# Supplementary material for: A quinolone N-oxide antibiotic selectively targets Neisseria gonorrhoeae via its toxin–antitoxin system
Source: Nat Microbiol. 2025 Apr 2;10(4):939–57. doi: 10.1038/s41564-025-01968-y (PMC11964940; doi:10.1038/s41564-025-01968-y)
Supplement: Supplementary file 1 — Supplementary Tables 1–5 and Data File 1. [file 41564_2025_1968_MOESM1_ESM.pdf]

nature microbiology

Supplementary information

https://doi.org/10.1038/s41564-025-01968-y

A quinolone *N*-oxide antibiotic selectively targets *Neisseria gonorrhoeae* via its toxin–antitoxin system

In the format provided by the authors and unedited

Suppl. Table S1. Raw data of neisserial growth curves depicted as heat map in Fig. 1C

The indicated Neisseria isolates were grown in liquid medium in the presence of 5, 10, 25, or 50 µM of NQ, trans-Δ1-NQ, NQNO or trans-Δ1-NQNO respectively. Control cultures were grown in the presence of solvent (1% DMSO). Growth was monitored by OD550 readings every 30 min and quantified by the area under curve (AUC) from growth curves. The AUC was normalized to growth curves obtained in the presence of solvent (1% DMSO). The values displayed here form the basis of the heat map shown in Fig. 1C.

	NQ				*trans*-Δ^1-NQ				NQNO				*trans*-Δ^1-NQNO			
µM	5	10	25	50	5	10	25	50	5	10	25	50	5	10	25	50
*Neisseria gonorrhoeae*_LSH1	0,12	0,12	0,01	0,08	0,02	0,03	0,03	0,03	0,01	0,01	0,00	0,01	0,81	0,03	0,01	0,02
Neisseria gonorrhoeae VP1/N131	0,15	0,13	0,15	0,09	0,21	0,10	0,01	0,01	0,02	0,00	0,02	0,00	0,92	0,25	0,00	0,01
Neisseria gonorrhoeae 81	0,04	0,02	0,00	0,00	0,05	0,11	0,00	0,00	0,02	0,00	0,00	0,00	0,13	0,05	0,00	0,00
Neisseria gonorrhoeae MS11	0,09	0,12	0,08	0,12	0,13	0,11	0,04	0,02	0,03	0,02	0,00	0,00	0,99	0,67	0,00	0,00
Neisseria gonorrhoeae 14	0,04	0,01	0,01	0,00	0,08	0,07	0,05	0,03	0,01	0,00	0,00	0,00	0,05	0,02	0,00	0,00
Neisseria lactamica	0,24	0,11	0,04	0,01	0,13	0,06	0,10	0,00	0,09	0,01	0,01	0,00	0,58	0,19	0,01	0,00
Neisseria gonorrhoeae 241	0,38	0,21	0,20	0,16	0,31	0,27	0,20	0,16	0,10	0,04	0,02	0,00	1,00	0,80	0,02	0,04
Neisseria gonorrhoeae 11	0,17	0,08	0,09	0,10	0,17	0,08	0,03	0,00	0,03	0,06	0,00	0,00	0,67	0,15	0,01	0,05
*Neisseria gonorrhoeae*_LSH3	0,62	0,45	0,24	0,19	0,68	0,57	0,33	0,18	0,05	0,01	0,00	0,00	0,89	0,39	0,00	0,04
*Neisseria gonorrhoeae*_LSH2	0,64	0,47	0,46	0,34	0,69	0,60	0,45	0,22	0,12	0,07	0,01	0,00	1,01	0,52	0,03	0,04
Neisseria gonorrhoeae 102	0,71	0,49	0,43	0,39	0,74	0,79	0,52	0,51	0,13	0,07	0,03	0,04	1,10	1,02	0,71	0,02
Neisseria gonorrhoeae 340	0,29	0,32	0,22	0,23	0,39	0,33	0,34	0,28	0,30	0,27	0,16	0,14	0,46	0,35	0,26	0,19
Neisseria mucosa	0,67	0,63	0,59	0,62	0,69	0,63	0,94	0,47	0,71	0,67	0,74	0,67	0,76	0,71	0,76	0,34
Neisseria macacae	0,91	0,91	0,87	0,85	0,85	0,85	0,86	0,87	0,78	0,74	0,76	0,75	0,78	0,65	0,49	0,55
Neisseria elongata elongata	0,88	0,63	0,69	0,02	0,80	0,85	0,45	0,39	0,69	0,72	0,64	0,66	0,64	0,51	0,68	0,36
Neisseria flavescens	0,20	0,15	0,06	0,03	0,39	0,40	0,30	0,23	0,94	0,92	0,79	0,52	1,00	0,85	0,51	0,11
Neisseria sicca	0,47	0,45	0,42	0,39	0,47	0,51	0,45	0,44	0,84	0,79	0,66	0,60	0,82	0,78	0,58	0,21
Neisseria canis	0,83	0,81	0,81	0,84	0,90	0,85	0,81	0,73	0,90	0,88	0,85	0,79	0,97	0,99	0,86	0,78
Neisseria dentiae	0,82	0,70	0,60	0,64	0,78	0,75	0,69	0,66	0,94	0,91	0,85	0,83	0,92	0,91	0,86	0,66
Neisseria subflava	0,37	0,29	0,24	0,24	0,93	0,54	0,37	0,14	1,04	0,95	0,82	0,86	1,02	1,04	0,93	0,33
Neisseria perflava	0,45	0,32	0,32	0,29	0,56	0,59	0,60	0,59	0,98	1,02	0,90	0,75	1,00	0,96	0,66	0,32
Neisseria cinerea	0,97	0,92	0,94	0,81	1,03	0,98	0,85	0,85	1,03	0,98	0,93	0,88	0,99	0,97	0,90	0,76

Suppl. Table S2. Results of genome comparison between parent *N. gonorrhoeae* MS11 strain versus NQNO-resistant strains derived from *N. gonorrhoeae* MS11

Comparative genomics of NQNO-sensitive *N. gonorrhoeae* MS11 and NQNO-resistant MS11-R1 and MS11-R2 revealed I) genes absent; II) missense mutations found; and III) plasmids missing in both NQNO-resistant strains compared to the parent, NQNO-sensitive MS11 strain.

I. Absent genes

Opacity protein opa54
IS1595 family transposase
Peptidase C39
Bacteriocin resistance protein
Hypothetical protein
IS1016 group transposase
TypeI restriction-modification system DNA methylase
Uncharacterized protein
Outer membrane protein P.IIC

II. Protein coding genes with missense mutations

01699:hypothetical protein
02115:hypothetical protein, phage associated
02116:hypothetical protein
02119 hypothetical protein
02231:transposase

III. Absent plasmids

pTetM (pEP5233), conjugative, Tet resistance

Suppl. Table S3. Origin of *Neisseria gonorrhoeae* strains used in this study

Name according to source	Description	Internal number	Scource	Isolation date	Isolation site	Publication
MS11	MS11 Opa+ (N309)	0009P	Thomas F. Meyer			[1]
	MS11 Opa- (N302)	0002P	Thomas F. Meyer			[1]
	MS11-R1	0568P	spontaneous NQNO-resistant			this study
	MS11-R2	0569P	spontaneous NQNO-resistant			this study
	MS11-R2 pTetM A	0566P	pTetM conjugant of MS11-R2			this study
	MS11-R2 pTetM B	0567P	pTetM conjugant of MS11-R2			this study
Ngo_LSH1	Clinical isolate	0022P	Dermatology	2002		
Ngo_LSH2	Clinical isolate	0023P	Dermatology	2002		
Ngo_LSH3	Clinical isolate	0024P	Dermatology	2002		
VP1/N131	VP1	0051P	Thomas F. Meyer			
NCTC13799	Clinical isolate	0098P	NCTC	2015, June, UK	throat	
Ngo 241	Clinical isolate DGI	0341P	Magnus Unemo	2001 August	blood	
Ngo 14	DGI	0342P	Magnus Unemo	2003 March	blood	
Ngo 102	DGI	0343P	Magnus Unemo	2005 March	blood	
Ngo 11	DGI	0344P	Magnus Unemo	2006 January	blood	
Ngo 81	DGI	0346P	Magnus Unemo	2009 March	joint fluid	
Ngo 340	DGI	0347P	Magnus Unemo	2012 August	joint fluid	
Ngo 316	Antibiotic resistant Austria	0348P	Magnus Unemo	2011 July	pharyngeal	[2]
Ngo XDR	Opa+ isolate of Ngo 316	0592P	visual selection of Ngo316			this study
Ngo 231	Antibiotic resistant Slovenia	0349P	Magnus Unemo	2011 October	pharyngeal	[3]
WHO F	NCTC 13477	0577P	NCTC	1991, Canada		[4]
WHO G	NCTC 13478	0578P	NCTC	1997, Thailand		[4]
WHO K	NCTC 13479	0579P	NCTC			[4]
WHO L	NCTC 13480	0580P	NCTC	1996, Asia		[4]
WHO M	NCTC 13481	0581P	NCTC	1992, Philippines		[4]
WHO N	NCTC 13482	0582P	NCTC	2001, Austrailia		[4]
WHO O	NCTC 13483	0583P	NCTC	1991, Canada		[4]
WHO P	NCTC 13484	0584P	NCTC			[4]
WHO P	WHO P TetM A	0575P	pTetM conjugant of WHO P			this study
	WHO P TetM A	0576P	pTetM conjugant of WHO P			this study
	WHO P ε/ζ	0743P	pNEISS ε1/ζ1 transformant			this study

Thomas F. Meyer, Max Planck Institute for Infection Biology, Berlin, Germany
Dermatology, Hautklinik, University of Würzburg, Würzburg, Germany
NCTC = National Collection of Type Cultures, Salisbury, United Kingdom
Magnus Unemo, Örebro University Hospital, Örebro, Sweden

[1] Edwards M, McDade RL, Schoolnik G, Rothbard JB, Gotschlich EC (1984) Antigenic analysis of gonococcal pili using monoclonal antibodies. *J Exp Med* 160, 1782-1791.

[2] Unemo M, Golparian D, Stary A, Eigentler A (2011) First *Neisseria gonorrhoeae* strain with resistance to cefixime causing gonorrhoea treatment failure in Austria, 2011. *Euro surveillance* 16.

[3] Unemo M, Golparian D, Potocnik M, Jeverica S (2012) Treatment failure of pharyngeal gonorrhoea with internationally recommended first-line ceftriaxone verified in Slovenia, September 2011. *Euro surveillance* 17.

[4] Unemo M, Fasth O, Fredlund H, Limnios A, Tapsall J (2009) Phenotypic and genetic characterization of the 2008 WHO Neisseria gonorrhoeae reference strain panel intended for global quality assurance and quality control of gonococcal antimicrobial resistance surveillance for public health purposes. *J Antimicrob Chemother* 63,1142-1151.

Suppl. Table S4. Origin of non-gonococcal bacterial strains used in this study

Name according to source	Number according to source	Internal number	Scource	Publication
N. mucosa	N350	0019P	Thomas F. Meyer	[5]
N. cinerea	N340	0020P	Thomas F. Meyer	[6]
N. lactamica	N348	0021P	Thomas F. Meyer	[6]
N. sicca	N349	0038P	Thomas F. Meyer	[6]
N. elongata elongata	DSM 17712	0123P	DSMZ	[7]
N. flavescens	DSM 17633	0124P	DSMZ	[8]
N. macacae	DSM 19175	0125P	DSMZ	[9]
N. perflava	DSM 18009	0126P	DSMZ	[8]
N. subflava	DSM 17610	0127P	DSMZ	[8]
Escherichia coli	EPEC	0026P	Thomas F. Meyer	
Escherichia coli	Nova Blue	360	Novagen (Merck KGaA)	
Klebsiella pneumoniae	Strain 3091	0234P	Tobias Oelschläger	
Lactobacillus gasseri	DSM 20243	0655P	DSMZ	
Lactobacillus jensenii	DSM 20557	0656P	DSMZ	
Lactobacillus paragasseri	DSM 20077	0657P	DSMZ	
Lactobacillus delbrueckii	DSM 0513	0658P	DSMZ	
Lactobacillus hominis	DSM 23910	0705P	DSMZ	
Limosilactobacillus vaginalis	DSM 5837	0704P	DSMZ	
P. aeruginosa PAO1	DSM 22644	0498P	DSMZ	
P. aeruginosa pqsL	PW 8104	0507P	UW Genome Sciences	[10, 11]
P. aeruginosa pqsH	PW 5343	0508P	UW Genome Sciences	[10, 11]
P. aeruginosa pqsR	PW 2812	0514P	UW Genome Sciences	[10, 11]

DSMZ, Leibniz-Institute DSZM-German Collection of Mircoorganisms and Cell Cultures, Braunschweig, Germany

UW Genome Sciences, Seattle, USA

Thomas F. Meyer, Max Planck Institute for Infection Biology, Berlin, Germany

Tobias Oelschläger, Institute for Molecular Infection Biology, Julius-Maximilian-Universität Würzburg, Germany

[5] Berger U. (1961) [Reduction of nitrate and nitrite by *Neisseria*]. *Zeitschrift fur Hygiene und Infektionskrankheiten; medizinische Mikrobiologie, Immunologie und Virologie*, 148, 45-50.

[6] Berger U. (1971) [*Neisseria mucosa var. heidelbergensis*]. *Zeitschrift fur medizinische Mikrobiologie und Immunologie* 156, 154-158.

[7] Bovre K, Holten E (1970) *Neisseria elongata sp.nov.*, a rod-shaped member of the genus *Neisseria*. Re-evaluation of cell shape as a criterion in classification. *J Gen Microbiol* 60, 67-75.

[8] Skerman VBD, McGowan V, Sneath PHA (1980) Approved Lists of Bacterial Names, vol. 30. USA: Int J. Syst. Bacteriol.

[9] Vedros NAH, C.; Chun, P. (1983) *Neisseria macacae sp. nov*., a new *Neisseria* species isolated from the Oropharynges of Rhesus Monkeys (*Macaca mulatta*). *Int J Syst Bact* 33, 515-520.

[10] Jacobs MA, Alwood A, Thaipisuttikul I, Spencer D, Haugen E, Ernst S, Will O, Kaul R, Raymond C, Levy R *et al* (2003) Comprehensive transposon mutant library of *Pseudomonas aeruginosa*. *Proc Natl Acad Sci U S A* 100, 14339-14344.

[11] Held K, Ramage E, Jacobs M, Gallagher L, Manoil C (2012) Sequence-verified two-allele transposon mutant library for Pseudomonas aeruginosa PAO1. *J Bacteriol* 194, 6387-6389

Suppl. Table S5. Overview of synthetic compound names, abbreviations and structures.

Name	Abbreviation	Structure
6-Chloro-2-nonylquinolin-4(1*H*)-one	**6Cl-NQ**	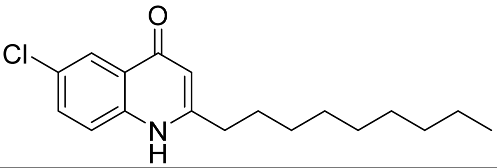
6-Bromo-2-nonylquinolin-4(1*H*)-one	**6Br-NQ**	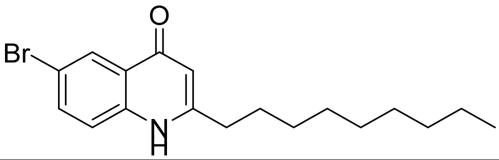
6-Fluoro-2-nonylquinolin-4(1*H*)-one	**6F-NQ**	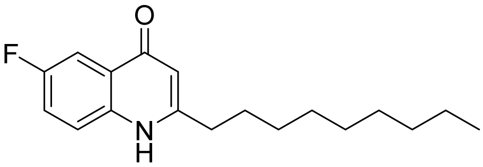
6-Methyl-2-nonylquinolin-4(1*H*)-one	**6Me-NQ**	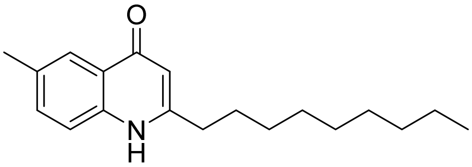
6-Methoxy-2-nonylquinolin-4(1*H*)-one	**6OMe-NQ**	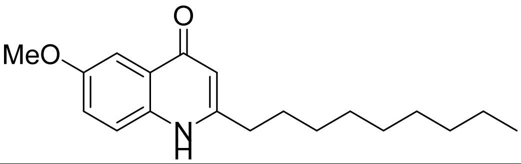
2-Nonyl-6-(trifluoromethyl)quinolin-4(1*H*)-one	**6CF$_3$-NQ**	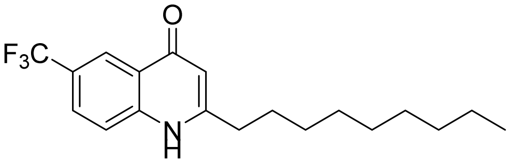
2-Nonyl-6-(trifluoromethoxy)quinolin-4(1*H*)-one	**6OCF$_3$-NQ**	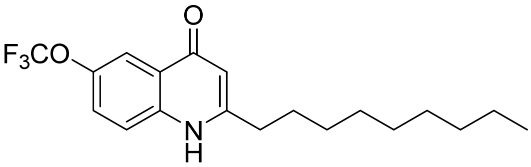
5-Methyl-2-nonylquinolin-4(1*H*)-one	**5Me-NQ**	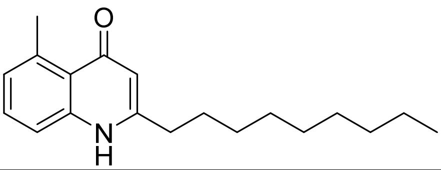
7-Methyl-2-nonylquinolin-4(1*H*)-one	**7Me-NQ**	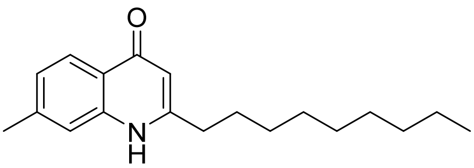
7-Fluoro-2-nonylquinolin-4(1*H*)-one	**7F-NQ**	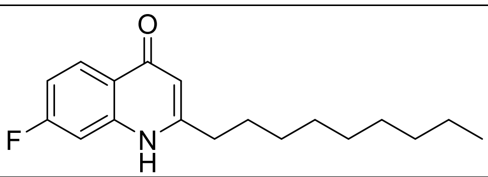

3-Methyl-2-nonylquinolin-4(1*H*)-one	**3Me-NQ**	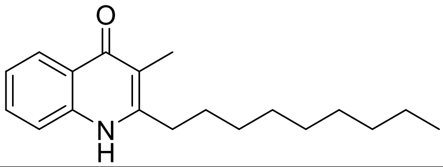
5,8-dimethyl-2-nonylquinolin-4(1*H*)-one	**5,8diMe-NQ**	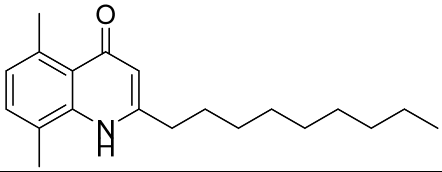
8-Methyl-2-nonylquinolin-4(1*H*)-one	**8Me-NQ**	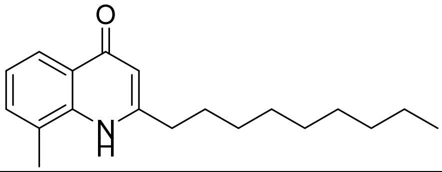
8-Fluoro-2-nonylquinolin-4(1*H*)-one	**8F-NQ**	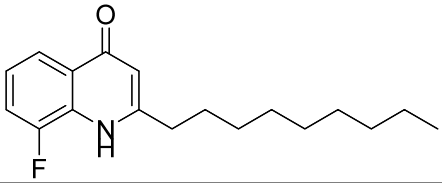
2-nonyl-1,8-naphthyridin-4(1*H*)-one	**C8Py-NQ**	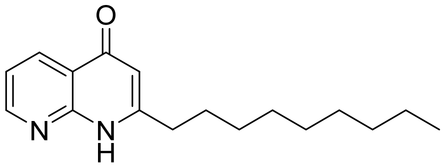
6-hydroxy-2-nonylquinolin-4(1*H*)-one	**6OH-NQ**	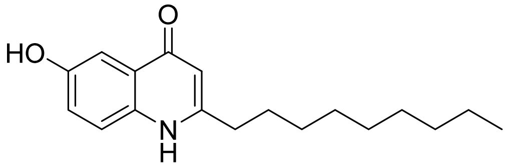
6-Chloro-1-hydroxy-2-nonylquinolin-4(1*H*)-one	**6Cl-NQNO**	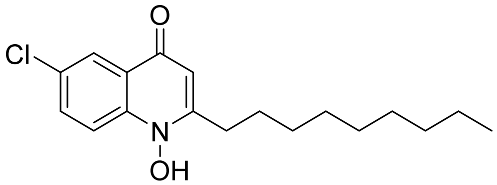
6-Bromo-1-hydroxy-2-nonylquinolin-4(1*H*)-one	**6Br-NQNO**	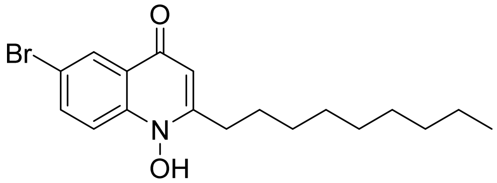
6-Fluoro-1-hydroxy-2-nonylquinolin-4(1*H*)-one	**6F-NQNO**	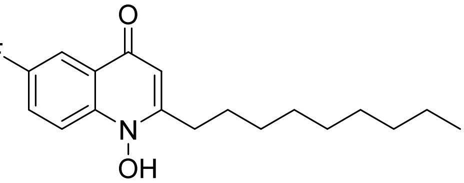
1-Hydroxy-6-methyl-2-nonylquinolin-4(1*H*)-one	**6Me-NQNO**	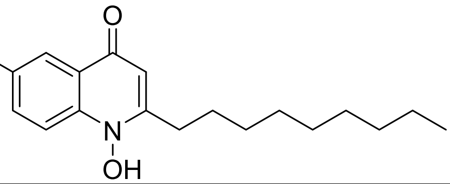

1-Hydroxy-6-methoxy-2-nonylquinolin-4(1*H*)-one	**6OMe-NQNO**	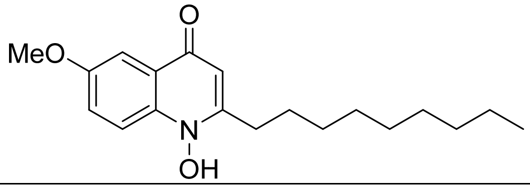
1-Hydroxy-2-nonyl-6-(trifluoromethyl)quinolin-4(1*H*)-one	**6CF$_3$-NQNO**	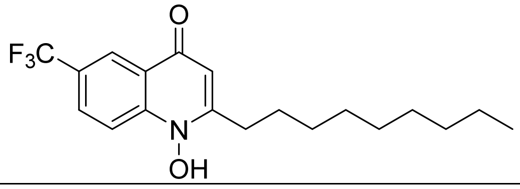
1-Hydroxy-2-nonyl-6-(trifluoromethoxy)quinolin-4(1*H*)-one	**6OCF$_3$-NQNO**	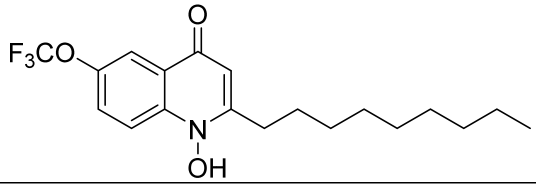
1-Hydroxy-5-methyl-2-nonylquinolin-4(1*H*)-one	**5Me-NQNO**	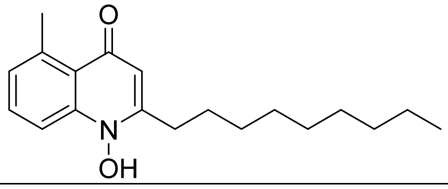
7-Fluoro-1-hydroxy-2-nonylquinolin-4(1*H*)-one	**7F-NQNO**	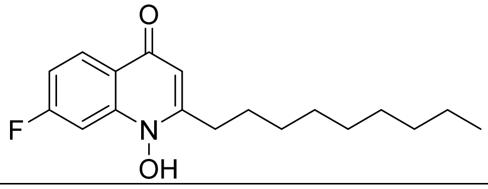
1-Hydroxy-3-methyl-2-nonylquinolin-4(1*H*)-one	**3Me-NQNO**	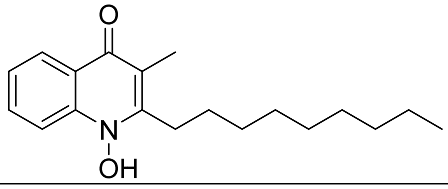
1,6-dihydroxy-2-nonylquinolin-4(1*H*)-one	**6OH-NQNO**	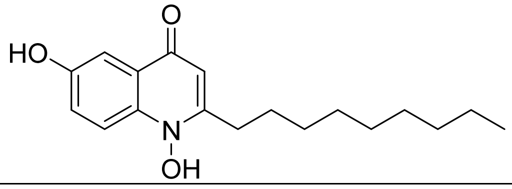

Supplementary Data File 1

Synthesis, structures and NMR-based quality control of AQ compounds

1. Materials and Methods

The solvents and chemicals for synthesis were purchased from Sigma-Aldrich, Acros Organics, Carl Roth, VWR Chemicals, Merck, and TCI chemicals and were used without further purification. For Silica gel chromatography, distilled technical grade solvents and silica gel 60 A (Carl Roth) was used. Thin layer chromatography (TLC) was performed using aluminium sheets "TLC Silica gel 60 F_254" from Merck Millipore and analysed with UV light or by permanganate staining. NMR spectra were obtained with Bruker Avance-III 400 and Bruker Avance-III 600 NMR spectrometers at ambient temperature. Multiplicities are given as follows: s-singlet, d-doublet, t-triplet, q-quarter, m-multiplet. Chemical shifts (δ) are given in parts per million (ppm) relative to the solvent residual signal with $CDCl_3$ -d (δ_H = 7.26 ppm and δ_C = 77.16 ppm), DMSO-*d*$_6$ (δ_H = 2.50 ppm and δ_C = 39.52 ppm), or MeOD-*d*$_4$ (δ_H = 3.31 ppm and δ_C = 49.00 ppm). The data obtained were processed and analysed with MestReNova 12.0 software. High resolution mass spectrometry data were obtained on an ESI-Orbitrap (Thermo Scientific, LTQ Orbitrap Velos) by direct injection and analysed with Xcalibur (Thermo Scientific) software.

2. Syntheses

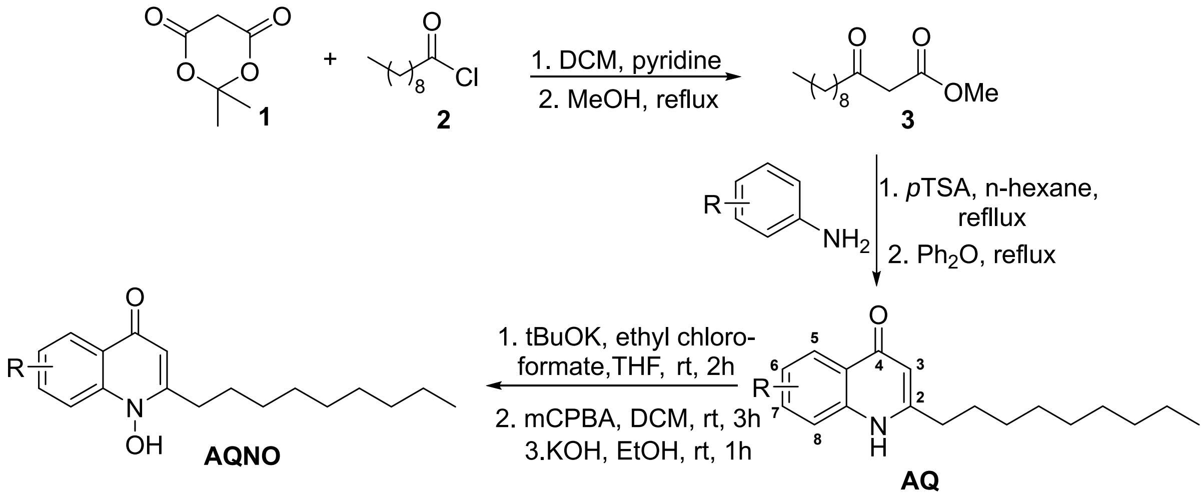

Synthesis of methyl-3-oxododecanoate 3

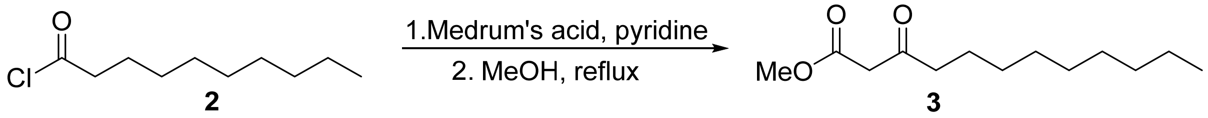

2,2-Dimethyl-1,3-dioxane-4,6-dione (Meldrum's acid) (4.45 g, 30.0 mmol, 1 eq.) was dissolved in 50 mL anhydrous DCM and cooled to 0°C. Pyridine (6.22 mL, 77.2 mmol, 2.5 eq.) was added and the reaction stirred for 30 min at 0°C. Decanoyl chloride (6.41 mL, 30.9 mmol, 1eq.) was added dropwise and the resulting orange/red solution was allowed to stir at 0°C for 1 h and at room temperature for 1 h. After the reaction time, the mixture was washed with 100 mL of 1M HCl. The layers were separated, and the aqueous phase was back-extracted three times with 20 mL of DCM. The combined organic layers were washed with 50 mL of 1M HCl followed by 30 mL of brine. The orange solution was dried over magnesium sulfate, filtered, and concentrated to give the desired adduct as an orange oil. The oil was dissolved in 50 mL of anhydrous MeOH and heated at reflux for 5 h. The solvent was evaporated, and the remaining oil was purified by column chromatography with silica gel 60 and hexane/ethyl acetate 9:1 to yield the desired product as the yellow oil.

Methyl 3-oxododecanoate **3**: 82% R_f = 0.50 (Hexane/EtOAc 8:1)
^{1}H NMR (400 MHz, $CDCl_3$-*d*) δ 3.71 (s, 3H, -OC**H**$_3$), 3.44 (s, 2H, -C**H**$_2$-CO-), 2.50 (t, *J* = 7.4 Hz, 2H, -CO-C**H**$_2$-), 1.63 – 1.48 (m, 2H, -CO-CH$_2$-C**H**$_2$), 1.34 – 1.15 (m, 12H, -(C**H**$_2$)$_6$CH$_3$), 0.85 (t, *J* = 6.6 Hz, 3H, -C**H**$_3$).

^{13}C NMR (101 MHz, $CDCl_3$-*d*) δ 202.9 (-**C**O), 167.8 (-**C**OOMe), 52.5 (-COO**Me**), 49.1 (COOMe-**C**H$_2$-CO), 43.2 (-CO-**C**H$_2$-), 32.0, 29.51, 29.5, 29.4, 29.1, 23.6, 22.8 (-(CH$_2$)$_7$**C**H$_3$), 14.1 (-**C**H$_3$).

Synthesis of methyl 2-methyl-3-oxododecanoate

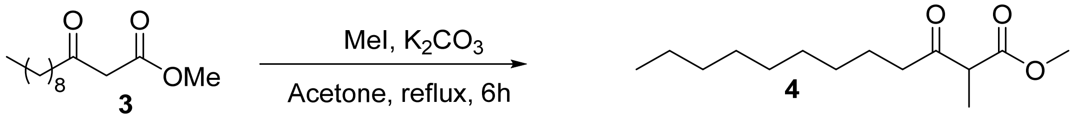

To the round-bottom flask containing dry potassium carbonate (0.969 g, 7 mmol, 2 eq.) was added a solution of methyl 3-oxododecanote (800 mg, 3.5 mmol, 1 eq.) in acetone (12.5 mL). The resulting mixture was allowed to stir for 20 min before the addition of methyl iodide (241 µL, 3.8 mmol, 1.1 eq.). The reaction mixture was allowed to stir under reflux for 6 h. The mixture was cooled to room temperature and the solvent was removed in vacuo to yield crude product as the colourless oil, which was used in the next step without further purification.

^{1}H NMR (400 MHz, $CDCl_3$-*d*) δ 3.72 (s, 3H, - OC**H**$_3$), 3.56 – 3.47 (m, 1H, -C**H**-CO-), 2.61 – 2.40 (m, 2H, -CO-C**H**$_2$-), 1.63 – 1.52 (m, 2H, -CO-CH$_2$-C**H**$_2$), 1.37 – 1.19 (m, 15H, -(C**H**$_2$)$_6$CH$_3$, -CO-CH(C**H**$_3$)-CO-), 0.87 (t, *J* = 6.8 Hz, 3H, -C**H**$_3$).

^{13}C NMR (101 MHz, $CDCl_3$-*d*) δ 206.10 (-**C**O), 171.24 (-**C**OOMe), 52.83 (-COO**Me**), 52.47 (COOMe-**C**H-CO), 41.53 (-CO-**C**H$_2$-), 31.99, 29.55, 29.51, 29.39, 29.19, 23.68, 22.79 (-(CH$_2$)$_7$**C**H$_3$), 14.22 (-**C**H$_3$), 12.99 (-CO-CH(**C**H$_3$)-CO-).

General synthesis of quinoline-4-ones

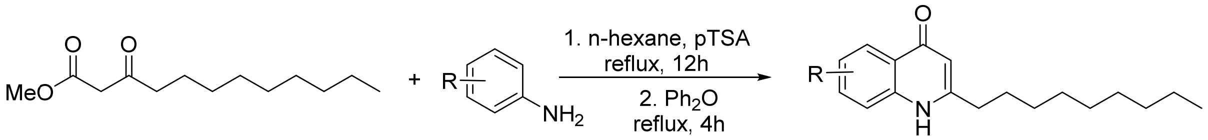

The β-ketoester (1 eq.) was dissolved in 20 mL anhydrous *n*-hexane with 1 eq. of aniline derivatives and 2 mol% pTSA. 2 g molecular sieve 4 Å was added and the mixture was refluxed for 12 h. After cooling down to room temperature, the reaction mixture was filtered to remove molecular sieve 4 Å, and the solvent was evaporated to give the desired product as a yellow oil. The oil was dissolved in diphenyl ether (10 mL/1 g educt) and the mixture was refluxed for 4 h. The reaction mixture was cooled to room temperature, and dropwise added to *n*-hexane. The precipitate was filtered and washed with *n*-hexane several time to obtain the desired products as the solid.

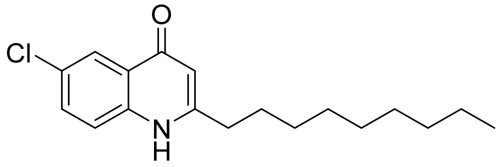

6-Chloro-2-nonylquinolin-4(1*H*)-one (**6Cl-NQ**): obtained from 4-chloroaniline and methyl-3-oxododecanoate **3** (55% over 2 steps)

^{1}H NMR (400 MHz, DMSO-*d6*) δ 11.65 (s, 1H, NH), 8.03 – 7.91 (m, 1H, H-5), 7.65 (d, *J* = 8.8 Hz, 1H, H-7), 7.57 (d, *J* = 8.8 Hz, 1H, H-8), 5.97 (s, 1H, H-3), 2.59 (t, *J* = 7.7 Hz, 2H, H-9), 1.75 – 1.57 (m, 2H, H-10), 1.27 (d, *J* = 25.3 Hz, 12H, H-11-16), 0.84 (t, *J* = 6.7 Hz, 3H, H-17).

^{13}C NMR (101 MHz, DMSO-*d6*) δ 175.41 (C-4), 154.18 (C-2), 138.39 (C-8a), 131.57 (C-7), 127.42 (C-6), 125.50 (C-5), 123.69 (C-4a), 120.35 (C-8), 107.82 (C-3), 33.23 (C-9), 31.22, 28.83, 28.67, 28.61, 28.45, 28.22, 22.05 (C-10-16), 13.91 (**C**H_3).

TOF-HRMS: m/z = 306.1607 $[M+H]^+$, calc. for $C_{18}H_{24}ClNO + H^+$ = 306.1618; 328.1428 $[M+Na]^+$, calc. for $C_{18}H_{24}ClNO + Na^+$ = 328.1438

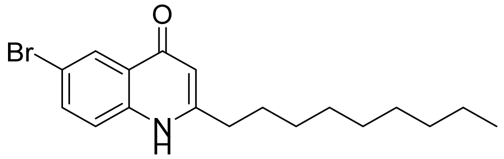

6-Bromo-2-nonylquinolin-4(1H)-one (**6Br-NQ**): obtained from 4-bromoaniline and methyl-3-oxododecanoate **3** (43% after 2 steps)

^{1}H NMR (400 MHz, DMSO-*d6*) δ 11.63 (s, 1H, NH), 8.11 (d, J = 2.3 Hz, 1H, H-5), 7.75 (dd, J = 8.8, 2.4 Hz, 1H, H-8), 7.50 (d, J = 8.8 Hz, 1H, H-7), 5.97 (s, 1H, H-3), 2.63 – 2.54 (m, 2H, H-9), 1.65 (p, J = 7.2 Hz, 2H, H-10), 1.38-1.15 (m, 12H, H-11-16), 0.90 – 0.78 (m, 3H, H-17).

^{13}C NMR (101 MHz, DMSO-*d6*) δ 175.4 (C-4), 154.2 (C-2), 139.0 (C-8a), 134.2 (C-7), 126.9 (C-5), 126.0 (C-4a), 120.5 (C-6), 115.4 (C-8), 107.9 (C-3), 33.2 (C-9), 31.2, 28.8, 28.7, 28.6, 28.4, 28.2, 22.0 (C-10-16), 13.9 (C-17).

TOF-HRMS: m/z = 350.1108 [M+H]$^+$, calc. for $C_{18}H_{24}BrNO + H^+$ = 350.1138; 372.0922 [M+Na]$^+$, calc. for $C_{18}H_{24}BrNO + Na^+$ = 372.0933

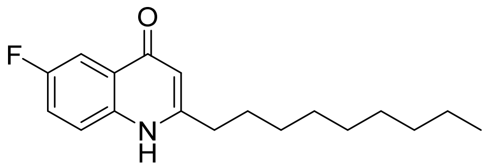

6-Fluoro-2-nonylquinolin-4(1*H*)-one (**6F-NQ**): obtained from 4-fluoroaniline and methyl-3-oxododecanoate **3** (64% over 2 steps)

^{1}H NMR (400 MHz, DMSO-d_6) δ 11.61 (s, 1H, NH), 7.67 (dd, *J* = 9.4, 3.0 Hz, 1H, H-5), 7.60 (dd, *J* = 9.1, 4.7 Hz, 1H, H-8), 7.52 (td, *J* = 8.6, 3.0 Hz, 1H, H-7), 5.93 (s, 1H, H-3), 2.62 – 2.54 (m, 2H, H-9), 1.66 (p, *J* = 7.2 Hz, 2H, H-10), 1.37 – 1.17 (m, 12H, H-11-16), 0.84 (t, *J* = 6.8 Hz, 3H, H-17).

^{13}C NMR (101 MHz, DMSO-d_6) δ 175.9 (C-4), 158.1 (d, *J* = 242.2 Hz, C-6), 153.8 (C-2), 136.8 (C-8a), 125.7d, *J* = 6.1 Hz, C-8), 120.6 (d, *J* = 8.1 Hz, C-4a), 120.2 (d, *J* = 26.3 Hz, C-7), 108.7 (d, *J* = 21.2 Hz, C-5), 106.9 (C-3), 33.2 (C-9), 31.2, 28.8, 28.7, 28.6, 28.4, 28.3, 22.0 (C-10-16), 13.9 (C-17).

TOF-HRMS: m/z = 290.1902 [M+H]$^+$, calc. for $C_{18}H_{24}FNO + H^+$ = 290.1911; 312.1719 [M+Na]$^+$, calc. for $C_{18}H_{24}FNO + Na^+$ = 312.1734

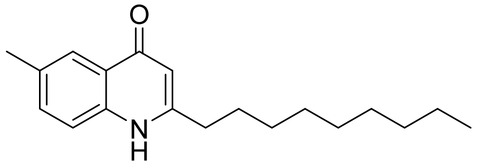

6-Methyl-2-nonylquinolin-4(1*H*)-one (**6Me-NQ**): obtained from 4-methylaniline and methyl-3-oxododecanoate **3** (54% over 2 steps)

^{1}H NMR (400 MHz, DMSO-d_6) δ 11.40 (s, 1H, NH), 7.82 (s, 1H, H-5), 7.43 (s, 2H, H-7, H-8), 5.88 (s, 1H, H-3), 2.56 (t, *J* = 7.7 Hz, 2H, H-9), 2.38 (s, 3H, Ar-Me), 1.64 (q, *J* = 7.7 Hz, 2H, H-10), 1.41 – 1.13 (m, 12H, H-11-16), 0.84 (t, *J* = 6.8 Hz, 3H, H-17).

^{13}C NMR (101 MHz, DMSO-d_6) δ 176.50 (C-2), 153.07 (C-4), 138.17 (C-8a), 132.68 (C-6), 131.82 (C-7), 124.49 (C-4a), 123.97 (C-5), 117.74 (C-8), 107.27 (C-3), 33.17 (C-9), 31.18, 28.80, 28.64, 28.57, 28.43, 28.27, 22.00 (C-10-16), 20.64 (Ar-Me), 13.86 (C-17).

TOF-HRMS: m/z = 286.2154 [M+H]$^+$, calc. for $C_{19}H_{27}NO + H^+$ = 286.2166; 308.1970 [M+Na]$^+$, calc. for $C_{19}H_{27}NO + Na^+$ = 308.1985

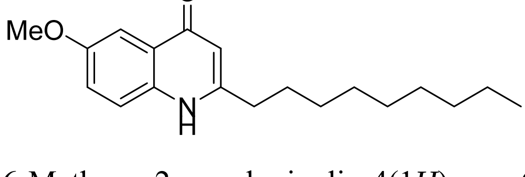

6-Methoxy-2-nonylquinolin-4(1*H*)-one (**6OMe-NQ**): obtained from 4-methoxylaniline and methyl-3-oxododecanoate **3** (68% after 2 steps)

^{1}H NMR (400 MHz, DMSO-d_6) δ 11.43 (s, 1H, NH), 7.48 (d, *J* = 9.0 Hz, 1H, H-5), 7.45 (d, *J* = 2.9 Hz, 1H, H-8), 7.25 (dd, *J* = 9.0, 3.0 Hz, 1H, H-7), 5.88 (s, 1H, H-3), 3.81 (s, 3H, Ar-O**Me**), 2.60 – 2.52 (m, 2H, H-9), 1.65 (p, *J* = 7.1 Hz, 2H, H-10), 1.36 – 1.17 (m, 12H, H-11-16), 0.89 – 0.79 (m, 3H, H-17).

^{13}C NMR (101 MHz, DMSO-d_6) δ 176.2 (C-4), 155.2 (C-2), 152.5 (C-6), 134.7 (C-8a), 125.6 (C-4a), 121.7 (C-7), 119.6 (C-8), 106.6 (C-5), 104.2 (C-3), 55.3 (Ar-O**Me**), 33.2 (C-9), 31.2, 28.8, 28.7, 28.6, 28.5, 28.4, 22.0 (C-10-16), 13.1 (C-17).

TOF-HRMS: m/z = 302.2101 $[M+H]^+$, calc. for $C_{19}H_{27}NO_2 + H^+$ = 302.2115; 324.1923 $[M+Na]^+$, calc. for $C_{19}H_{27}NO_2 + Na^+$ = 324.1934

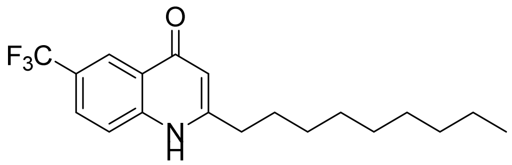

2-Nonyl-6-(trifluoromethyl)quinolin-4(1*H*)-one (**6CF$_3$-NQ**): obtained from 4-(trifluoromethyl)aniline and methyl-3-oxododecanoate **3** (20% over 2 steps)

^{1}H NMR (400 MHz, DMSO-d_6) δ 11.80 (s, 1H, NH), 8.33 – 8.27 (m, 1H, H-5), 7.91 (dd, *J* = 8.8, 2.2 Hz, 1H, H-7), 7.72 (d, *J* = 8.7 Hz, 1H, H-8), 6.04 (d, *J* = 1.5 Hz, 1H, H-3), 2.65 – 2.57 (m, 2H, H-9), 1.67 (p, *J* = 7.4 Hz, 2H, H-10), 1.40-1.15 (m,12H, H-11-16), 0.90 – 0.78 (m, 3H, H-17).

^{13}C NMR (101 MHz, DMSO-d_6) δ 176.2 (C-4), 154.8 (C-2), 142.3 (C-8a), 127.5 (C-7), 123.8 (C-5), 123.2 (C-4a), 122.9 (Ar-**C**F$_3$), 122.4 (C-6), 119.5 (C-8), 108.7 (C-3), 33.2 (C-9), 31.2, 28.8, 28.7, 28.6, 28.4, 28.1, 22.0 (C-10-16), 13.9 (C-17).

TOF-HRMS: m/z = 340.1883 $[M+H]^+$, calc. for $C_{19}H_{24}F_3NO + H^+$ = 340.1871; 362.1702 $[M+Na]^+$, calc. for $C_{19}H_{24}F_3NO + Na^+$ = 362.1691

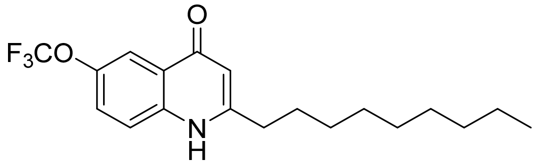

2-Nonyl-6-(trifluoromethoxy)quinolin-4(1*H*)-one (**6OCF$_3$-NQ**): obtained from 4-(trifluoromethoxy)aniline and methyl-3-oxododecanoate **3** (40% over 2 steps)

^{1}H NMR (400 MHz, DMSO-d_6) δ 11.72 (s, 1H, NH), 7.87 (s, 1H, H-5), 7.68 – 7.60 (m, 2H, H-7, H-8), 5.98 (s, 1H, H-3), 2.63 – 2.56 (m, 2H, H-9), 1.66 (p, *J* = 7.3 Hz, 2H, H-10), 1.35-1.19 (m, 12H, H-11-16), 0.87 – 0.80 (m, 3H, H-17).

^{13}C NMR (101 MHz, DMSO-d_6) δ 175.9 (C-4), 154.4 (C-2), 145.5 (C-6), 143.71 (C-4a), 138.8 (Ar-O**C**F$_3$), 125.2 (C-4a), 125.1 (C-7), 120.6 (C-8), 115.8 (C-5), 107.6 (C-3), 33.2 (C-9), 31.2, 28.8, 28.7, 28.6, 28.4, 28.2, 22.0 (C-10-16), 13.9 (C-17).

TOF-HRMS: m/z = 356.1820 $[M+H]^+$, calc. for $C_{19}H_{24}F_3NO_2$+ H^+ = 356.1832; 378.1639 $[M+Na]^+$, calc. for $C_{19}H_{24}F_3NO_2$+ Na^+ = 378.1651

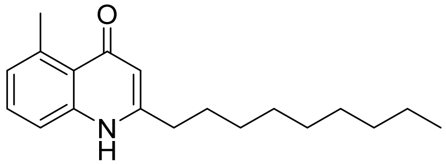

5-Methyl-2-nonylquinolin-4(1*H*)-one (**5Me-NQ**): obtained from 3-methylaniline and methyl-3-oxododecanoate **3** (32% over 2 steps)

^{1}H NMR (400 MHz, DMSO-d_6) δ 11.17 (s, 1H, NH), 7.42 – 7.35 (m, 1H, H-7), 7.32 (d, *J* = 8.1 Hz, 1H, H-8), 6.93 (d, *J* = 7.1 Hz, 1H, H-5), 5.80 (s, 1H, H-3), 2.77 (s, 3H, Ar-**Me**), 2.50 (dt, *J* = 4.1, 2.0 Hz, 2H, H-9 mix with DMSO), 1.63 (q, *J* = 6.7 Hz, 2H, H-10), 1.36-1.11 (m, 12H, H-11-16), 0.84 (t, *J* = 6.7 Hz, 3H, H-17).

^{13}C NMR (101 MHz, DMSO-d_6) δ 179.5 (C-4), 151.7 (C-2), 141.8 (C-8a), 139.0 (C-5), 130.4 (C-7), 125.0 (C-6), 122.9 (C-4a), 116.0 (C-8), 109.4 (C-3), 32.6 (C-9), 31.2, 28.9, 28.7, 28.6, 28.4, 28.1 (C-10-15), 23.1 (Ar-**Me**), 22.0 (C-16), 13.9 (C-17).

TOF-HRMS: m/z = 289.2156 $[M+H]^+$, calc. for $C_{19}H_{27}NO + H^+$ = 286.2166; 308.1972 $[M+Na]^+$, calc. for $C_{19}H_{27}NO + Na^+$ = 308.1985

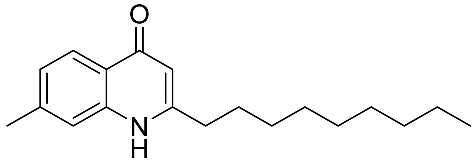

7-Methyl-2-nonylquinolin-4(1*H*)-one (**7Me-NQ**): obtained from 3-methylaniline and methyl-3-oxododecanoate **3** (40% over 2 steps)

^{1}H NMR (400 MHz, DMSO-d_6) δ 11.66 (s, 1H, NH), 7.90 (d, *J* = 8.2 Hz, 1H, H-5), 7.37 (s, 1H, H-8), 7.08 (d, *J* = 8.3 Hz, 1H, H-6), 5.84 (s, 1H, H-3), 2.57 (t, *J* = 7.6 Hz, 2H, H-9), 2.40 (s, 3H, Ar-**Me**), 1.65 (p, *J* = 6.7 Hz, 2H, H-10), 1.36 – 1.15 (m, 12H, H-11-16), 0.84 (t, *J* = 6.7 Hz, 3H, H-17).

^{13}C NMR (101 MHz, DMSO-d_6) δ 176.7 (C-4), 153.3 (C-2), 141.3 (C-8a), 140.4 (C-7), 124.7 (C-5), 124.3 (C-4a), 122.6 (C-6), 117.2 (C-8), 107.4 (C-3), 33.1 (C-9), 31.2, 28.9, 28.7, 28.6, 28.4, 28.3, 22.1 (C-10-16), 21.3 (Ar-**Me**), 13.9 (C-17).

TOF-HRMS: m/z = 286.2154 $[M+H]^+$, calc. for $C_{19}H_{27}NO + H^+$ = 286.2166; 308.1970 $[M+Na]^+$, calc. for $C_{19}H_{27}NO + Na^+$ = 308.1985

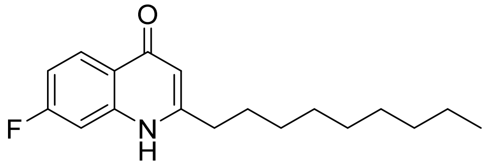

7-Fluoro-2-nonylquinolin-4(1*H*)-one (**7F-NQ**): obtained from 3-fluoroaniline and methyl-3-oxododecanoate **3** (41% over 2 steps)

^{1}H NMR (400 MHz, DMSO-d_6) δ 11.51 (s, 1H, NH), 8.07 (dd, *J* = 8.9, 6.5 Hz, 1H, H-5), 7.23 (dd, *J* = 10.2, 2.4 Hz, 1H, H-8), 7.12 (td, *J* = 8.8, 2.5 Hz, 1H, H-6), 5.91 (s, 1H, H-3), 2.60 –

2.53 (m, 2H, H-9), 1.65 (p, *J* = 7.3 Hz, 2H, H-10), 1.37 – 1.16 (m, 12H, H-11-16), 0.89 – 0.79 (m, 3H, H-17).

^{13}C NMR (101 MHz, DMSO-*d*6) δ 176.2 (C-4), 163.6 (d, *J* = 248.5 Hz, C-7), 154.1 (C-2), 141.5 (d, *J* = 13.1 Hz, C-8a), 128.1 (d, *J* = 10.1 Hz, C-5), 121.6 (C-4a), 111.5 (d, *J* = 24.2 Hz, C-6), 107.9 (C-3), 103.0 (d, *J* = 25.3 Hz, C-8), 33.2 (C-9), 31.2, 28.8, 28.7, 28.6, 28.4, 28.1, 22.0 (C-10-16), 13.9 (C-17).

TOF-HRMS: m/z = 290.1902 [M+H]$^+$, calc. for $C_{18}H_{24}FNO$ + H^+ = 290.1914; 312.1718 [M+Na]$^+$, calc. for $C_{18}H_{24}FNO$ + Na^+ = 312.1734

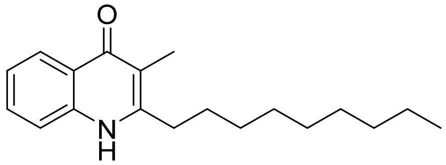

3-Methyl-2-nonylquinolin-4(1*H*)-one (**3Me-NQ**): obtained from aniline and methyl 2-methyl-3-oxododecanoate **4 (**60% after 2 steps)

^{1}H NMR (400 MHz, CDCl3-*d*) δ 11.72 (s, 1H, NH), 8.39 (d, *J* = 8.2 Hz, 1H, H-5), 7.77 (d, *J* = 8.4 Hz, 1H, H-7), 7.53 (t, *J* = 7.6 Hz, 1H, H-8), 7.29 (d, *J* = 7.6 Hz, 1H, H-6), 2.85 – 2.72 (m, 2H, H-9), 2.22 (s, 3H, -C**H**3), 1.66 (p, *J* = 7.7 Hz, 2H, H-10), 1.37-1.09 (m, 12H, H-11-16), 0.85 (t, *J* = 6.9 Hz, 3H, H-17).

^{13}C NMR (101 MHz, CDCl3-*d*) δ 177.8 (C-4), 151.7 (C-2), 139.7 (C-8a), 131.2 (C-7), 125.6 (C-5), 123.6 (C-4a), 123.3 (C-6), 118.3 (C-8), 115.2 (C-3), 33.0 (C-9), 32.0, 29.7, 29.6, 29.5, 29.4, 29.1, 22.8 (C-10-16), 14.2 (C-17), 11.0 (**C**H3).

TOF-HRMS: m/z = 286.2151 [M+H]$^+$, calc. for $C_{19}H_{27}NO$ + H^+ = 286.2166; 308.1967[M+Na]$^+$, calc. for $C_{19}H_{27}NO$ + Na^+ = 308.1985

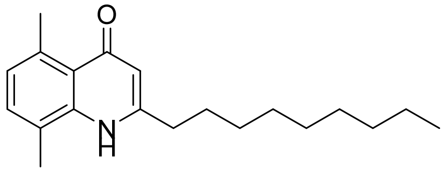

5,8-dimethyl-2-nonylquinolin-4(1*H*)-one (**5,8diMe-NQ**): obtained from 2,5-dimethylaniline and methyl-3-oxododecanoate **3 (**70% after 2 steps)

^{1}H NMR (400 MHz, DMSO-*d*6) δ 9.80 (s, 1H, NH), 7.25 (d, *J* = 7.4 Hz, 1H, H-7), 6.85 (d, *J* = 7.4 Hz, 1H, H-6), 5.83 (s, 1H, H-3), 2.73 (s, 3H, Ar-**Me** H-5), 2.67 – 2.56 (m, 2H, H-9), 2.44 (s, 3H, Ar-**Me**, H-8), 1.62 (p, *J* = 7.3 Hz, 2H, H-10), 1.41-1.04 (m, 12H, H-11-16), 0.84 (t, *J* = 6.7 Hz, 3H, H-17).

^{13}C NMR (101 MHz, DMSO-*d*6) δ 179.8 (C-4), 152.2 (C-2), 140.2 (C-8a), 136.6 (C-5), 131.5 (C-7), 124.8 (C-8), 123.4 (C-4a), 123.1 (C-6), 109.7 (C-3), 32.4 (C-9), 31.2, 28.9, 28.8, 28.64 (2C), 28.58 (C-10-15), 23.3 (Ar-**Me** C-5), 22.0 (C-16), 17.8 (Ar-**Me** C-8), 13.9 (C-17).

TOF-HRMS: m/z =300.2307 [M+H]$^+$, calc. for $C_{20}H_{29}NO$ + H^+ = 300.2322; 322.2128 [M+Na]$^+$, calc. for $C_{20}H_{29}NO$ + Na^+ = 322.2141

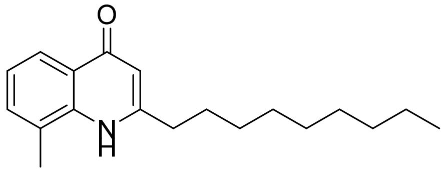

8-Methyl-2-nonylquinolin-4(1*H*)-one (**8Me-NQ**): obtained from 2-methylaniline and methyl-3-oxododecanoate **3** (67% after 2 steps)

^{1}H NMR (400 MHz, DMSO-d_6) δ 10.34 (s, 1H, NH), 7.93 (d, *J* = 8.0 Hz, 1H, H-5), 7.46 (d, *J* = 7.0 Hz, 1H, H-7), 7.18 (t, *J* = 7.6 Hz, 1H, H-6), 5.98 (s, 1H, H-3), 2.70 (t, *J* = 7.7 Hz, 2H, H-9), 2.52 (s, 3H, Ar-**Me**), 1.65 (p, *J* = 7.1 Hz, 2H, H-10), 1.41-1.14 (m, 12H, H-11-16), 0.84 (t, *J* = 6.3 Hz, 3H, H-17).

^{13}C NMR (151 MHz, DMSO-d_6) δ 176.8 (C-4), 154.4 (C-2), 138.8 (C-8a), 132.5 (C-7), 126.1 (C-8), 124.7 (C-4a), 122.6 (C-5), 118.6 (C-6), 107.7 (C-3), 33.0 (C-9), 31.3, 28.9 (2C), 28.8, 28.68, 28.66, 22.1 (C-10-16), 17.7 (Ar-**Me**), 13.9 (C-17).

TOF-HRMS: m/z =286.2154 $[M+H]^+$, calc. for $C_{19}H_{27}NO + H^+$ = 286.2166; 308.1969 $[M+Na]^+$, calc. for $C_{19}H_{27}NO + Na^+$ = 308.1985

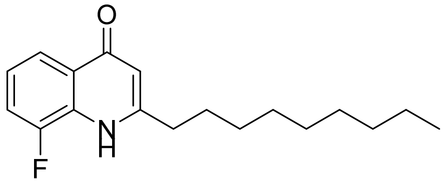

8-Fluoro-2-nonylquinolin-4(1*H*)-one (**8F-NQ**): obtained from 2-fluoroaniline and methyl-3-oxododecanoate **3** (46% over 2 steps)

^{1}H NMR (400 MHz, DMSO-d_6) δ 11.43 (s, 1H, NH), 7.85 (d, *J* = 8.0 Hz, 1H, H-5), 7.53 (ddd, *J* = 11.4, 7.9, 1.2 Hz, 1H, H-7), 7.25 (td, *J* = 8.0, 4.9 Hz, 1H, H-6), 5.97 (s, 1H, H-3), 2.69 – 2.59 (m, 2H, H-9), 1.64 (p, *J* = 7.4 Hz, 2H, H-10), 1.37 – 1.14 (m, 12H, H-11-16), 0.90 – 0.78 (m, 3H, H-17).

^{13}C NMR (101 MHz, DMSO-d_6) δ 175.9 (C-4), 154.4 (C-2), 151.5 (d, *J* = 249.5 Hz, C-8), 129.4 (d, *J* = 13.1 Hz, C-8a), 126.8 (C-4a), 122.3 (d, *J* = 7.1 Hz, C-6), 120.5 (d, *J* = 3.0 Hz, C-5), 116.1 (d, *J* = 17.2 Hz, C-7), 108.4 (C-3), 32.8 (C-9), 31.2, 28.9, 28.7, 28.7, 28.6, 28.5, 22.1 (C-10-16), 13.9 (C-17).

TOF-HRMS: m/z = 290.1902 $[M+H]^+$, calc. for $C_{18}H_{24}FNO + H^+$ = 290.1914; 312.1718 $[M+Na]^+$, calc. for $C_{18}H_{24}FNO + Na^+$ = 312.1734

Syntheses of 2-nonyl-1,8-naphthyridin-4(1*H*)-one (C8Py-NQ)

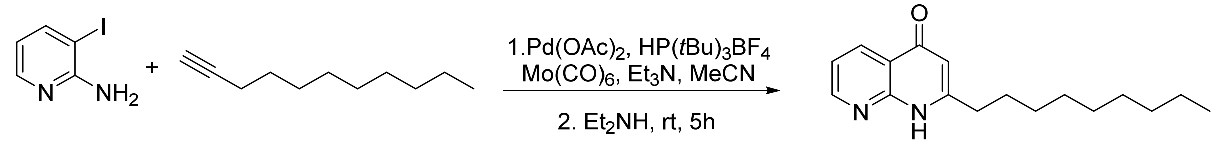

A mixture of 2-amino-1-iodoaniline (220 mg, 1 mmol, 1 eq.), $Pd(OAc)_2$ (4.5 mg, 0.02 mmol, 0.02 eq.), tri-*tert*-butylphosphonium tetrafluoroborate (17.4 mg, 0.06 mmol, 0.06 eq.), and $Mo(CO)_6$ (396 mg, 1.5 mmol, 1.5 eq.) in a sealed vial was evacuated and backfilled with nitrogen gas three times. Acetonitril (4 mL), and 1-undecyne (395 µL, 2 mmol, 2 eq.), and triethylamine (279 µL, 2 mmol, 2 eq.) were added by syringe. The reaction mixture was stirred at room temperature for 16 h whereafter all starting material had been consumed. Diethylamine (517 µL, 5 mmol, 5 eq.) was added to the reaction mixture, and stirred at room temperature for another 5 h. The reaction mixture was poured over water and extracted with chloroform (3 x 15 mL). The combined organic phases were dried over $MgSO_4$, filtered, and concentrated under vacuum. The residue was purified by column chromatography using silica gel and Hexane/EtOAc (gradient from Hexane/EtOAc 1:4) to obtained desired product as brown powder.

Yield 55% R_f = 0.55 (Hexane/EtOAc 1:3)

^{1}H NMR (400 MHz, DMSO-d_6) δ 12.03 (s, 1H, NH), 8.70 (dd, *J* = 4.5, 2.0 Hz, 1H, H-7), 8.40 (dd, *J* = 8.0, 2.1 Hz, 1H, H-5), 7.36 (dd, *J* = 7.9, 4.5 Hz, 1H, H-6), 5.98 (s, 1H, H-3), 2.60 (t, *J* = 7.6 Hz, 2H, H-9), 1.66 (p, *J* = 7.2 Hz, 2H, H-10), 1.37 – 1.13 (m, 12H, H-11-16), 0.89 – 0.76 (m, 3H, H-17).

^{13}C NMR (101 MHz, DMSO-d_6) δ 177.18 (C-4), 155.20 (C-2), 152.64 (C-7), 150.85 (C-8a), 134.38 (C-5), 119.43 (C-4a), 119.01 (C-6), 108.43 (C-3), 32.93 (C-9), 31.22, 28.82, 28.64, 28.62, 28.39, 28.29, 22.05 (C-10-16), 13.90 (C-17).

TOF-HRMS: m/z = 273.1952 $[M+H]^+$, calc. for $C_{17}H_{24}N_2O + H^+$ = 273.1962; 295.1767 $[M+Na]^+$, calc. for $C_{17}H_{24}N_2O + Na^+$ = 295.1781

Synthesis of 6-hydroxy-2-nonylquinolin-4(1*H*)-one (6OH-NQ)

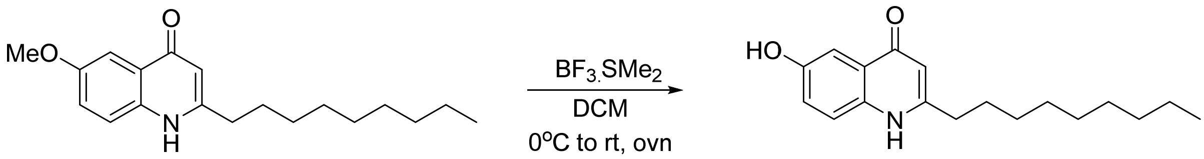

6-Methoxy-2-nonylquinolin-4(1*H*)-one (**6OMe-NQ**) (50 mg, 0.17 mmol, 1 eq.) was dissolved in anhydrous DCM (4 mL) and flushed with nitrogen. After the flask was cooled to 0°C, boron trifluoride-dimethyl sulfite complex (0.523 mL, 4.97 mmol, 30 eq.) was added dropwise. The reaction mixture was allowed to stir at room temperature overnight. The excess $BF_3{\cdot}SMe_2$ was quenched by addition of methanol (15 mL) and left to stir for another 30 min. After solvent was removed under vacuum, the residues were purified by column chromatography using silica gel and DCM/MeOH (gradient from 40:1 to 15:1)

Yield: 98%. R_f = 0.46 (DCM/MeOH 15:1)

^{1}H NMR (400 MHz, DMSO-d_6) δ 11.37 (s, 1H, NH), 9.58 (s, 1H, OH), 7.40 (d, *J* = 8.9 Hz, 1H, H-8), 7.38 – 7.33 (m, 1H, H-5), 7.10 (dd, *J* = 8.8, 2.2 Hz, 1H, H-7), 5.81 (s, 1H H-3), 2.53 (t, *J* = 7.6 Hz, 2H, H-9), 1.69 – 1.57 (m, 2H, H-10), 1.35-1.15 (m, 12H, H-11-16), 0.83 (t, *J* = 6.4 Hz, 3H, H-17).

^{13}C NMR (101 MHz, DMSO-d_6) δ 176.3 (C-4), 153.3 (C-2), 152.2 (C-6), 133.6 (C-8a), 126.0 (C-4a), 121.5 (C-7), 119.3 (C-8), 107.4 (C-5), 106.1 (C-3), 33.2 (C-9), 31.2, 28.9, 28.7, 28.6, 28.5, 28.5, 22.1 (C-10-16), 13.9 (C-17).

TOF-HRMS: m/z = 288.1948 $[M+H]^+$, calc. for $C_{18}H_{25}NO_2 + H^+$ = 288.1958; 310.1765 $[M+Na]^+$, calc. for $C_{18}H_{25}NO_2 + Na^+$ = 310.1778

General synthesis of ethyl carbonates:

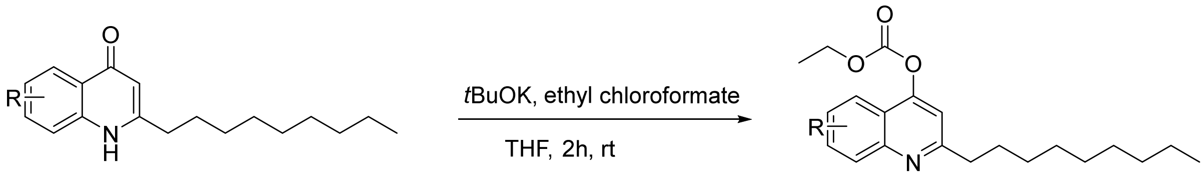

2-Alkyl-4(1*H*)-quinolones (1 eq.) were dissolved in THF (10 mL/ 0.4 g 2-Alkyl-4(1*H*)-quinolones) together with *t*BuOK (1.25 eq.). The reaction mixture was stirred at room temperature for 1 h. Ethyl chloroformate (2.15 eq.) was added and the mixture was stirred at room temperature for another 1 h. The reaction was quenched by the addition of H_2O and the THF was evaporated under reduced pressure. The residue was diluted with H_2O and extracted with ethyl acetate. The combined organic phases were dried with $MgSO_4$, filtered, and evaporated to yield the pure compound. If traces of educt or by-products were visible on TLC, the residue was purified by column chromatography on silica gel using *n*-hexane/ethyl acetate 7:3.

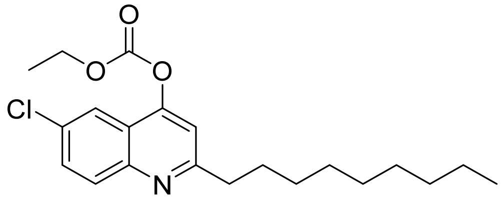

6-Chloro-2-nonylquinolin-4-yl ethyl carbonate: 83%. R_f = 0.80 (Hexane/EtOAc 7:3)

^{1}H NMR (400 MHz, $CDCl_3$-*d*) δ 7.98 (d, *J* = 7.1 Hz, 2H, H-5, H-8), 7.63 (d, *J* = 9.2 Hz, 1H, H-7), 7.35 (s, 1H, H-3), 4.41 (q, *J* = 7.1 Hz, 2H, -OC**H**$_2$CH$_3$), 3.00 – 2.90 (m, 2H, H-9), 1.80 (p, *J* = 7.5 Hz, 2H, H-10), 1.49 – 1.21 (m, 15H, H-11-16, -OCH$_2$C**H**$_3$), 0.87 (t, *J* = 6.5 Hz, 3H, -C**H**$_3$).

^{13}C NMR (101 MHz, $CDCl_3$-*d*) δ 164.6 (C-2), 153.5 (C-4), 152.3 (-O**C**OO), 148.0 (C-8a), 132.2 (C-6), 131.1 (C-7), 130.6 (C-8), 121.3 (C-5), 120.2 (C-4a), 112.6 (C-3), 65.8 (O-**C**H$_2$-CH$_3$), 39.7 (C-9), 32.00, 30.5, 29.8, 29.6 (2C), 29.4, 22.8 (C-10-16), 14.3 (O-CH$_2$-**C**H$_3$), 14.2 (C-17).

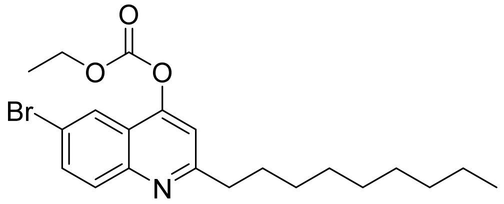

6-Bromo-2-nonylquinolin-4-yl ethyl carbonate: 77%. R_f = 0.77 (Hexane/EtOAc 7:3)

^{1}H NMR (400 MHz, $CDCl_3$-*d*) δ 8.16 (s, 1H, H-5), 7.91 (d, *J* = 9.0 Hz, 1H, H-7), 7.77 (d, *J* = 9.2 Hz, 1H, H-8), 7.35 (s, 1H, H-3), 4.41 (q, *J* = 7.1 Hz, 2H, -OC**H**$_2$CH$_3$), 3.00 – 2.90 (m, 2H, H-9), 1.80 (p, *J* = 7.7 Hz, 2H, H-10), 1.50 – 1.19 (m, 15H, H-11-16,-OCH$_2$C**H**$_3$), 0.87 (t, *J* = 6.5 Hz, 3H, H-17).

^{13}C NMR (101 MHz, $CDCl_3$-*d*) δ 164.8 (C-2), 153.4 (C-4), 152.2 (-O**C**OO), 148.1 (C-8a), 133.7 (C-7), 130.6 (C-8), 123.6 (C-6), 121.7 (C-5), 120.2 (C-4a), 112.6 (C-3), 65.8 (-

OC**H**$_2$CH$_3$), 39.6 (C-9), 32.0, 29.8, 29.60 (2C), 29.59, 29.4, 22.8 (C-10-16), 14.3 (-OCH$_2$C**H**$_3$), 14.2 (C-17).

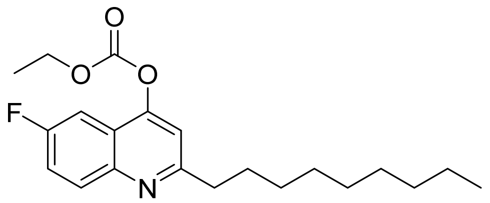

Ethyl (6-fluoro-2-nonylquinolin-4-yl) carbonate: 95%. R$_f$ = 0.72 (Hexane/EtOAc 7:3)

^{1}H NMR (400 MHz, CDCl$_3$-*d)* δ 8.05 (dd, *J* = 9.2, 5.2 Hz, 1H, H-8), 7.60 (dd, *J* = 9.0, 2.4 Hz, 1H, H-5), 7.47 (td, *J* = 9.2, 2.6 Hz, 1H, H-7), 7.35 (s, 1H, H-3), 4.41 (q, *J* = 7.1 Hz, 2H, -OC**H**$_2$CH$_3$), 3.00 – 2.91 (m, 2H, H-9), 1.80 (p, *J* = 7.7 Hz, 2H, H-10), 1.49 – 1.20 (m, 15H, H-11-16, -OCH$_2$C**H**$_3$), 0.87 (t, *J* = 6.6 Hz, 3H, H-17).

^{13}C NMR (101 MHz, CDCl$_3$-*d)* δ 163.5 (C-2), 161.7-159.2 (d, *J* = 248.5 Hz, C-6), 154.0 (C-4), 152.4 (-O**C**OO), 146.8 (C-8a), 131.6 (d, *J* = 9.1 Hz, C-8), 121.3 (d, *J* = 10.1 Hz, C-4a), 120.3 (d, *J* = 25.3 Hz, C-7), 112.6 (C-3), 105.0 (d, *J* = 24.2 Hz, C-5), 65.8 (-O**C**H$_2$CH$_3$), 39.6 (C-9), 34.8, 32.0, 29.9, 29.6 (2C), 29.4, 22.8 (C-10-16), 14.3(-OCH$_2$**C**H$_3$), 14.2 (C-17).

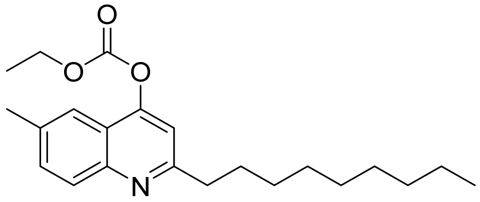

Ethyl (6-methyl-2-nonylquinolin-4-yl) carbonate: 75%. R$_f$ = 0.73 (Hexane/EtOAc 7:3)

^{1}H NMR (400 MHz, CDCl$_3$-*d)* δ 7.98 (d, *J* = 8.5 Hz, 1H, H-8), 7.74 (s, 1H, H-5), 7.54 (d, *J* = 9.7 Hz, 1H, H-7), 7.27 (s, 1H, H-3), 4.45 – 4.36 (m, 2H, -OC**H**$_2$CH$_3$), 3.04 – 2.91 (m, 2H, H-9), 2.53 (s, 3H, Ar-CH$_3$), 1.80 (p, *J* = 7.6 Hz, 2H, H-10), 1.48 – 1.22 (m, 15H, H-11-16, -OCH$_2$C**H**$_3$), 0.87 (t, *J* = 6.8 Hz, 3H, H-17).

^{13}C NMR (101 MHz, CDCl$_3$-*d)* δ 163.1 (C-2), 154.1 (C-4), 152.5 (-O**C**OO), 136.3 (C-6), 132.6 (C-7), 128.5 (C-8), 120.5 (C-6), 119.8 (C-4a), 112.0 (C-3), 65.6 (-O**C**H$_2$CH$_3$), 39.4 (C-9), 32.0, 30.0, 29.65, 29.63 (2C), 29.4, 22.8 (C-10-16), 21.8 (Ar-**C**H$_3$), 14.3 (-OCH$_2$**C**H$_3$), 14.2 (C-17).

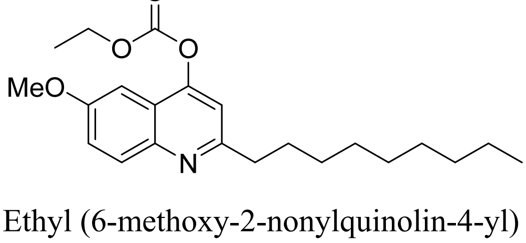

Ethyl (6-methoxy-2-nonylquinolin-4-yl) carbonate: 91%. R$_f$ = 0.65 (Hexane/EtOAc 7:3)

^{1}H NMR (600 MHz, CDCl$_3$-*d)* δ 7.98 (d, *J* = 7.3 Hz, 1H, H-8), 7.36 (dd, *J* = 9.2, 2.5 Hz, 1H, H-7), 7.28 (s, 1H, H-3), 7.20 (d, *J* = 6.0 Hz, 1H, H-5), 4.41 (q, *J* = 7.1 Hz, 2H, -OC**H**$_2$CH$_3$),

3.93 (s, 3H, Ar-O**Me**), 2.94 (t, *J* = 7.8 Hz, 2H, H-9), 1.79 (p, *J* = 7.7 Hz, 2H, H-10), 1.47 – 1.23 (m, 15H, H-11-16, -OCH$_2$C**H**$_3$), 0.87 (t, *J* = 6.9 Hz, 3H, H-17).

^{13}C NMR (151 MHz, CDCl$_3$-*d)* δ 161.3 (C-2), 157.8 (C-4), 153.6 (C-6), 152.5 (-O**C**OO), 130.4 (C-8a), 122.9 (C-8), 121.3 (C-7), 119.0 (C-4a), 112.2 (C-3), 98.9 (C-5), 65.6 (-O**C**H$_2$CH$_3$), 55.8 (Ar-O**Me**), 39.3 (C-9), 32.0, 30.1, 29.6 (2C), 29.4, 22.8 (C-10-16), 14.4 (-OCH$_2$**C**H$_3$), 14.2 (C-17).

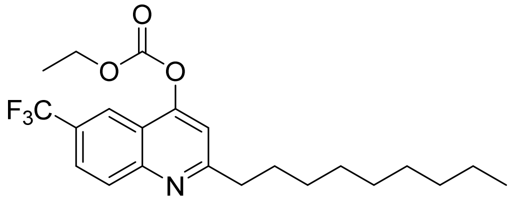

Ethyl (2-nonyl-6-(trifluoromethyl)quinolin-4-yl) carbonate: quant. R$_f$ = 0.82 (Hexane/EtOAc 7:3)

^{1}H NMR (400 MHz, CDCl$_3$-*d)* δ 8.33 (s, 1H, H-5), 8.16 (d, *J* = 8.9 Hz, 1H, H-8), 7.88 (dd, *J* = 8.9, 1.9 Hz, 1H, H-7), 7.45 (s, 1H, H-3), 4.43 (q, *J* = 7.1 Hz, 2H, -OC**H**$_2$CH$_3$), 3.04 – 2.95 (m, 2H, H-9), 1.83 (p, *J* = 7.6 Hz, 2H, H-10), 1.47 (t, *J* = 7.1 Hz, 3H, -OCH$_2$C**H**$_3$), 1.45 – 1.23 (m, 12H, H-11-16), 0.87 (t, *J* = 6.8 Hz, 3H, H-17).

^{13}C NMR (101 MHz, CDCl$_3$-*d)* δ 166.9 (C-2), 154.8 (C-4), 152.2 (-O**C**OO), 150.5 (C-8a), 130.2 (C-8), 129.9 (C-7), 125.93 (Ar-**C**F$_3$), 125.9 (C-6), 119.8 (C-5), 119.5 (C-4a), 112.8 (C-3), 65.9 (-O**C**H$_2$CH$_3$), 39.9 (C-9), 32.0, 30.5, 29.8, 29.6, 29.4, 22.8 (C-10-16), 14.3 (-OCH$_2$**C**H$_3$), 14.2 (C-17).

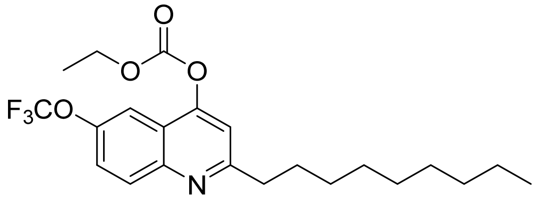

Ethyl (2-nonyl-6-(trifluoromethoxy)quinolin-4-yl) carbonate:quant. R$_f$ = 0.80 (Hexane/EtOAc 7:3)

^{1}H NMR (400 MHz, CDCl$_3$-*d)* δ 8.12 (s, 1H, H-5), 7.83 (s, 1H, H-8), 7.58 (d, *J* = 9.1 Hz, 1H, H-7), 7.42 (s, 1H, H-3), 4.42 (q, *J* = 7.1 Hz, 2H, -OC**H**$_2$CH$_3$), 3.06 – 2.92 (m, 2H, H-9), 1.81 (p, *J* = 7.4 Hz, 2H, H-10), 1.46 (t, *J* = 7.1 Hz, 3H, -OCH$_2$C**H**$_3$), 1.42 – 1.22 (m, 12H, H-11-16), 0.87 (t, *J* = 6.7 Hz, 3H, H-17).

^{13}C NMR (101 MHz, CDCl$_3$-*d)* δ 164.93 (C-6), 154.35 (-O**C**OO), 152.15 (C-2), 147.53 (C-4), 146.93 (C-8a), 131.06 (Ar-O**C**F$_3$), 124.37 (C-8), 120.84 (C-4a), 112.63 (C-3, C-7), 112.17 (C-5), 65.89 (-O**C**H$_2$CH$_3$), 39.51 (C-9), 32.00, 29.87, 29.61, 29.59 (2C), 29.41 (C-10-15), 22.80 (C-16), 14.28 (-OCH$_2$**C**H$_3$), 14.22 (C-17).

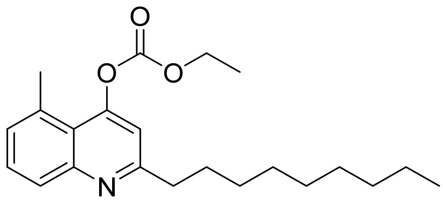

Ethyl (5-methyl-2-nonylquinolin-4-yl) carbonate: 93%. R$_f$ = 0.93 (Hexane/EtOAc 7:3)

^{1}H NMR (400 MHz, CDCl$_3$-*d*) δ 7.92 (d, *J* = 8.5 Hz, 1H, H-8), 7.55 (t, *J* = 7.8 Hz, 1H, H-7), 7.27 (s, 1H, H-6), 7.13 (s, 1H, H-3), 4.40 (q, *J* = 7.1 Hz, 2H, -OC**H**$_2$CH$_3$), 2.99 – 2.91 (m, 2H, H-9), 2.78 (s, 3H, Ar-**Me**), 1.81 (p, *J* = 7.5 Hz, 2H, H-10), 1.48 – 1.20 (m, 15H, H-11-16, -OCH$_2$CH$_3$), 0.88 (t, *J* = 6.5 Hz, 3H, H-17).

^{13}C NMR (101 MHz, CDCl$_3$-*d*) δ 163.5 (C-2), 155.7 (C-4), 153.0 (-O**C**OO), 151.4 (C-8a), 133.2 (C-7), 129.6 (C-5), 129.0 (C-8), 127.7 (C-6), 120.5 (C-4a) 114.0 (C-3), 65.5 (-O**C**H$_2$CH$_3$), 39.2 (C-9), 32.0, 29.8, 29.6 (2C), 29.4 (C-10-15), 23.3 (Ar-**Me**), 22.8 (C-16), 14.4 (-O**C**H$_2$CH$_3$), 14.2 (C-17).

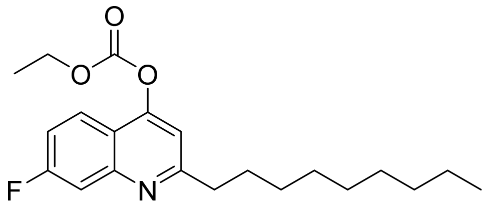

Ethyl (7-fluoro-2-nonylquinolin-4-yl) carbonate: quant. R*f* = 0.78 (Hexane/EtOAc 7:3)

^{1}H NMR (400 MHz, CDCl$_3$-*d*) δ 8.00 (dd, *J* = 9.2, 6.0 Hz, 1H, H-8), 7.69 (dd, *J* = 10.2, 2.2 Hz, 1H, H-5), 7.33 – 7.26 (m, 2H, H-6, H-3), 4.40 (q, *J* = 7.1 Hz, 2H, -OC**H**$_2$CH$_3$), 3.00 – 2.91 (m, 2H, H-9), 1.80 (p, *J* = 7.7 Hz, 2H, H-10), 1.48 – 1.20 (m, 15H, H-11-16, -OCH$_2$C**H**$_3$), 0.92 – 0.82 (m, 3H, H-17).

^{13}C NMR (101 MHz, CDCl$_3$-*d*) δ 165.8 (C-2), 163.2 (d, *J* = 251.5 Hz, C-7), 154.5 (C-4), 152.3 (-O**C**OO), 123.5 (d, *J* = 10.1 Hz, C-8a), 117.6 (d, *J* = 1.1 Hz, C-4a), 116.5 (d, *J* = 25.3 Hz, C-6), 112.8 (d, *J* = 21.2 Hz, C-8), 111.3 (C-3), 65.7 (-O**C**H$_2$CH$_3$), 39.7, 32.0, 29.9, 29.6, 29.4, 22.8, 14.3 (-OCH$_2$**C**H$_3$), 14.2 (C-17).

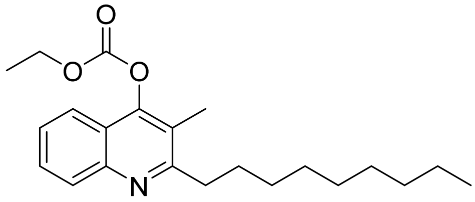

Ethyl (3-methyl-2-nonylquinolin-4-yl) carbonate: 98%. R$_f$ =0.68 (Hexane/EtOAc 7:3)

^{1}H NMR (400 MHz, CDCl$_3$-*d*) δ 8.04 (d, *J* = 8.4 Hz, 1H, H-8), 7.82 – 7.76 (m, 1H, H-5), 7.65 (ddd, *J* = 8.4, 6.9, 1.3 Hz, 1H, H-7), 7.49 (ddd, *J* = 8.1, 7.0, 1.0 Hz, 1H, H-6), 4.38 (q, *J* = 7.1 Hz, 2H, -OC**H**$_2$CH$_3$), 3.03 – 2.95 (m, 2H, H-9), 2.35 (s, 3H, -C**H**$_3$), 1.83 – 1.74 (m, 2H, H-10), 1.51 – 1.21 (m, 15H, H-11-16, -OCH$_2$C**H**$_3$), 0.92 – 0.83 (m, 3H, H-17).

^{13}C NMR (101 MHz, CDCl$_3$-*d*) δ 163.9 (C-2), 152.5 (-O**C**OO), 151.8 (C-4), 147.6 (C-8a), 129.2 (C-7), 128.9 (C-8), 126.4 (C-6), 121.4 (C-5), 121.2 (C-4a), 120.6 (C-3), 65.7(-O**C**H$_2$CH$_3$), 37.1 (C-9), 32.0, 30.0, 29.7 (2C), 29.4, 29.0, 22.8 (C-10-16), 14.4 (-OCH$_2$**C**H$_3$), 14.2 (C-17), 12.1 (-**C**H$_3$).

General synthesis of ethyl carbonate N-oxides

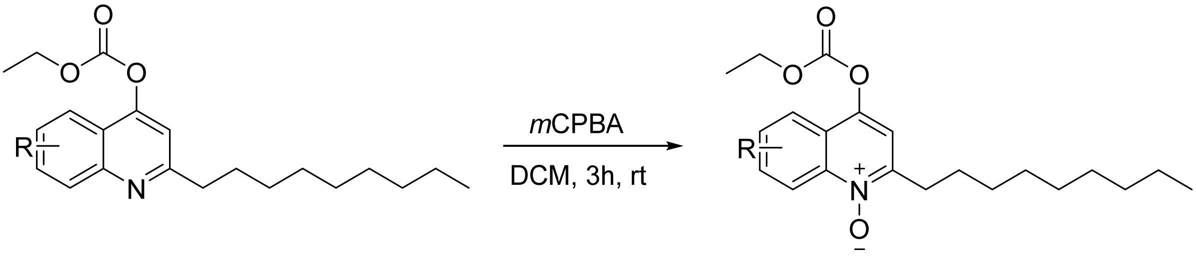

Ethyl carbonates were dissolved in DCM (10 mL/ 250 mg educt) together with *m*CPBA (1.1 eq). The reaction mixture was stirred at room temperature for 3 h. The solution was washed twice with aqueous Na_2CO_3 0.5 M and once with H_2O. The organic phases were dried with $MgSO_4$, filtered, and evaporated. The residue was purified by column chromatography on silica gel using Hexane/EtOAc.

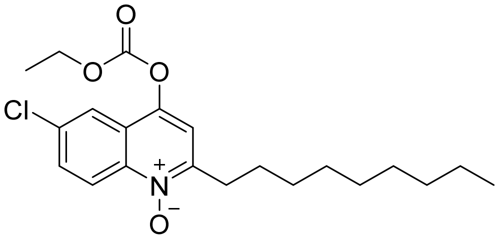

6-Chloro-4-((ethoxycarbonyl)oxy)-2-nonylquinoline 1-oxide: 90%. R_f = 0.80 (EtOAc)

^{1}H NMR (400 MHz, $CDCl_3$-*d*) δ 8.73 (d, *J* = 9.3 Hz, 1H, H-8), 7.99 (d, *J* = 2.1 Hz, 1H, H-5), 7.72 (dd, *J* = 9.3, 2.1 Hz, 1H, H-7), 7.43 (s, 1H, H-3), 4.42 (q, *J* = 7.1 Hz, 2H, -OC**H**$_2$CH$_3$), 3.17 – 3.07 (m, 2H, H-9), 1.80 (p, *J* = 7.6 Hz, 2H, H-10), 1.54 – 1.17 (m, 15H, H-11-16, -OCH$_2$C**H**$_3$), 0.87 (t, *J* = 6.8 Hz, 3H, H-17).

^{13}C NMR (101 MHz, $CDCl_3$-*d*) δ 155.7 (-O**C**OO), 152.2 (C-2), 145.9 (C-4), 140.8 (C-8a), 135.0 (C-6), 132.0 (C-7), 123.5 (C-5), 122.3 (C-8), 121.1 (C-4a), 114.3 (C-3), 66.2 (-O**C**H$_2$CH$_3$), 32.0 (C-9), 31.9, 29.7, 29.6, 29.5, 29.4, 26.2, 22.8 (C-10-16), 14.3 (-OCH$_2$**C**H$_3$), 14.2 (C-17).

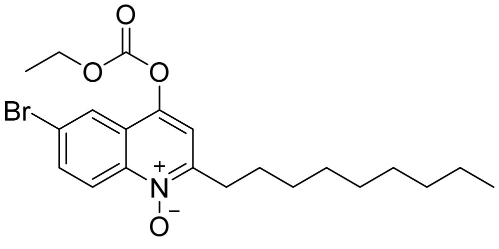

6-Bromo-4-((ethoxycarbonyl)oxy)-2-nonylquinoline 1-oxide: 93%. R_f = 0.82 (EtOAc)

^{1}H NMR (400 MHz, $CDCl_3$-*d*) δ 8.65 (d, *J* = 9.3 Hz, 1H, H-8), 8.17 (d, *J* = 2.0 Hz, 1H, H-5), 7.85 (dd, *J* = 9.3, 2.0 Hz, 1H, H-7), 7.41 (s, 1H, H-3), 4.42 (q, *J* = 7.1 Hz, 2H, -OC**H**$_2$CH$_3$), 3.15 – 3.04 (m, 2H, H-9), 1.80 (p, *J* = 7.6 Hz, 2H, H-10), 1.55 – 1.15 (m, 15H, H-11-16, -OCH$_2$C**H**$_3$), 0.87 (t, *J* = 6.8 Hz, 3H, H-17).

^{13}C NMR (101 MHz, $CDCl_3$-*d*) δ 152.2 (-O**C**OO), 150.4 (C-2), 142.9 (C-4), 141.1 (C-8a), 134.5 (C-7), 124.4 (C-5), 123.8 (C-8), 123.0 (C-6), 122.3 (C-4a), 114.3 (C-3), 66.1 (-

O**C**H$_2$CH$_3$), 32.0 (C-9), 31.9, 29.7, 29.6, 29.5, 29.4, 26.1, 22.8 (C-10-16), 14.3 (-OCH$_2$**C**H$_3$), 14.2 (C-17).

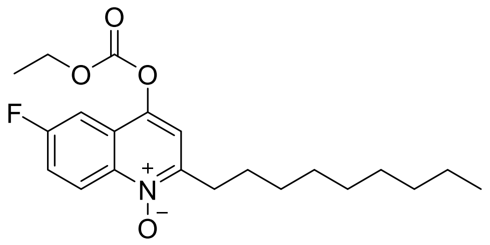

4-((Ethoxycarbonyl)oxy)-6-fluoro-2-nonylquinoline 1-oxide: 82%. R$_f$ = 0.68 (Hexane/EtOAc 1:3)

^{1}H NMR (400 MHz, CDCl$_3$-*d*) δ 8.78 (dd, *J* = 9.6, 5.1 Hz, 1H, H-8), 7.58 (dd, *J* = 8.7, 2.6 Hz, 1H, H-5), 7.53 – 7.45 (m, 1H, H-7), 7.38 (s, 1H, H-3), 4.38 (q, *J* = 7.1 Hz, 2H, -OC**H**$_2$CH$_3$), 3.12 – 3.01 (m, 2H, H-9), 1.78 (p, *J* = 7.6 Hz, 2H, H-10), 1.50 – 1.14 (m, 15H, H-11-16, -OCH$_2$C**H**$_3$), 0.90 – 0.77 (m, 3H, H-17).

^{13}C NMR (101 MHz, CDCl$_3$-*d*) δ 161.3 (d, *J* = 252.5 Hz, C-6), 152.3 (-O**C**OO), 149.1 (C-2), 143.1 (d, *J* = 6.1 Hz, C-4), 139.4 (C-8a), 124.0 (d, *J* = 10.1 Hz, C-8), 123.5 (d, *J* = 9.1 Hz, C-4a), 120.8 (d, *J* = 25.3 Hz, C-7), 114.4 (C-3), 106.4-106.2 (d, *J* = 24.2 Hz, C-7), 66.0 (-O**C**H$_2$CH$_3$), 31.9 (C-9), 31.7, 29.6, 29.6, 29.5, 29.4, 26.1, 22.7 (C-10-16), 14.24 (-OCH$_2$**C**H$_3$), 14.17 (C-17).

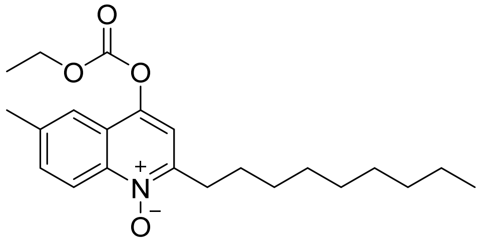

4-((Ethoxycarbonyl)oxy)-6-methyl-2-nonylquinoline 1-oxide: 69%. R$_f$ = 0.62 (EtOAc)

^{1}H NMR (400 MHz, CDCl$_3$-*d*) δ 8.66 (d, *J* = 8.9 Hz, 1H, H-8), 7.75 (s, 1H, H-5), 7.62 (d, *J* = 9.0 Hz, 1H, H-6), 7.33 (s, 1H, H-3), 4.41 (q, *J* = 7.1 Hz, 2H, -OC**H**$_2$CH$_3$), 3.12 (t, *J* = 7.7 Hz, 2H, H-9), 2.55 (s, 3H, Ar-C**H**$_3$), 1.81 (p, *J* = 7.6 Hz, 2H, H-10), 1.51 – 1.18 (m, 15H, H-11-16, -OCH$_2$C**H**$_3$), 0.87 (t, *J* = 6.7 Hz, 3H, H-17).

^{13}C NMR (101 MHz, CDCl$_3$-*d*) δ 154.6 (-O**C**OO), 152.5 (C-2), 149.4 (C-4), 141.0 (C-8a), 138.7 (C-6), 133.5 (C-7), 122.7 (C-5), 120.8 (C-8), 120.1 (C-4a), 113.3 (C-3), 65.9 (-OC**H**$_2$CH$_3$), 32.0 (C-9), 31.8, 29.7, 29.2, 29.5, 29.4, 26.3, 22.8 (C-10-16), 21.7 (Ar-C**H**$_3$), 14.3 (-OCH$_2$C**H**$_3$), 14.2 (C-17).

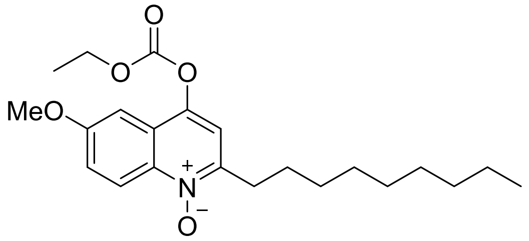

4-((Ethoxycarbonyl)oxy)-6-methoxy-2-nonylquinoline 1-oxide: 77%. R$_f$ = 0.62 (EtOAc)

^{1}H NMR (400 MHz, $CDCl_3$-*d*) δ 8.69 (d, *J* = 9.6 Hz, 1H, H-8), 7.41 (dd, *J* = 9.5, 2.2 Hz, 1H, H-7), 7.35 (s, 1H, H-3), 7.20 (d, *J* = 2.2 Hz, 1H, H-5), 4.42 (q, *J* = 7.1 Hz, 2H, -OC**H**$_2$CH$_3$), 3.95 (s, 3H, Ar-O**Me**), 3.17 – 3.06 (m, 2H, H-9), 1.80 (p, *J* = 7.6 Hz, 2H, H-10), 1.51 – 1.19 (m, 15H, H-11-16, -OCH$_2$C**H**$_3$), 0.87 (t, *J* = 6.6 Hz, 3H, H-17).

^{13}C NMR (101 MHz, $CDCl_3$-*d*) δ 159.4 (C-6), 152.4 (-O**C**OO), 152.17 (C-2), 152.15 (C-4), 138.2 (C-8a), 137.8(C-8), 123.9 (C-7), 122.1 (C-4a), 113.8 (C-3), 100.1 (C-5), 66.0 (-O**C**H$_2$CH$_3$), 56.0 (Ar-O**Me**), 32.0 (C-9), 31.7, 29.1, 29.6, 29.5, 29.4, 26.4, 22.8 (C-10-16), 14.3 (-OCH$_2$**C**H$_3$), 14.2 (C-17).

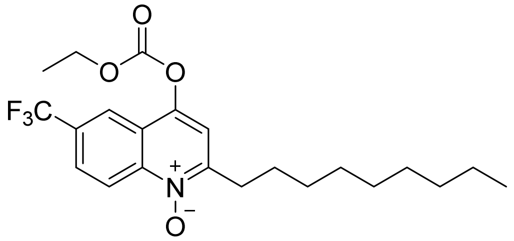

4-((Ethoxycarbonyl)oxy)-2-nonyl-6-(trifluoromethyl)quinoline 1-oxide: 72%. R*f* = 0.79 (Hexane/EtOAc 1:2)

^{1}H NMR (400 MHz, $CDCl_3$-*d*) δ 8.92 (d, *J* = 9.1 Hz, 1H, H-8), 8.33 (s, 1H, H-5), 7.96 (dd, *J* = 9.2, 1.8 Hz, 1H, H-7), 7.51 (s, 1H, H-3), 4.44 (q, *J* = 7.1 Hz, 2H, -OC**H**$_2$CH$_3$), 3.19 – 3.09 (m, 2H, H-9), 1.82 (p, *J* = 7.6 Hz, 2H, H-10), 1.52 – 1.20 (m, 15H, H-11-16, -OCH$_2$C**H**$_3$), 0.93 – 0.81 (m, 3H, H-17).

^{13}C NMR (101 MHz, $CDCl_3$-*d*) δ 152.2 (-O**C**OO), 151.9 (C-2), 143.8 (C-4), 143.3 (C-8a), 130.6 (C-6), 126.8 (C-5), 122.2 (C-7), 121.9 (Ar-**C**F$_3$), 120.4 (C-8), 120.3 (C-4a), 114.5 (C-3), 66.2 (-O**C**H$_2$CH$_3$), 32.01 (C-9), 31.99, 29.7, 29.6, 29.5, 29.4, 26.0, 22.8 (C-10-16), 14.3 (-OCH$_2$**C**H$_3$), 14.2 (C-17).

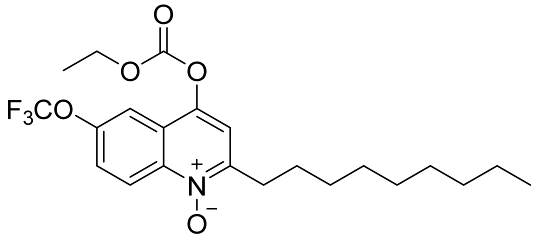

4-((Ethoxycarbonyl)oxy)-2-nonyl-6-(trifluoromethoxy)quinoline 1-oxide: 62%. R*f* = 0.82 (EtOAc)

^{1}H NMR (400 MHz, $CDCl_3$-*d*) δ 8.12 (d, *J* = 8.9 Hz, 1H, H-8), 7.82 (s, 1H, H-5), 7.62 – 7.52 (m, 1H, H-7), 7.42 (s, 1H, H-3), 4.42 (q, *J* = 7.1 Hz, 2H, -OC**H**$_2$CH$_3$), 3.07 – 2.91 (m, 2H, H-9), 1.81 (p, *J* = 7.6 Hz, 2H, H-10), 1.48 – 1.20 (m, 15H, H-11-16, -OCH$_2$C**H**$_3$), 0.87 (t, *J* = 6.7 Hz, 3H, H-17).

^{13}C NMR (101 MHz, $CDCl_3$-*d*) δ 164.9 (C-6), 154.4 (-O**C**OO), 152.2 (C-2), 147.5 (C-4), 146.9 (C-8a), 131.1 (Ar-O**C**F$_3$), 124.4 (C-8), 120.8 (C-4a), 112.6 (C-3, C-7), 112.2 (C-5), 65.9 (-O**C**H$_2$CH$_3$), 39.5 (C-9), 32.0, 29.9, 29.61, 29.59 (2C), 29.4, 22.8 (C-10-16), 14.3 (-OCH$_2$**C**H$_3$), 14.2 (C-17).

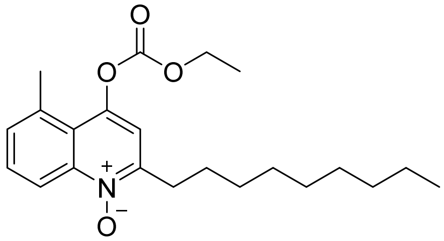

4-((Ethoxycarbonyl)oxy)-5-methyl-2-nonylquinoline 1-oxide: 88%. R_f = 0.62 (Hexane/EtOAc 1:2)

^{1}H NMR (800 MHz, $CDCl_3$-*d*) δ 8.74 (d, *J* = 8.8 Hz, 1H, H-8), 7.66 – 7.60 (m, 1H, H-7), 7.38 (d, *J* = 7.1 Hz, 1H, H-6), 7.14 (s, 1H, H-3), 4.40 (q, *J* = 7.1 Hz, 2H,-OC**H**$_2$CH$_3$), 3.12 – 3.08 (m, 2H, H-9), 2.78 (s, 3H, Ar-**Me**), 1.80 (p, *J* = 7.8 Hz, 2H, H-10), 1.49-1.41 (m, 5H, H-11, -OCH$_2$C**H**$_3$), 1.37 (p, *J* = 7.0 Hz, 2H, H-12), 1.32-1.22 (m, 8H, H-13-16), 0.87 (t, *J* = 7.1 Hz, 3H, H-17).

^{13}C NMR (201 MHz, $CDCl_3$-*d*) δ 153.0 (-O**C**OO), 149.2 (C-2), 145.0 (C-4), 144.2 (C-8a), 134.4 (C-5), 131.2 (C-6), 130.6 (C-7), 122.5 (C-8), 118.8 (C-4a), 115.1 (C-3), 65.8 (-OC**H**$_2$CH$_3$), 32.0 (C-9), 31.6, 29.7, 29.6, 29.55, 29.4, 26.0 (C-10-15), 23.0 (Ar-**Me**), 22.8 (C-16), 14.4 (-OCH$_2$C**H**$_3$), 14.2 (C-17).

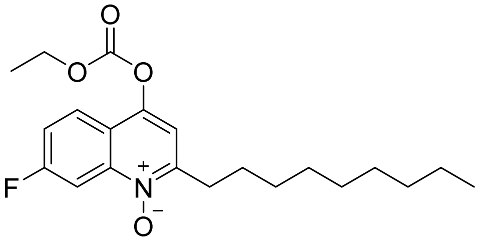

4-((Ethoxycarbonyl)oxy)-7-fluoro-2-nonylquinoline 1-oxide: 68%. R_f = 0.87 (Hexane/EtOAc 1:3)

^{1}H NMR (400 MHz, $CDCl_3$-*d*) δ 8.46 (dd, *J* = 10.1, 2.5 Hz, 1H, H-5), 8.04 (dd, *J* = 9.2, 5.4 Hz, 1H, H-8), 7.41 (ddd, *J* = 9.2, 7.7, 2.6 Hz, 1H, H-6), 7.36 (s, 1H, H-3), 4.41 (q, *J* = 7.1 Hz, 2H, -OC**H**$_2$CH$_3$), 3.17 – 3.08 (m, 2H, H-9), 1.81 (p, *J* = 7.6 Hz, 2H, H-10), 1.51 – 1.19 (m, 15H, H-11-16, -OCH$_2$C**H**$_3$), 0.91 – 0.83 (m, 3H, H-17).

^{13}C NMR (101 MHz, $CDCl_3$-*d*) δ 164.5 (d, *J* = 254.5 Hz, C-7), 152.3 (-O**C**OO), 151.3 (C-2), 144.1 (C-4), 143.5 (d, *J* = 11.1 Hz, C-8a), 124.9 (d, *J* = 9.1 Hz, C-5), 119.7 (C-4a), 118.6 (d *J* = 26.3 Hz, H-6), 112.7 (C-3), 105.7 (d, *J* = 27.3 Hz, C-8), 66.1 (-O**C**H$_2$CH$_3$), 32.0 (C-9), 31.1, 29.7, 29.6, 29.5, 29.4, 26.1, 22.8 (C-10-16), 14.3 (-OCH$_2$**C**H$_3$), 14.2 (C-17).

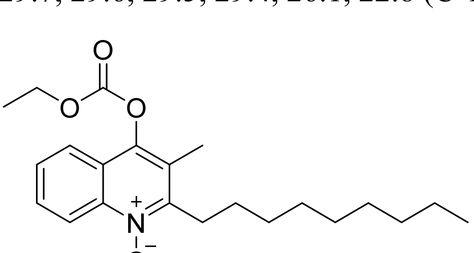

4-((Ethoxycarbonyl)oxy)-3-methyl-2-nonylquinoline 1-oxide: 75%. R_f = 0.68 (Hexane/EtOAc 1:4)

^1H NMR (400 MHz, $CDCl_3$-*d*) δ 8.75 (d, *J* = 8.7 Hz, 1H, H-8), 7.81 (d, *J* = 8.3 Hz, 1H, H-5), 7.72 (ddd, *J* = 8.5, 7.0, 1.2 Hz, 1H, H-7), 7.64 – 7.57 (m, 1H, H-6), 4.39 (q, *J* = 7.1 Hz, 2H, -OC**H**$_2$CH$_3$), 3.23 – 3.15 (m, 2H, H-9), 2.35 (s, 3H, -C**H**$_3$), 1.73 (p, *J* = 7.6 Hz, 2H, H-10), 1.55 – 1.19 (m, 15H, H-11-16, -OCH$_2$C**H**$_3$), 0.91 – 0.82 (m, 3H, H-17).

^{13}C NMR (101 MHz, $CDCl_3$-*d*) δ 152.6 (-O**C**OO), 151.0 (C-2), 142.2 (C-4), 140.9 (C-8a), 130.2 (C-7), 128.5 (C-6), 123.1 (C-4a), 123.0 (C-5), 121.3 (C-8), 120.4 (C-3), 66.0 (-O**C**H$_2$CH$_3$), 32.0 (C-9), 30.3, 29.7, 29.5, 29.4, 29.1, 25.5, 22.8 (C-10-16), 14.3 (-OCH$_2$**C**H$_3$), 14.2 (C-17), 12.8 (-**C**H$_3$).

General synthesis of 1-hydroxy-quinoline-4-ones

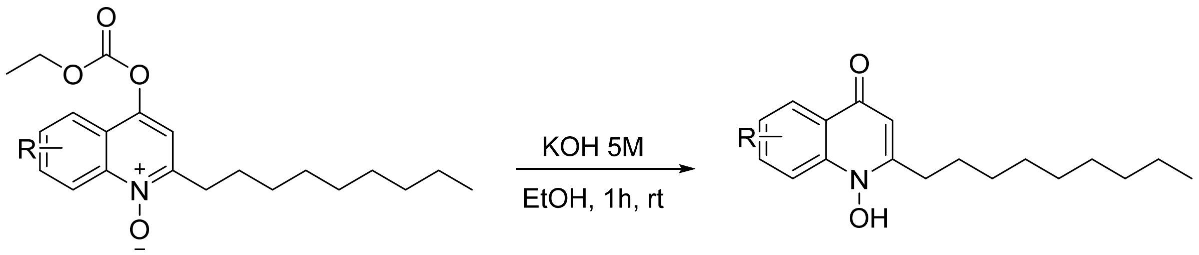

Ethyl-carbonate protected 4(1*H*)-quinolone *N*-oxides were dissolved in EtOH (10 mL/ 300 mg educts and aqueous KOH 5M (20 eq.) was added dropwise. The resulting yellow coloured reaction mixture was stirred at room temperature for 1 h. H_2O was added and the reaction was cooled to 0°C, and pH was adjusted to 1-2 with conc. HCl to form the milky suspension which soon crystallized. The product was collected by vacuum filtration and washed with cold H_2O. Recrystallization with EtOH/H_2O allowed to obtain the desired product as crystalline white solid.

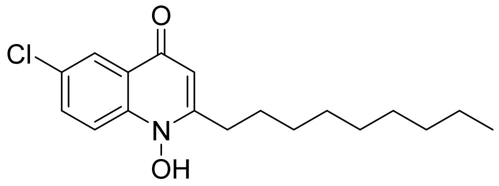

6-Chloro-1-hydroxy-2-nonylquinolin-4(1*H*)-one (**6Cl-NQNO**): 46%

^{1}H NMR (400 MHz, $CDCl_3$-*d*) δ 8.15 (d, *J* = 2.4 Hz, 1H, H-5), 8.08 (d, *J* = 9.2 Hz, 1H, H-7), 7.57 (dd, *J* = 9.1, 2.4 Hz, 1H, H-8), 6.12 (s, 1H, H-3), 2.63 (t, *J* = 8.0 Hz, 2H, H-9), 1.43 (q, *J* = 7.2 Hz, 2H, H-10), 1.28 – 1.15 (m, 12H, H-11-16), 0.86 (t, *J* = 7.0 Hz, 3H, H-17).

^{13}C NMR (101 MHz, $CDCl_3$-*d*) δ 170.19 (C-4), 158.16 (C-2), 138.93 (C-8a), 133.50 (C-6), 123.89 (C-7), 118.79 (C-4a), 106.00 (C-5), 93.42 (C-8), 85.80 (C-3), 32.03 (C-9), 29.86, 29.68, 29.62, 29.47, 29.43, 27.58, 22.81 (C-10-16), 14.23 (C-17).

TOF-HRMS: m/z = 322.1564 $[M+H]^+$, calc. for $C_{18}H_{24}ClNO_2$ + H^+ = 322.1569; 344.1389 $[M+Na]^+$, calc. for $C_{18}H_{24}ClNO_2$ + Na^+ = 344.1388

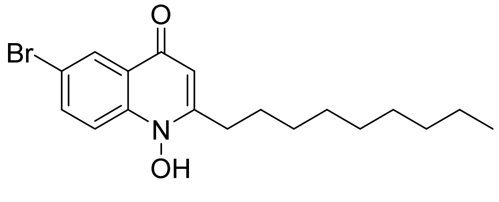

6-Bromo-1-hydroxy-2-nonylquinolin-4(1*H*)-one (**6Br-NQNO)**: 33%

^{1}H NMR (400 MHz, $CDCl_3$-*d*) δ 8.31 (d, *J* = 1.8 Hz, 1H, H-5), 8.01 (d, *J* = 9.2 Hz, 1H, H-7), 7.71 (dd, *J* = 9.1, 2.4 Hz, 1H, H-8), 6.08 (s, 1H, H-3), 2.61 (t, *J* = 8.0 Hz, 2H, H-9), 1.52 – 1.35 (m, 2H, H-10), 1.29 – 1.16 (m, 12H, H-11-16), 0.87 (t, *J* = 7.0 Hz, 3H, H-17).

^{13}C NMR (101 MHz, $CDCl_3$-*d*) δ 177.75 (C-4), 139.29 (C-2), 136.00 (C-8a), 133.46 (C-7), 127.32 (C-4a), 125.43 (C-5), 118.74 (C-8), 113.01 (C-6), 106.10 (C-3), 32.05 (C-9), 29.86, 29.70, 29.62, 29.47, 29.45, 27.57, 22.83 (C-10-16), 14.26 (C-17).

TOF-HRMS: m/z = 366.1065 [M+H] $^+$, calc. for $C_{18}H_{24}BrNO_2$ + H^+ = 366.1063; 388.0881 [M+Na] $^+$, calc. for $C_{18}H_{24}BrNO_2$ + Na^+ = 388.0882

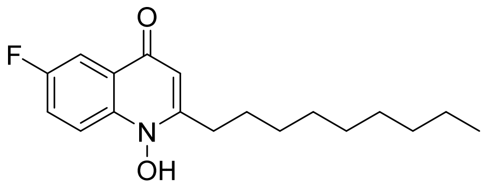

6-Fluoro-1-hydroxy-2-nonylquinolin-4(1*H*)-one (**6F-NQNO**): 62%

^{1}H NMR (400 MHz, $CDCl_3$-*d*) δ 8.19 – 8.04 (m, 1H, H-5), 8.02 – 7.78 (m, 1H, H-7), 7.54 – 7.31 (m, 1H, H-8), 6.30 – 6.03 (m, 1H, H-3), 2.59 (t, *J* = 7.9 Hz, 2H, H-9), 1.52-1.37 (m, 2H, H-10), 1.27 – 1.13 (m, 12H, H-11-16), 0.86 (t, *J* = 7.0 Hz, 3H, H-17).

^{13}C NMR (101 MHz, $CDCl_3$-*d*) δ 160.1 (C-6, *J* = 248.5 Hz), 155.5 (C-4), 137.5 (C-2), 134.5 (C-8a), 121.6 (C-4a, *J* = 25.3 Hz), 119.6 (C-7, *J* = 7.1 Hz), 116.8 (C-8), 109.2 (C-5, *J* = 23.2 Hz), 105.5 (C-3), 32.0 (C-9), 31.6, 29.9, 29.7, 29.6, 29.4, 27.5, 22.8 (C-10-16), 14.2 (C-17).

TOF-HRMS: m/z = 306.1852 [M+H]$^+$, calc. for $C_{18}H_{24}FNO_2$ + H^+ = 306.1852; 328.1683 [M+Na]$^+$, calc. for $C_{18}H_{24}FNO_2$ + Na^+ = 328.1677.

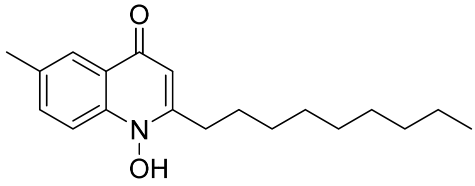

1-Hydroxy-6-methyl-2-nonylquinolin-4(1*H*)-one (**6Me-NQNO)**: 88.50%

^{1}H NMR (400 MHz, $CDCl_3$-*d*) δ 8.04 (d, *J* = 8.8 Hz, 1H, H-5), 7.99 (s, 1H, H-7), 7.44 (d, *J* = 8.9 Hz, 1H, H-8), 6.31 (s, 1H, H-3), 2.62 (t, *J* = 7.8 Hz, 2H, H-9), 2.42 (s, 3H, -C**H**$_3$), 1.53 – 1.39 (m, 2H, H-10), 1.32 – 1.10 (m, 12H, H-11-16), 0.85 (t, *J* = 7.0 Hz, 3H, H-17).

^{13}C NMR (101 MHz, $CDCl_3$-*d*) δ 154.80 (C-2), 138.68 (C-8a), 135.30 (C-6), 134.06 (C-7), 123.40 (C-4a), 122.95 (C-5), 116.49 (C-8), 104.90 (C-3), 31.71 (C-9), 31.33, 29.38, 29.32, 29.13 (2C), 27.22, 22.49 (C-10-16), 20.98 (-**C**H$_3$), 13.92 (C-17).

TOF-HRMS: m/z = 302.2108 [M+H]$^+$, calc. for $C_{19}H_{27}NO_2$ + H^+ = 302.2115; 324.1932 [M+Na]$^+$, calc. for $C_{19}H_{27}NO_2$ + Na^+ = 324.1934

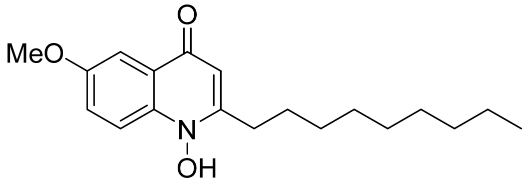

1-Hydroxy-6-methoxy-2-nonylquinolin-4(1*H*)-one (**6OMe-NQNO**): 41%

^{1}H NMR (400 MHz, $CDCl_3$-*d*) δ 8.14 (d, *J* = 9.4 Hz, 1H, H-5), 7.58 (d, *J* = 2.9 Hz, 1H, H-8), 7.28 (dd, *J* = 9.4, 2.8 Hz, 1H, H-7), 6.19 (s, 1H, H-3), 3.87 (s, 3H, -O**Me**), 2.60 – 2.29 (m, 2H, H-9), 1.36 (q, *J* = 6.6 Hz, 2H, H-10), 1.27 – 1.07 (m, 12H, H-11-16), 0.86 (t, *J* = 7.0 Hz, 3H, H-17).

^{13}C NMR (101 MHz, $CDCl_3$-*d*) δ 157.62 (C-2), 153.39 (C-6), 136.08 (C-8a), 124.74 (C-4a), 124.01 (C-8), 118.96 (C-7), 105.05 (C-5), 103.14 (C-3), 55.83 (-OMe), 32.05 (C-9), 31.31, 29.85, 29.72, 29.63, 29.41, 27.49, 22.80 (C-10-16), 14.22 (C-17).

TOF-HRMS: m/z = 318.2057 $[M+H]^+$, calc. for $C_{19}H_{27}NO_3 + H^+$ = 318.2064; 340.1881 $[M+Na]^+$, calc. for $C_{19}H_{27}NO_3 + Na^+$ = 340.1883

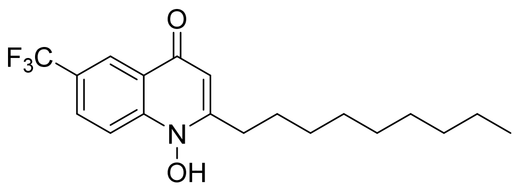

1-Hydroxy-2-nonyl-6-(trifluoromethyl)quinolin-4(1*H*)-one (**6CF$_3$-NQNO)**: 34%

^1H NMR (400 MHz, MeOD-*d*$_4$) δ 8.58 (s, 1H, H-5), 8.20 (d, *J* = 8.8 Hz, 1H, H-8), 8.06 (d, *J* = 9.0 Hz, 1H, H-7), 6.34 (s, 1H, H-3), 2.94 (t, *J* = 7.5 Hz, 2H, H-9), 1.81 (q, *J* = 6.8 Hz, 2H, H-10), 1.56 – 1.24 (m, 12H, H-11-16), 0.91 (t, *J* = 6.4 Hz, 3H, H-17).

^{13}C NMR (101 MHz, MeOD-*d*$_4$) δ 173.9 (C-4, only in HMBC), 158.2 (C-2), 143.6 (C-8a), 129.7 (C-4a), 129.6 (C-7), 125.3 (C-6), 124.20 (Ar-**C**F$_3$), 124.16 (C-5), 117.8 (C-8), 109.2 (C-3), 33.0 (C-9), 32.6, 30.6, 30.4 (2C), 28.9, 23.7 (C-10-16), 14.4 (C-17).

TOF-HRMS: m/z = 356.1826 $[M+H]^+$, calc. for $C_{19}H_{24}F_3NO_2 + H^+$ = 356.1832; 378.1648 $[M+Na]^+$, calc. for $C_{19}H_{24}F_3NO_2 + Na^+$ = 378.1651

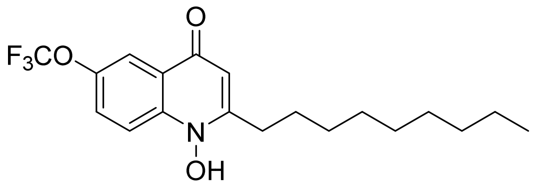

1-Hydroxy-2-nonyl-6-(trifluoromethoxy)quinolin-4(1*H*)-one (**6OCF$_3$-NQNO**): 41%

^1H NMR (400 MHz, $CDCl_3$-*d)* δ 8.18 (d, *J* = 9.4 Hz, 1H, H-5), 8.05 (d, *J* = 2.7 Hz, 1H, H-8), 7.49 (dd, *J* = 9.3, 2.9 Hz, 1H, H-7), 6.23 (s, 1H, H-3), 2.69 (t, *J* = 8.2 Hz, 2H, H-9), 1.62 – 1.45 (m, 2H, H-10), 1.28 – 1.18 (m, 12H, H-11-16), 0.85 (t, *J* = 7.0 Hz, 3H, H-17).

^{13}C NMR (101 MHz, $CDCl_3$-*d)* δ 156.62 (C-2), 146.50 (C-6), 138.87 (C-8a), 126.11 (C-4a), 124.47 (-OCF$_3$), 121.86 (C-8), 119.19 (C-7), 115.85 (C-5), 106.08 (C-3), 31.97 (C-9), 31.75, 29.60, 29.55, 29.41, 29.37, 27.61, 22.76 (C-10-16), 14.14 (C-17).

TOF-HRMS: m/z = 372.1774 $[M+H]^+$, calc. for $C_{19}H_{24}F_3NO_3 + H^+$ = 372.1780; 394.1595 $[M+Na]^+$, calc. for $C_{19}H_{24}F_3NO_3 + Na^+$ = 394.1600

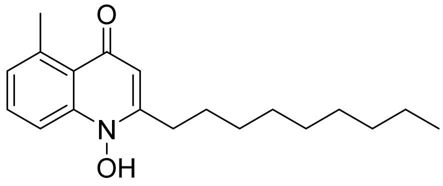

1-Hydroxy-5-methyl-2-nonylquinolin-4(1*H*)-one (**5Me-NQNO)**: 44%

^{1}H NMR (400 MHz, $CDCl_3$-*d*) δ 8.03 (d, *J* = 8.7 Hz, 1H, H-7), 7.43 (t, *J* = 7.9 Hz, 1H, H-8), 7.07 (d, *J* = 7.3 Hz, 1H, H-6), 6.09 (s, 1H, H-3), 2.85 (s, 3H, Ar-Me), 2.48 (t, *J* = 7.9 Hz, 2H, H-9), 1.46 – 1.00 (m, 14H, H-10-16), 0.87 (t, *J* = 7.0 Hz, 3H, H-17).

^{13}C NMR (101 MHz, $CDCl_3$-*d*) δ 169.8 (C-4), 154.5 (C-2), 142.5 (C-8a), 139.6 (C-5), 131.8 (C-7), 127.9 (C-6), 122.4 (C-4a), 114.9 (C-8), 106.3 (C-3), 32.0 (C-9), 31.1, 29.7, 29.6, 29.4, 29.4, 27.2 (C-10-15), 24.0 (Ar-Me), 22.8 (C-16), 14.2 (C-17)

TOF-HRMS: m/z = 302.2106 $[M+H]^+$, calc. for $C_{19}H_{27}NO_2 + H^+$ = 302.2115; 324.1931 $[M+Na]^+$, calc. for $C_{19}H_{27}NO_2 + Na^+$ = 324.1934

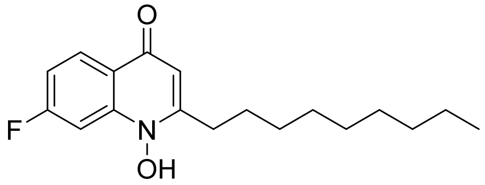

7-Fluoro-1-hydroxy-2-nonylquinolin-4(1*H*)-one (**7F-NQNO**): 66%

^{1}H NMR (400 MHz, $CDCl_3$-*d*) δ 8.19 (dd, *J* = 9.0, 5.9 Hz, 1H, H-5), 7.73 (dd, *J* = 10.1, 2.6 Hz, 1H, H-8), 7.07 (td, *J* = 8.0, 2.5 Hz, 1H, H-6), 6.16 (s, 1H, H-3), 2.66 (t, *J* = 7.9 Hz, 2H, H-9), 1.55-1.44 (m, 2H, H-10), 1.26 – 1.18 (m, 12H, H-11-16), 0.86 (t, *J* = 7.0 Hz, 3H, H-17).

^{13}C NMR (101 MHz, $CDCl_3$-*d*) δ 166.68 (C-4), 164.16 (C-7, *J* = 252.5 Hz), 156.98 (C-2), 142.20, 142.08 (C8a, *J* = 12.1 Hz) 128.08, 127.99 (C-5, *J* = 9.1 Hz), 120.42 (C-4a), 114.72, 114.47 (C-6, *J* = 25.3 Hz), 105.58 (C-3), 102.50, 102.23 (C-8, *J* = 27.3 Hz), 32.01 (C-9), 31.74 (C-10), 29.86, 29.64, 29.58, 29.43, 27.50, 22.81 (C-11-16), 14.22 (C-17).

TOF-HRMS: m/z = 306.1855 $[M+H]^+$, calc. for $C_{18}H_{24}FNO_2 + H^+$ = 306.1864; 328.1680 $[M+Na]^+$, calc. for $C_{18}H_{24}FNO_2 + Na^+$ = 328.1683

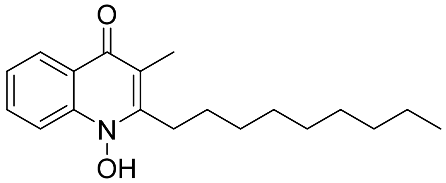

1-Hydroxy-3-methyl-2-nonylquinolin-4(1*H*)-one (**3Me-NQNO)**: 72%

^{1}H NMR (400 MHz, MeOD-d_4) δ 8.31 (dd, *J* = 8.2, 1.0 Hz, 1H, H-5), 7.99 (d, *J* = 8.5 Hz, 1H, H-8), 7.77 (ddd, *J* = 8.6, 7.0, 1.5 Hz, 1H, H-7), 7.45 (ddd, *J* = 8.1, 7.0, 1.0 Hz, 1H, H-6), 3.06 (dd, *J* = 9.3, 6.9 Hz, 2H, H-9), 2.25 (s, 3H, -C**H**$_3$), 1.80 – 1.70 (m, 2H, H-10), 1.58 – 1.26 (m, 12H, H-11-16), 0.97 – 0.85 (m, 3H, H-17).

^{13}C NMR (101 MHz, MeOD-d_4) δ 173.9 (C-4, only in HMBC), 154.2 (C-2), 140.9 (C-8a), 133.1 (C-7), 126.2 (C-6), 125.1 (C-5), 124.5 (C-4a), 115.9 (C-8), 115.8 (C-3), 33.0 (C-9), 30.8, 30.6, 30.40, 30.38, 29.8, 28.8, 23.7 (C-10-16), 14.4 (C-17), 11.6 (-**C**H$_3$).

TOF-HRMS: m/z = 302.2105 $[M+H]^+$, calc. for $C_{19}H_{27}NO_2 + H^+$ = 302.2115; 324.1928 $[M+Na]^+$, calc. for $C_{19}H_{27}NO_2 + Na^+$ = 324.1934

Synthesis of 1,6-dihydroxy-2-nonylquinolin-4(1*H*)-one (6OH-NQNO)

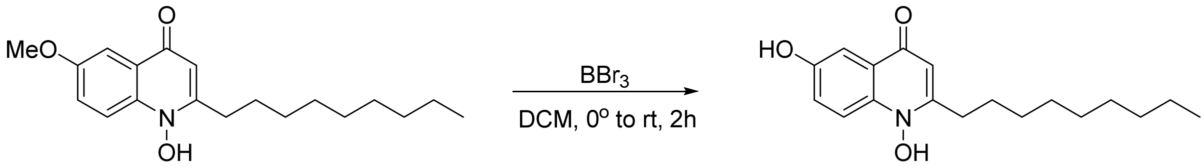

1-Hydroxy-6-methoxy-2-nonylquinolin-4(1*H*)-one (**6OMe-NQNO)** (140 mg, 0.44 mmol, 1 eq.) was dissolved in anhydrous DCM (4.4 mL) and flushed with nitrogen. After the flask was cooled to 0°C, boron tribromide (1M in DCM) (1.32 mL, 1.32 mmol, 3 eq.) was added dropwise. The reaction mixture was warmed to room temperature and stirred for 2 h. H_2O was added and the reaction was cooled to 0°C, and pH was adjusted to 1-2 with conc. HCl to form a milky suspension which soon crystallized. The product was collected by vacuum filtration and washed with cold H_2O. Recrystallization with EtOH/H_2O to obtain the desired product as crystalline white solid.

Yield: 95%

^{1}H NMR (400 MHz, MeOD -*d*4) δ 8.13 (d, *J* = 9.5 Hz, 1H, H-5), 7.54 (d, *J* = 2.6 Hz, 1H, H-7), 7.43 – 7.31 (m, 1H, H-8), 6.43 (s, 1H, H-3), 2.96 (t, *J* = 7.9 Hz, 2H, H-9), 1.86-1.71 (m, 2H, H-10), 1.54 – 1.24 (m, 12H, H-11-16), 0.92 (t, *J* = 6.9 Hz, 3H, H-17).

^{13}C NMR (101 MHz, MeOD- *d*4) δ 157.04 (C-2), 153.35 (C-6), 147.46 (C-8a), 136.40 (C-4a), 124.22 (C-7), 119.47 (C-8), 107.31 (C-5), 106.06 (C-3), 33.02 (C-9), 32.34, 30.59, 30.50, 30.41 (2C), 28.64, 23.72 (C-10-16), 14.41 (C-17).

TOF-HRMS: m/z = 304.1904 $[M+H]^+$, calc. for $C_{18}H_{25}NO_3 + H^+$ = 304.1907; 326.1928 $[M+Na]^+$, calc. for $C_{18}H_{25}NO_3 + Na^+$ = 326.1726.

Annex I: NMR spectra

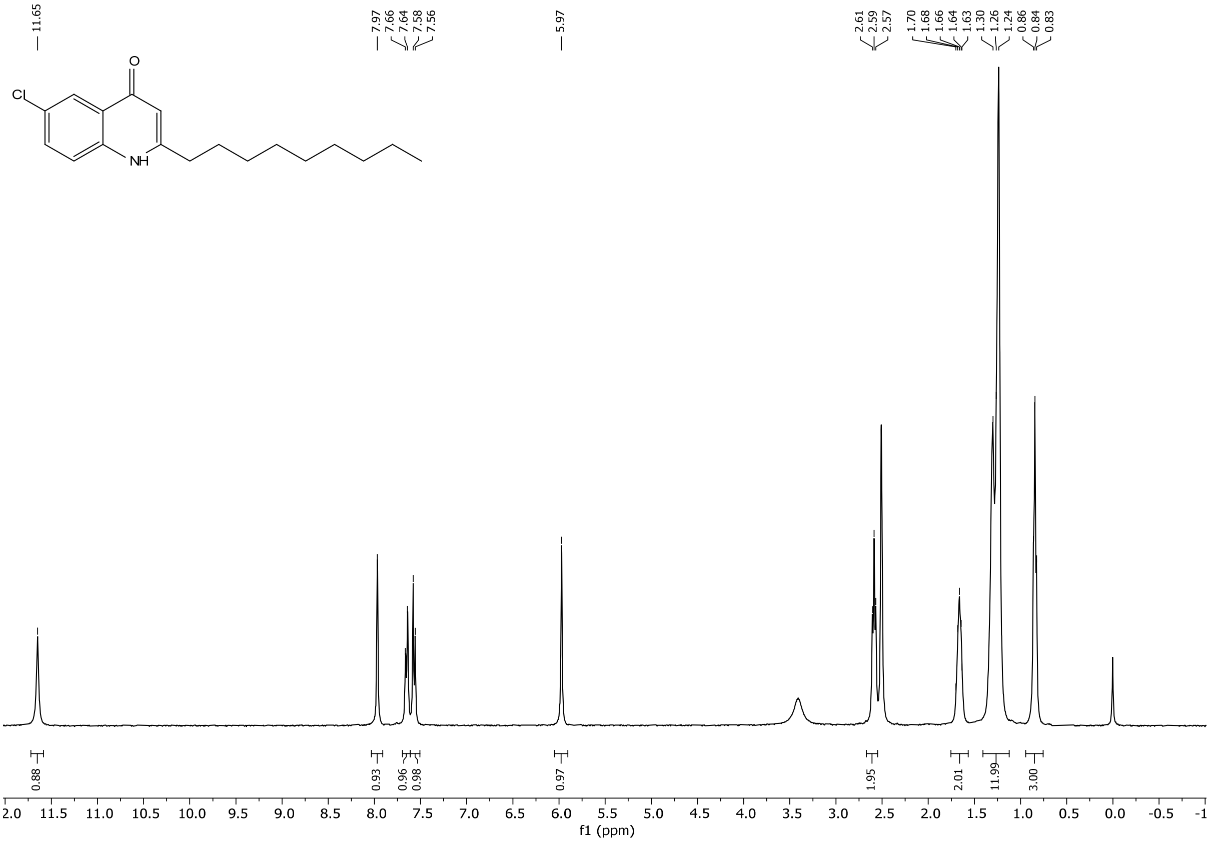

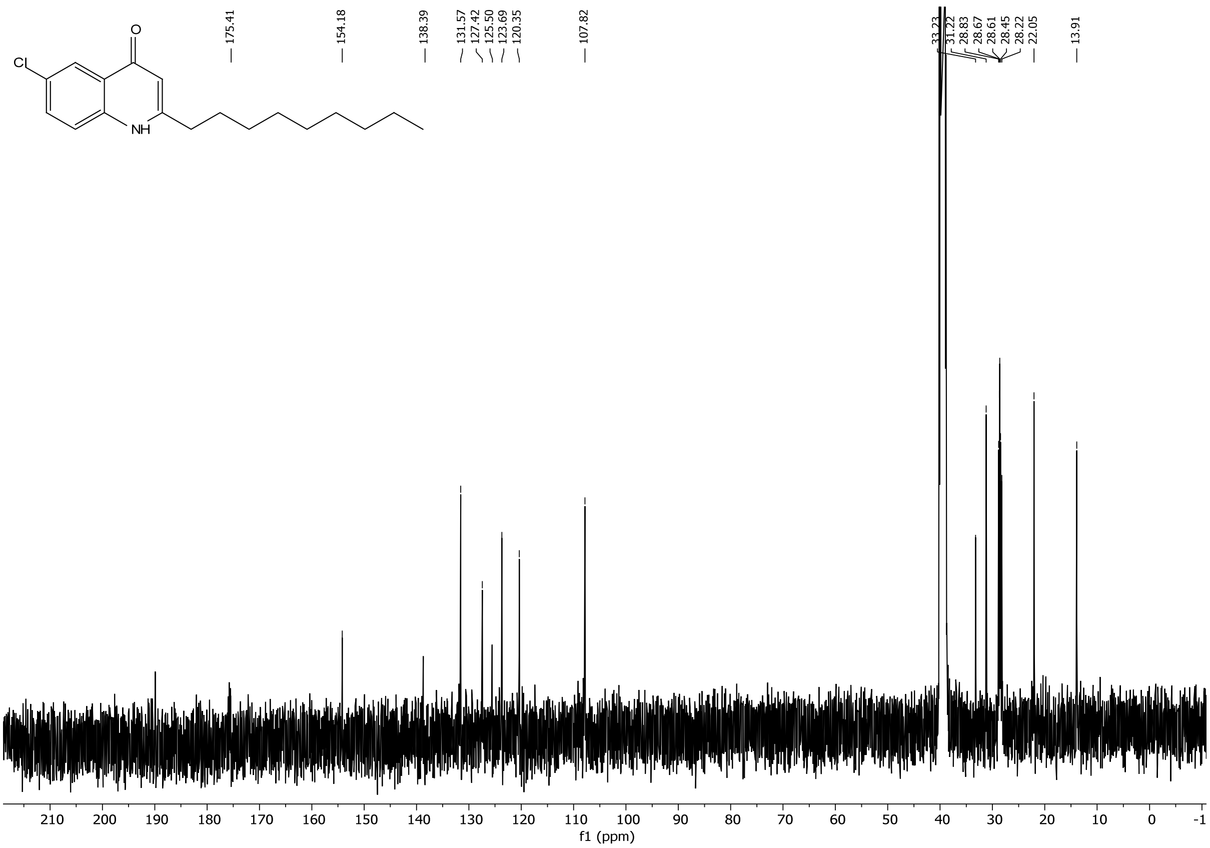

Figure SA1. ^{1}H-NMR and ^{13}C-NMR spectra of **6Cl-NQ** in DMSO-d_6.

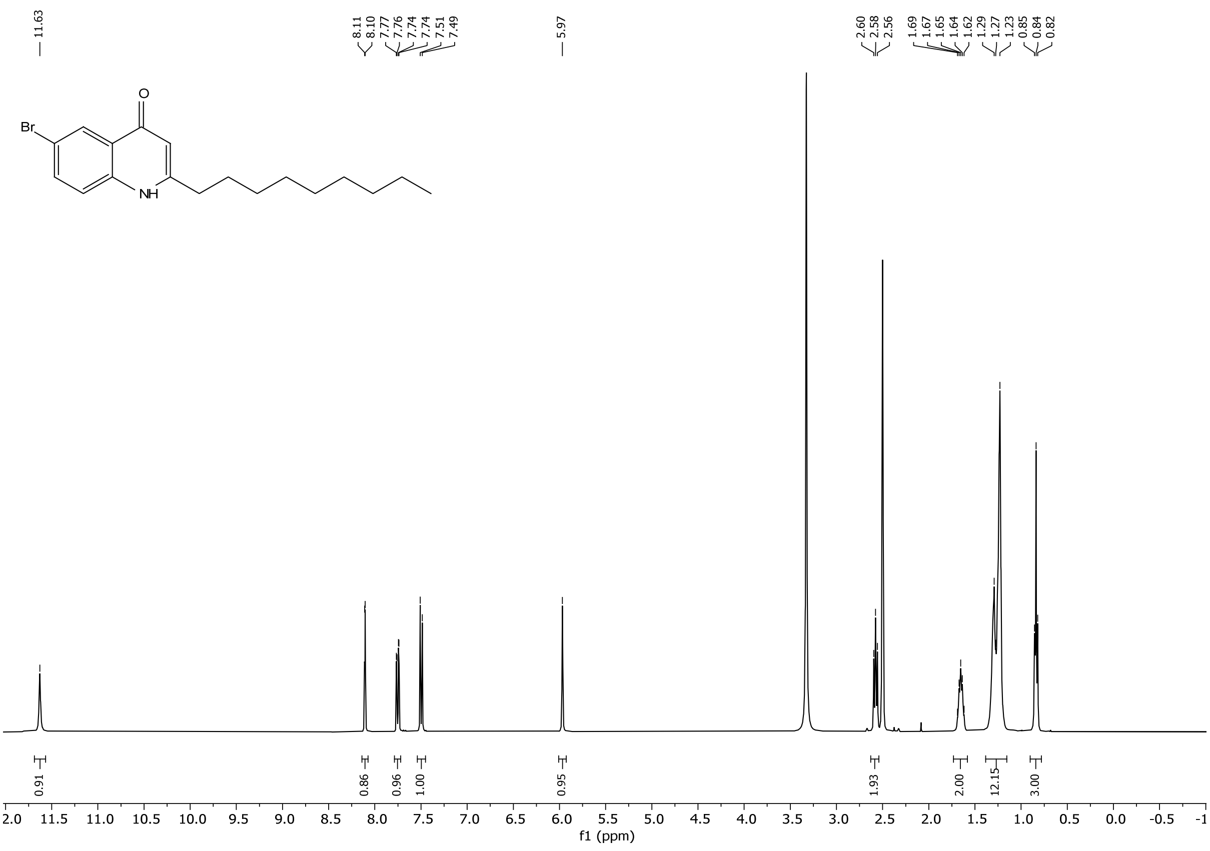

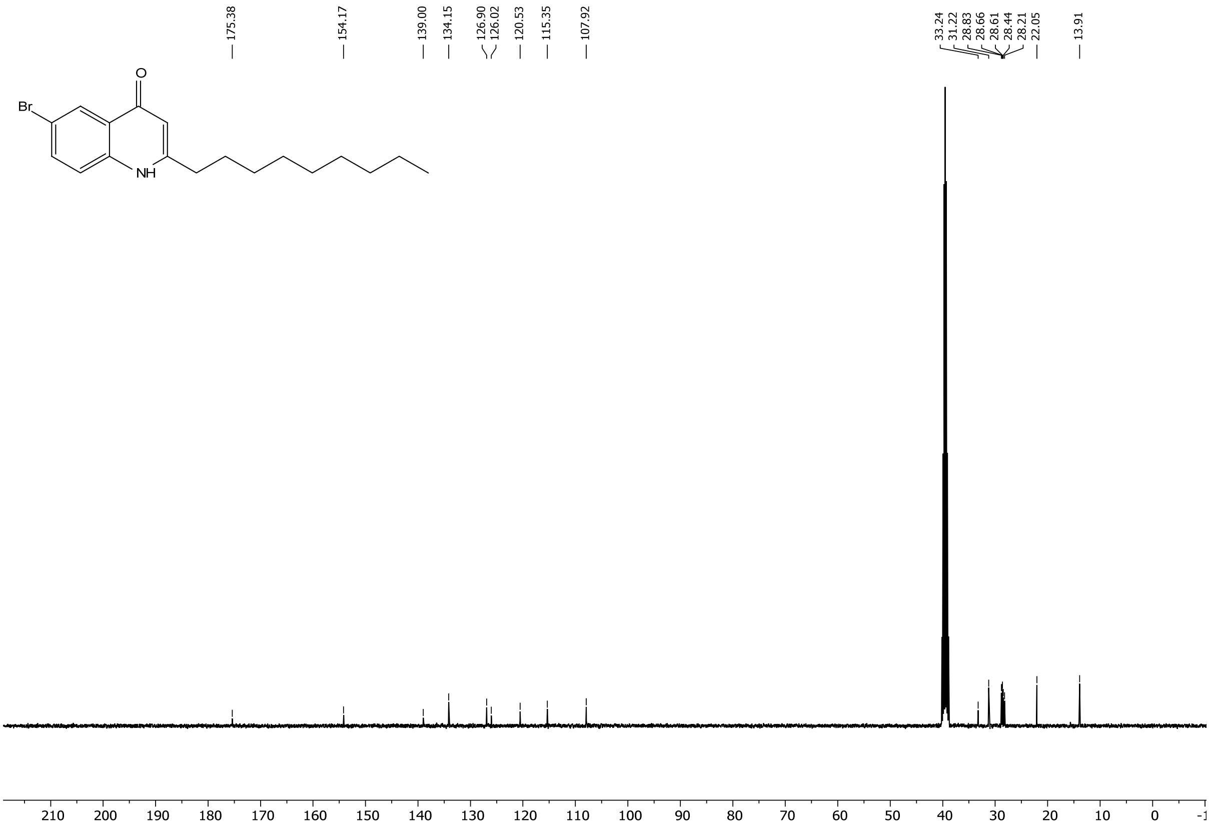

Figure SA2. ^{1}H-NMR and ^{13}C-NMR spectra of **6Br-NQ** in DMSO-d_6.

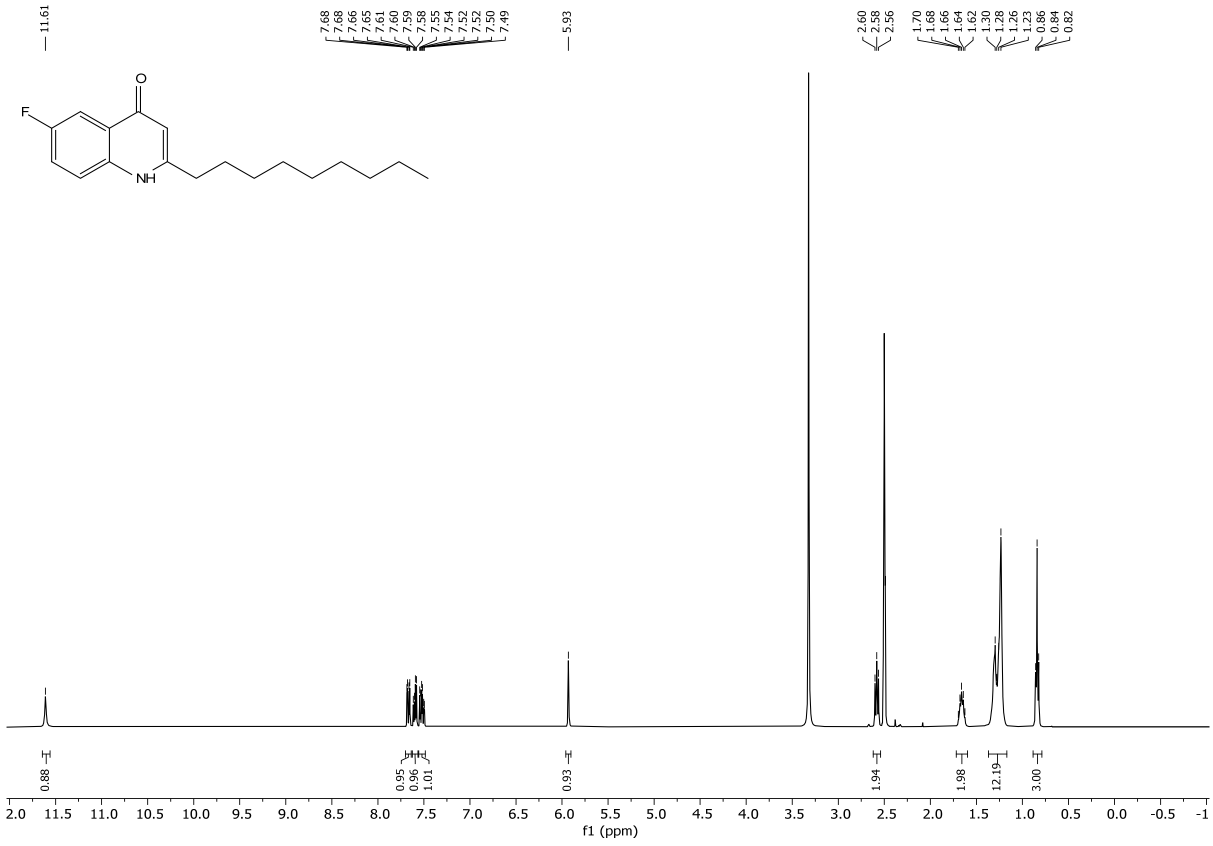

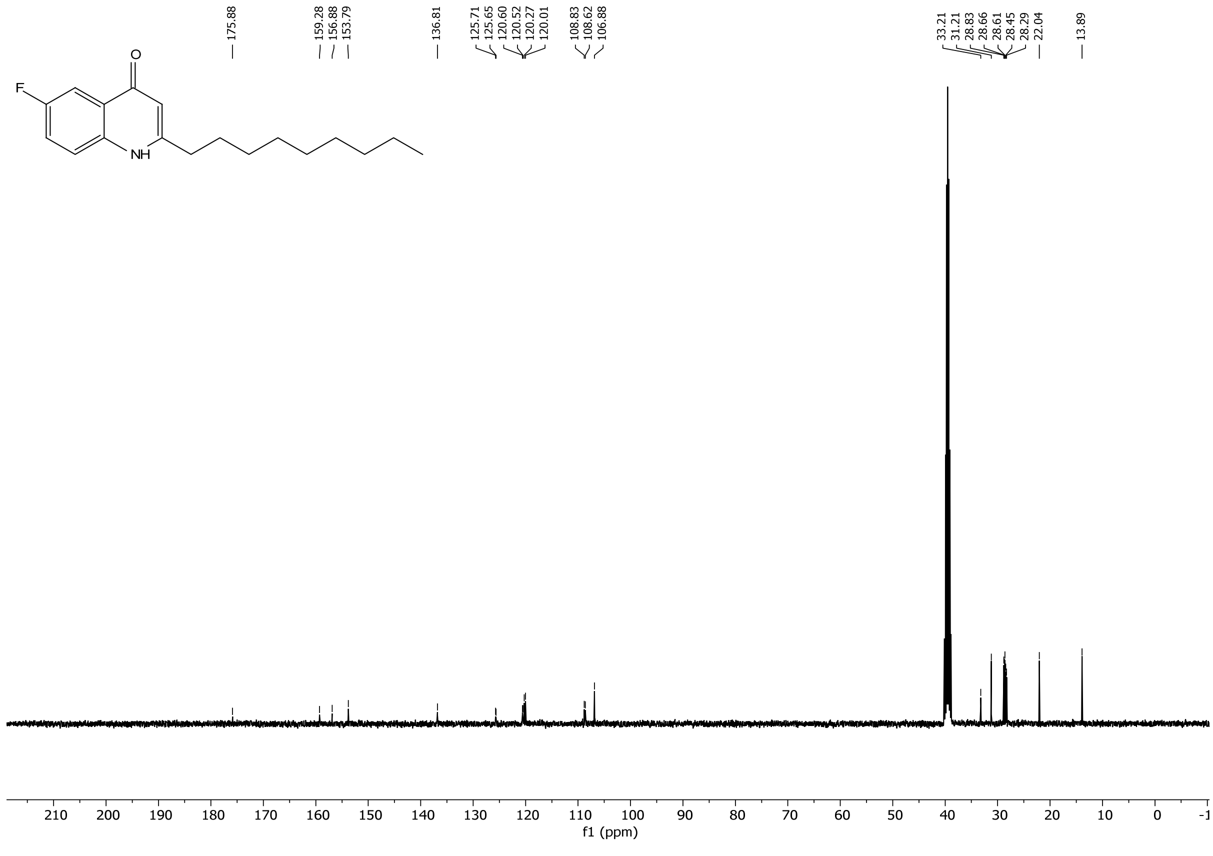

Figure SA3. ^{1}H-NMR and ^{13}C-NMR spectra of **6F-NQ** in DMSO-d_6.

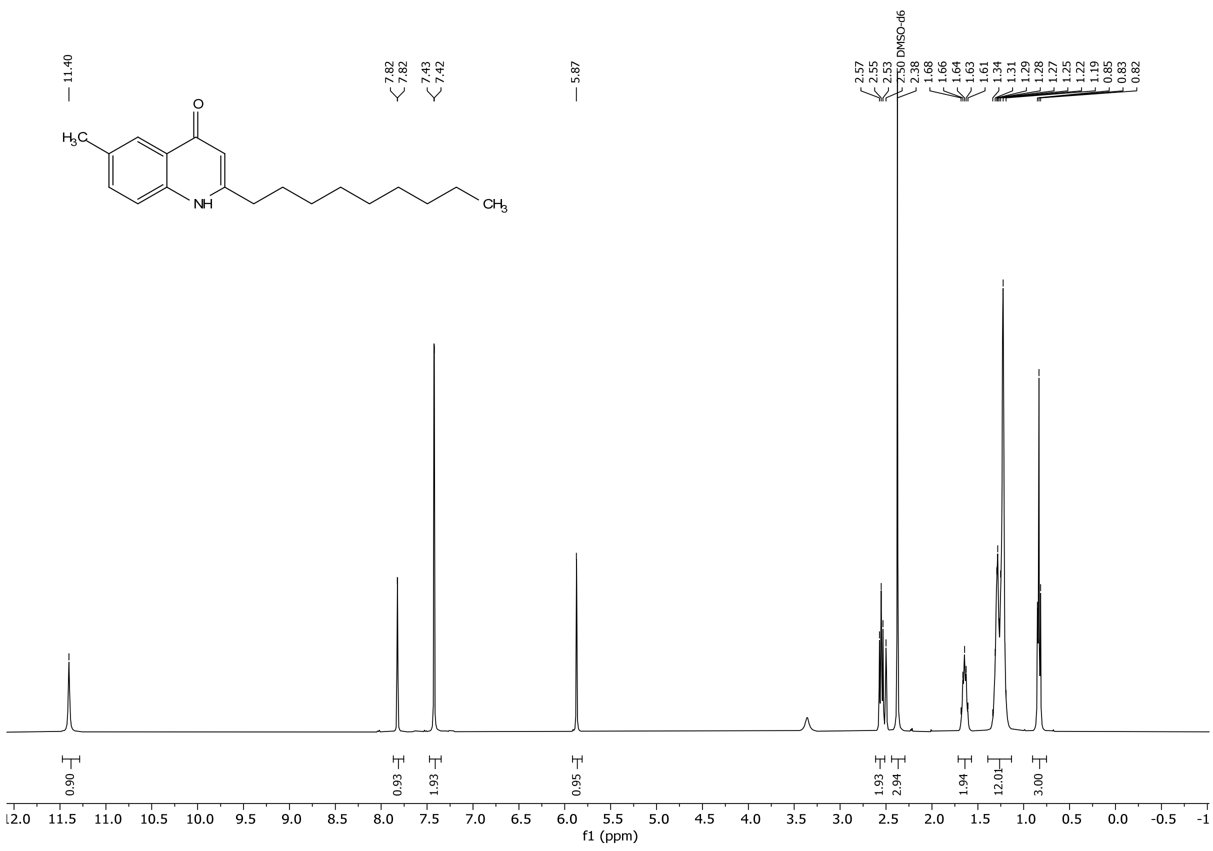

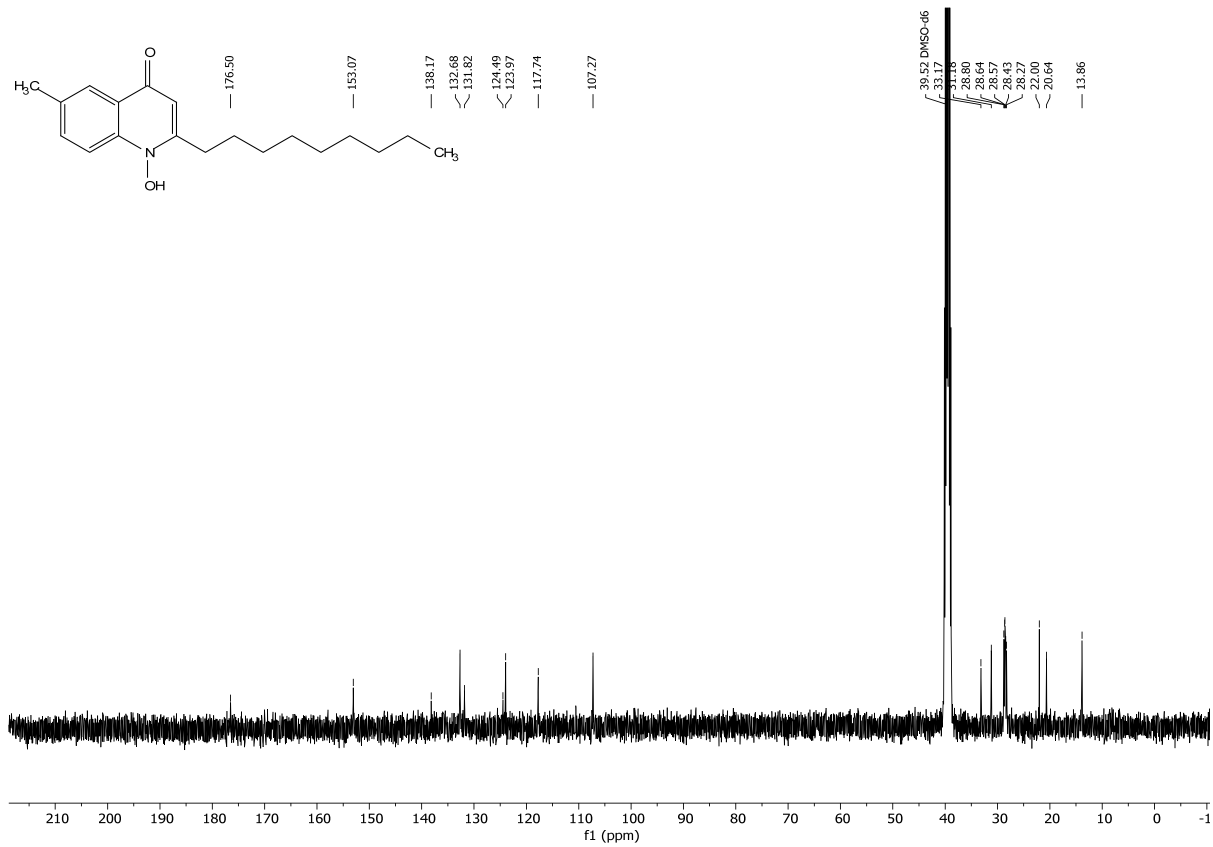

Figure SA4. ^{1}H-NMR and ^{13}C-NMR spectra of **6Me-NQ** in DMSO-d_6.

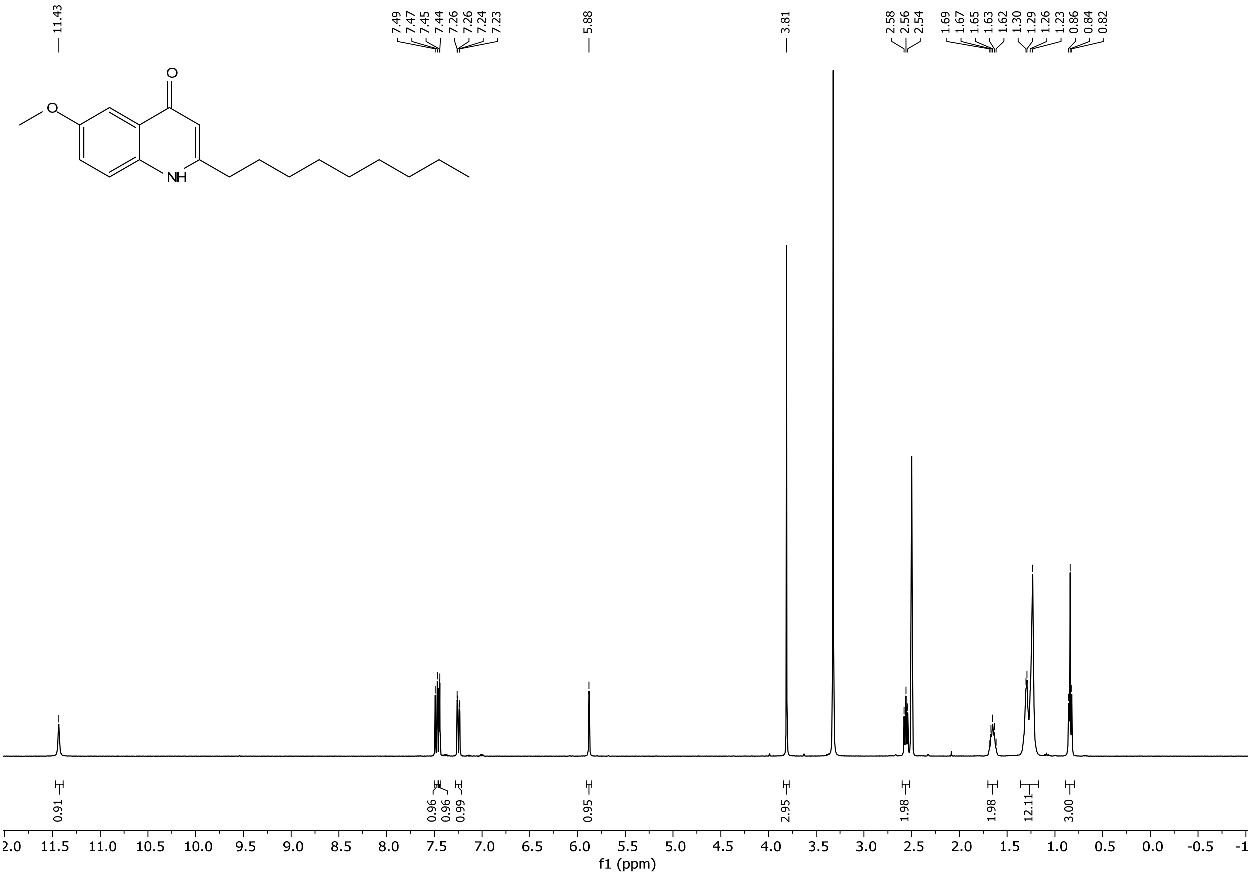

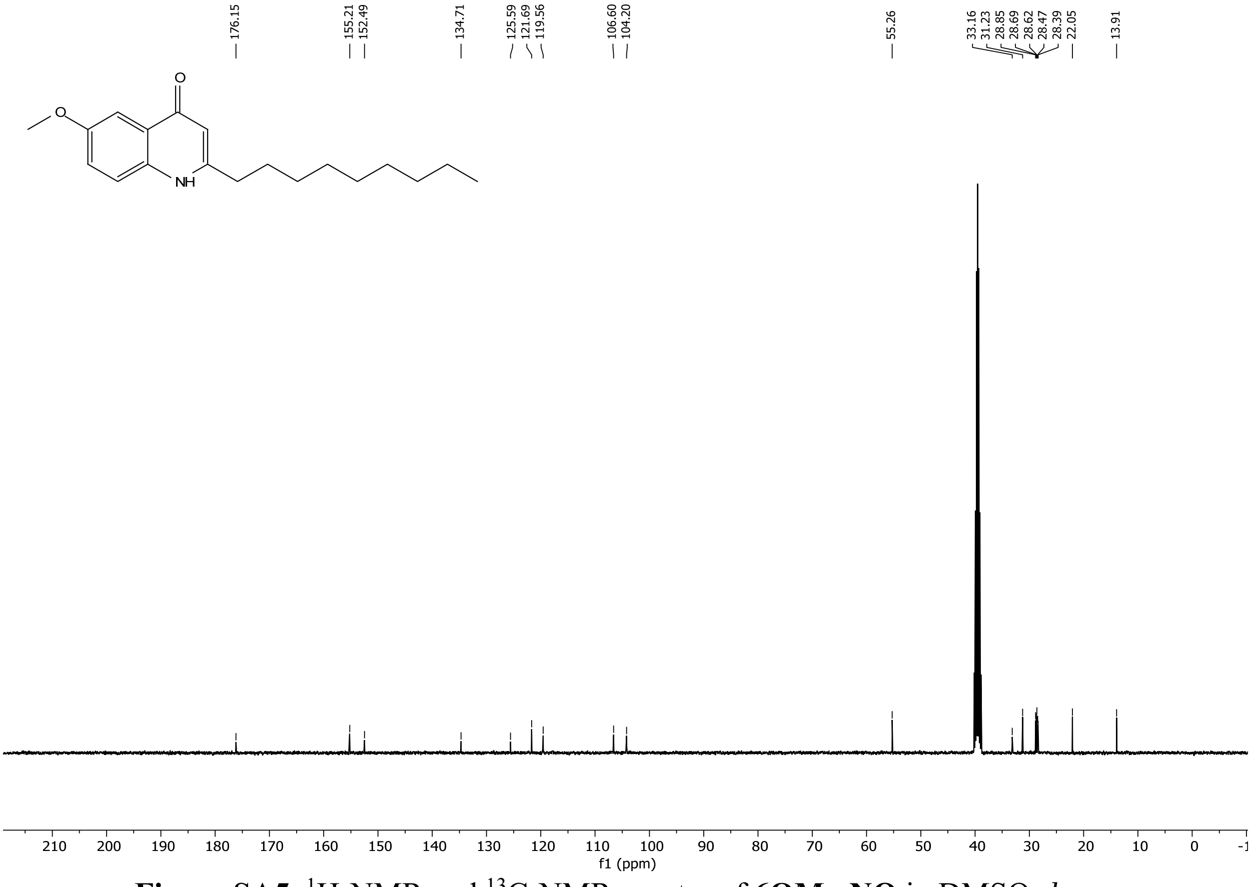

Figure SA5. ^{1}H-NMR and ^{13}C-NMR spectra of **6OMe-NQ** in DMSO-d_6.

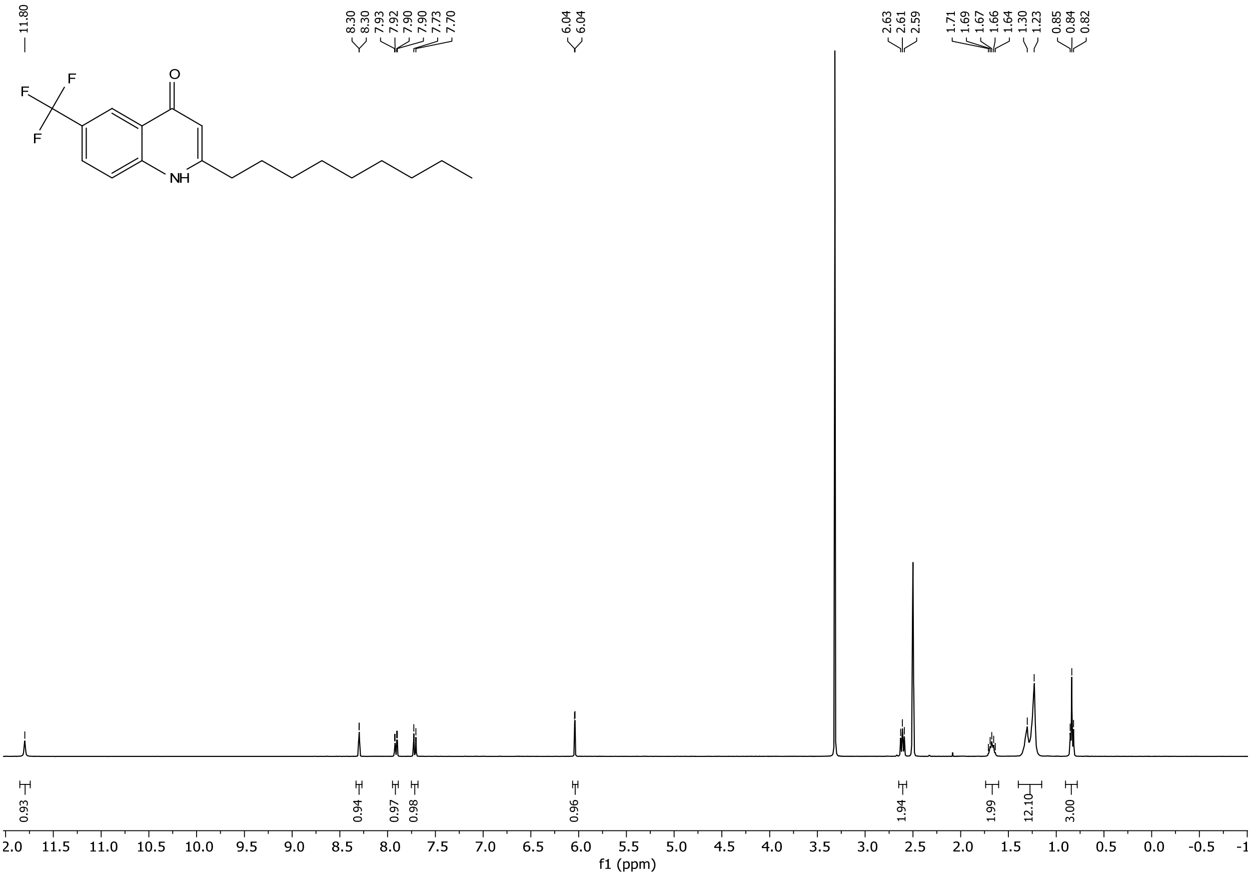

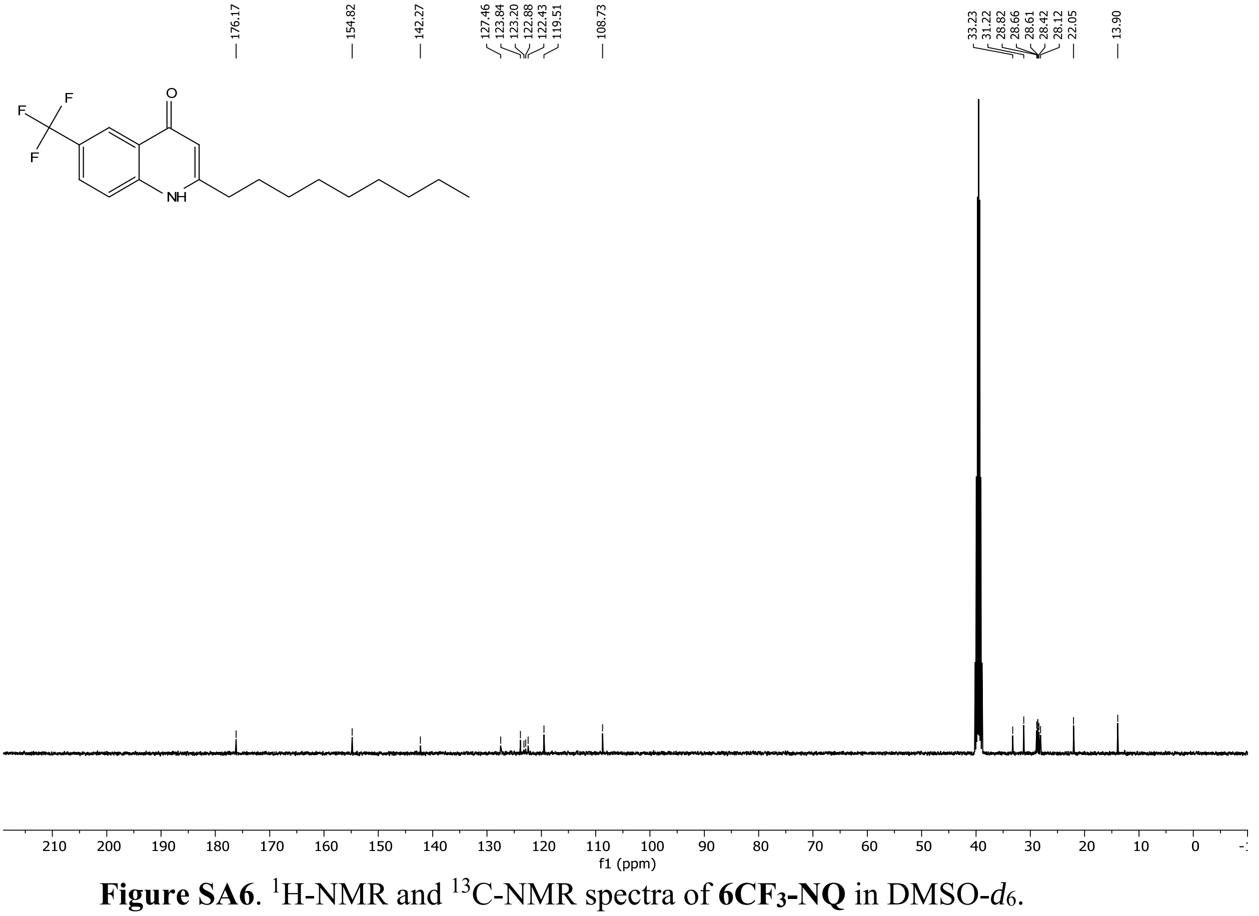

Figure SA6. ^{1}H-NMR and ^{13}C-NMR spectra of **6CF$_3$-NQ** in DMSO-d_6.

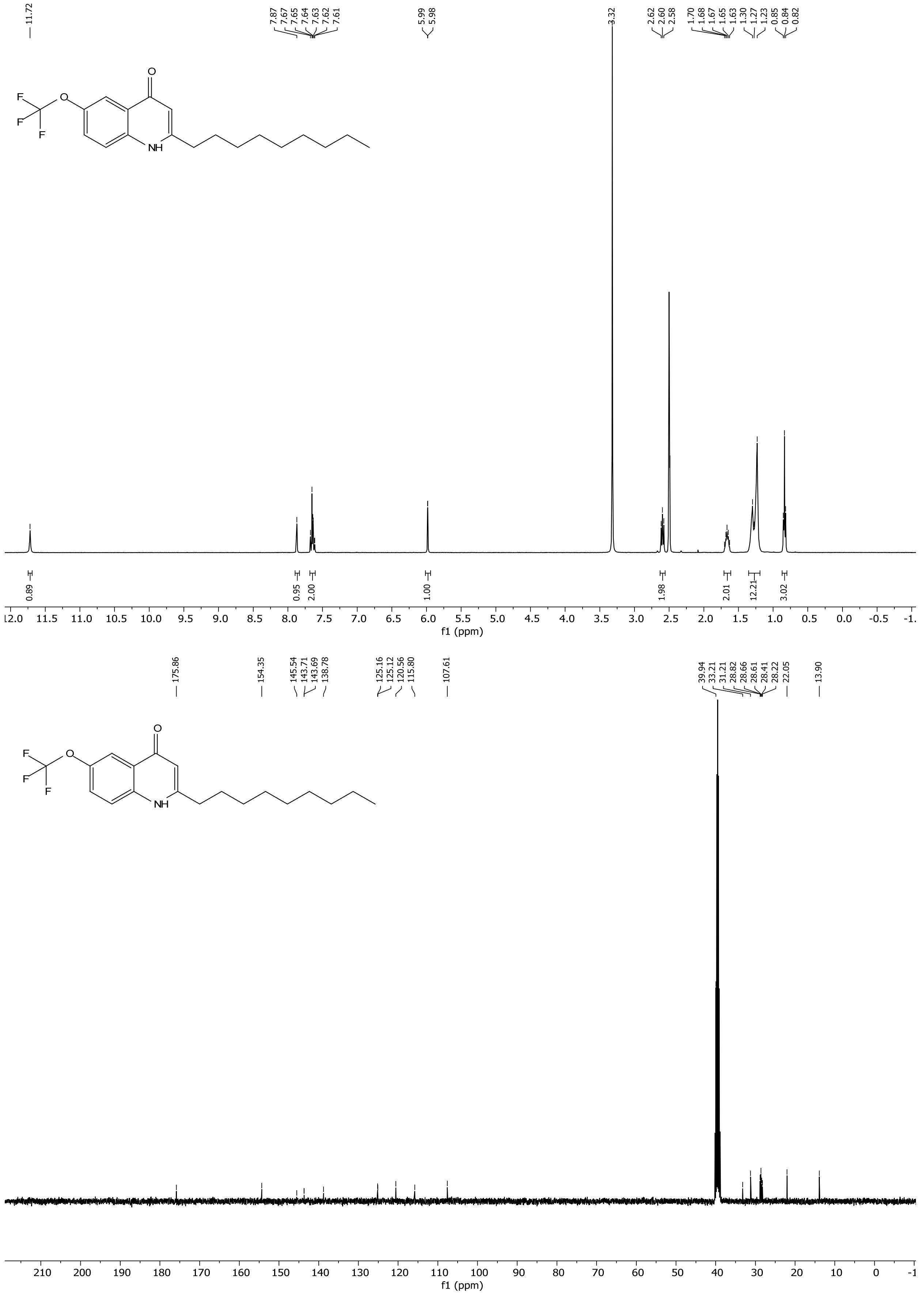

Figure SA7. ^{1}H-NMR and ^{13}C-NMR spectra of **6OCF$_3$-NQ** in DMSO-d_6.

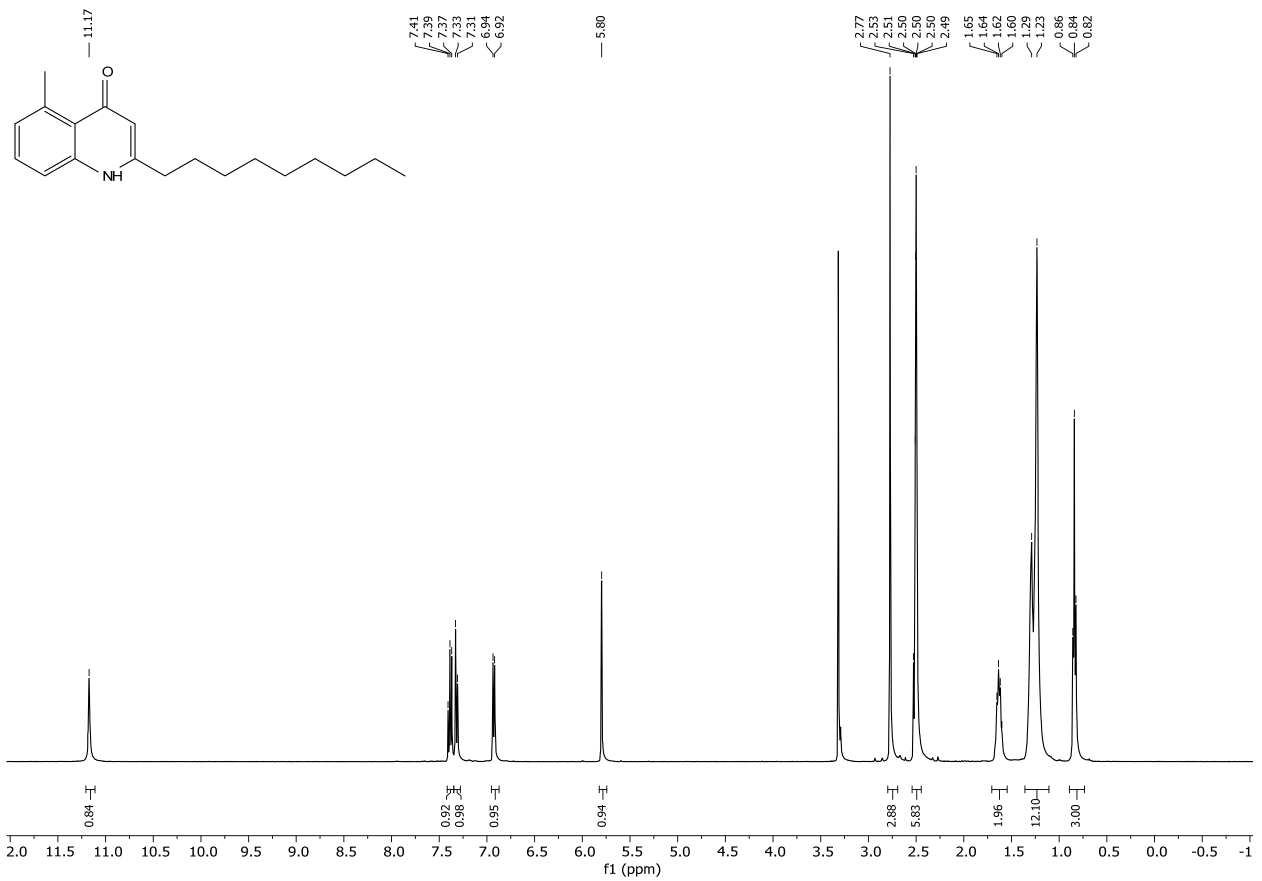

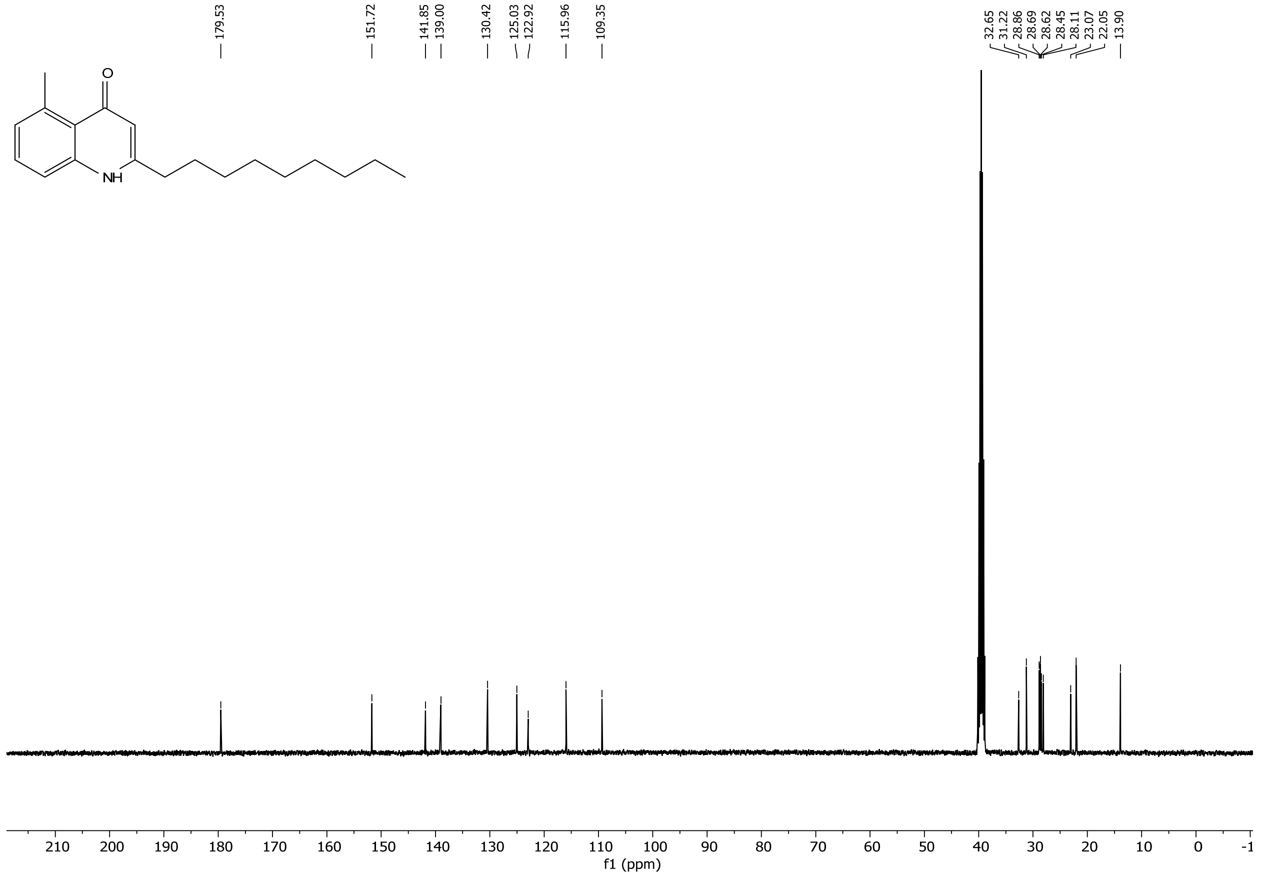

Figure SA8. ^{1}H-NMR and ^{13}C-NMR spectra of **5Me-NQ** in DMSO-d_6.

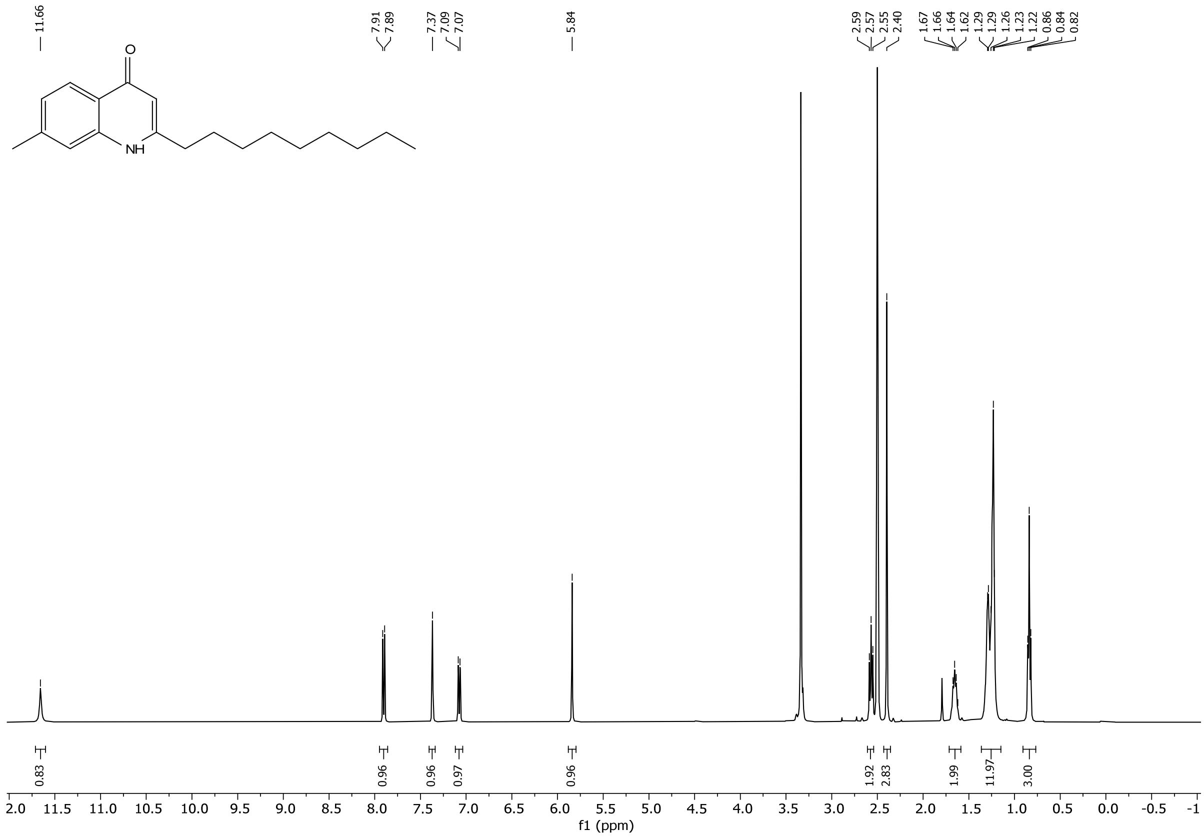

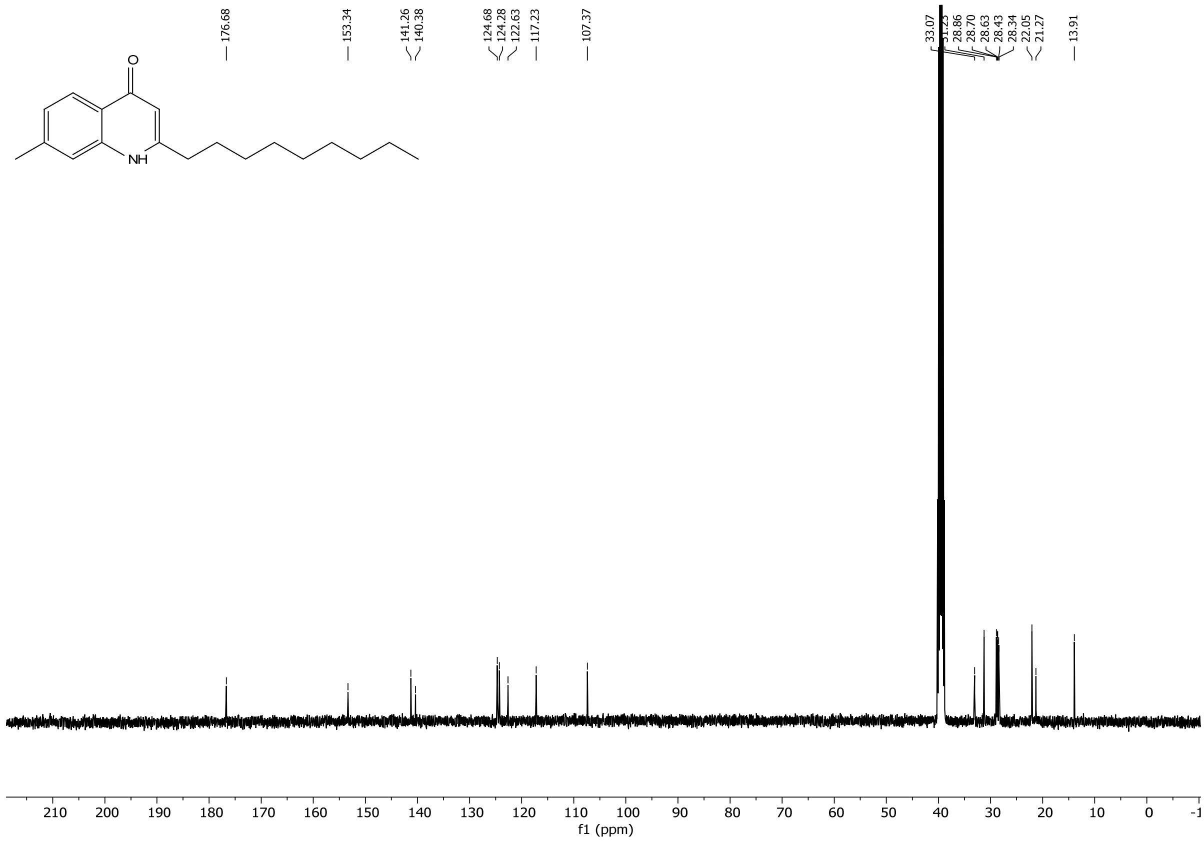

Figure SA9. ^{1}H-NMR and ^{13}C-NMR spectra of **7Me-NQ** in DMSO-d_6.

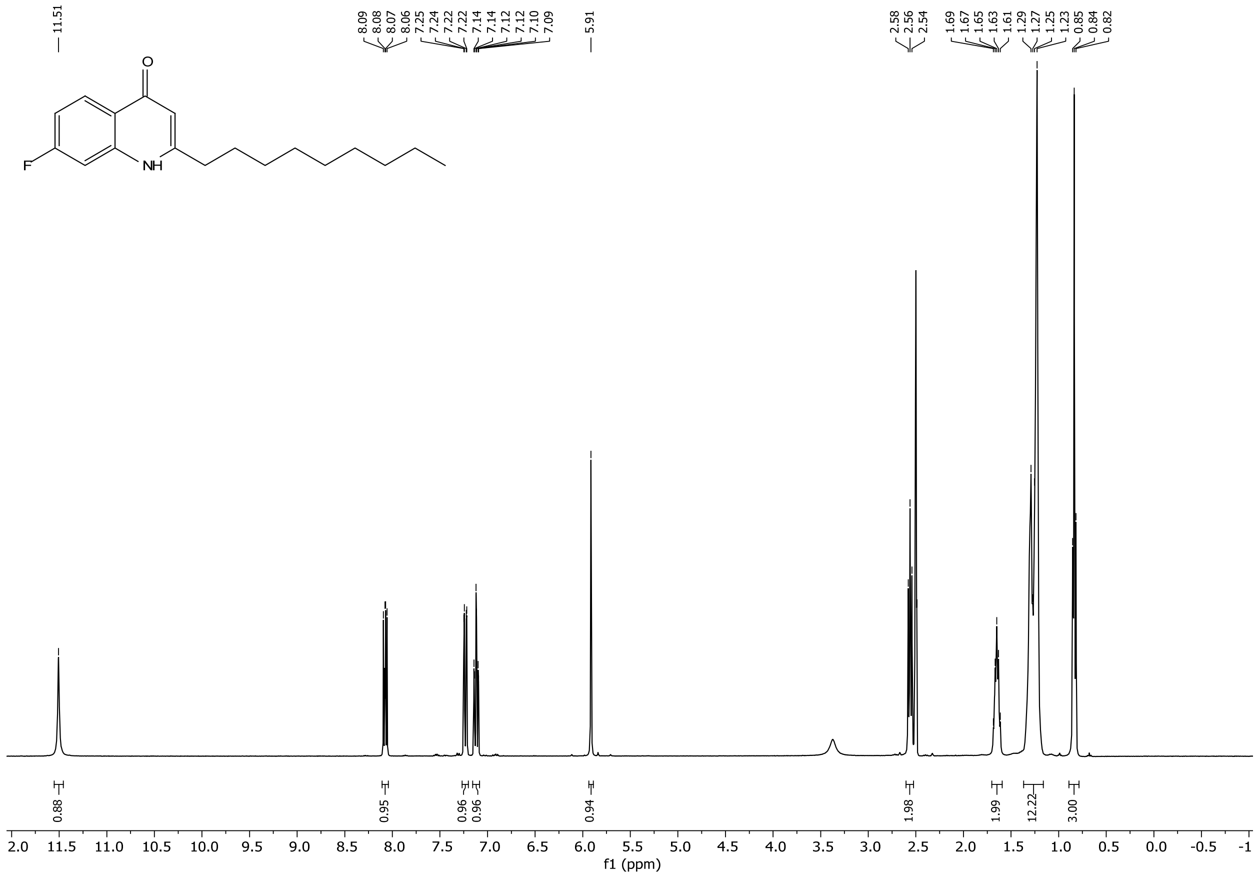

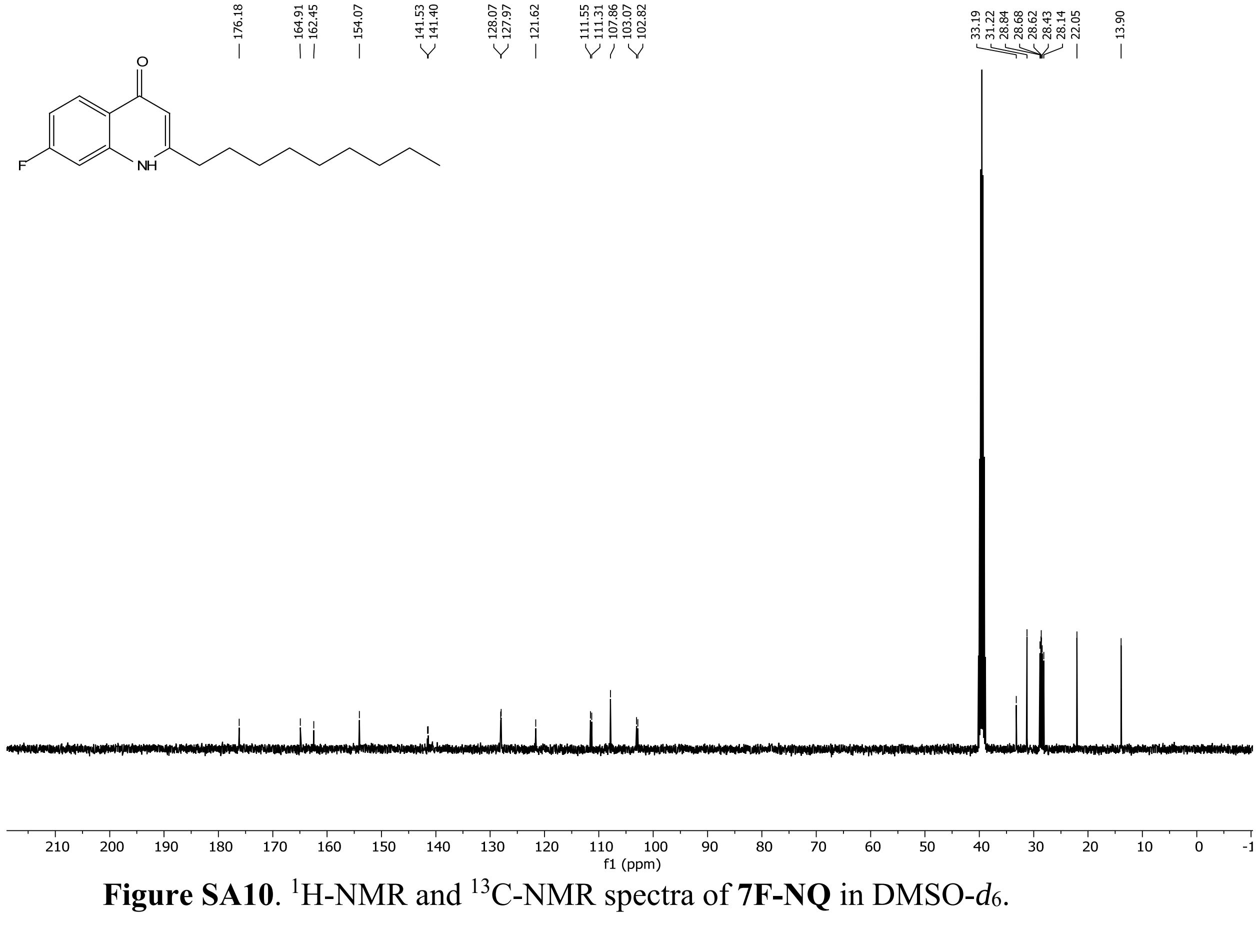

Figure SA10. ^{1}H-NMR and ^{13}C-NMR spectra of **7F-NQ** in DMSO-d_6.

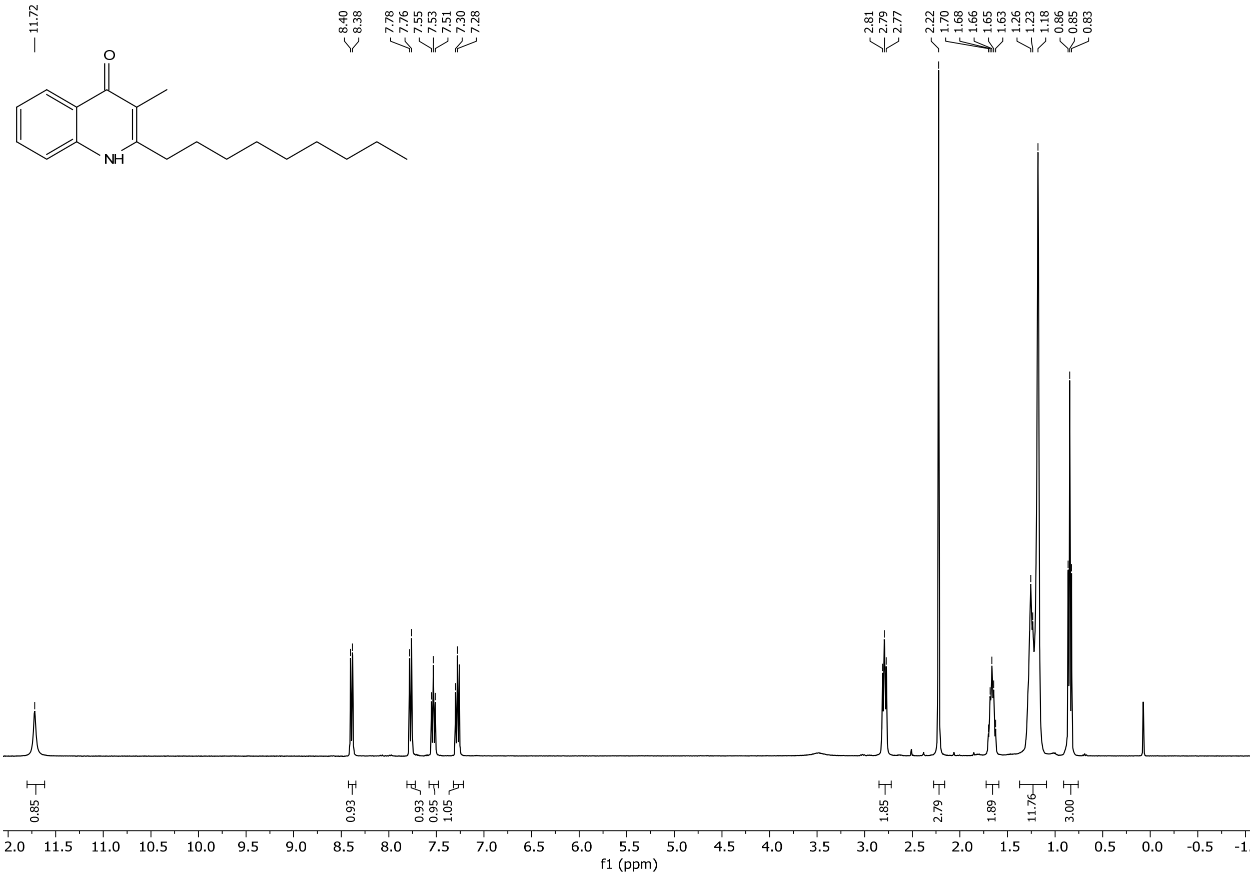

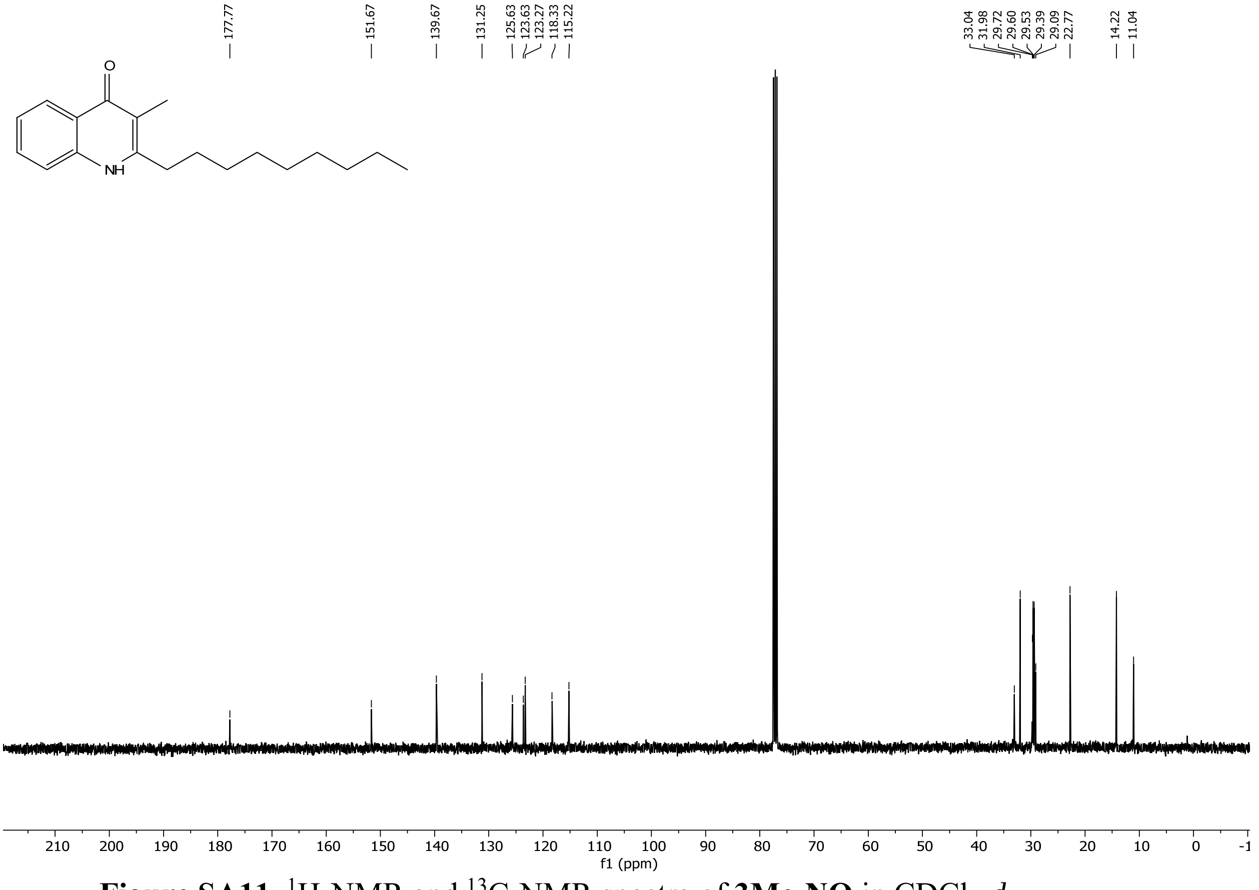

Figure SA11. ^{1}H-NMR and ^{13}C-NMR spectra of **3Me-NQ** in $CDCl_3$-*d*.

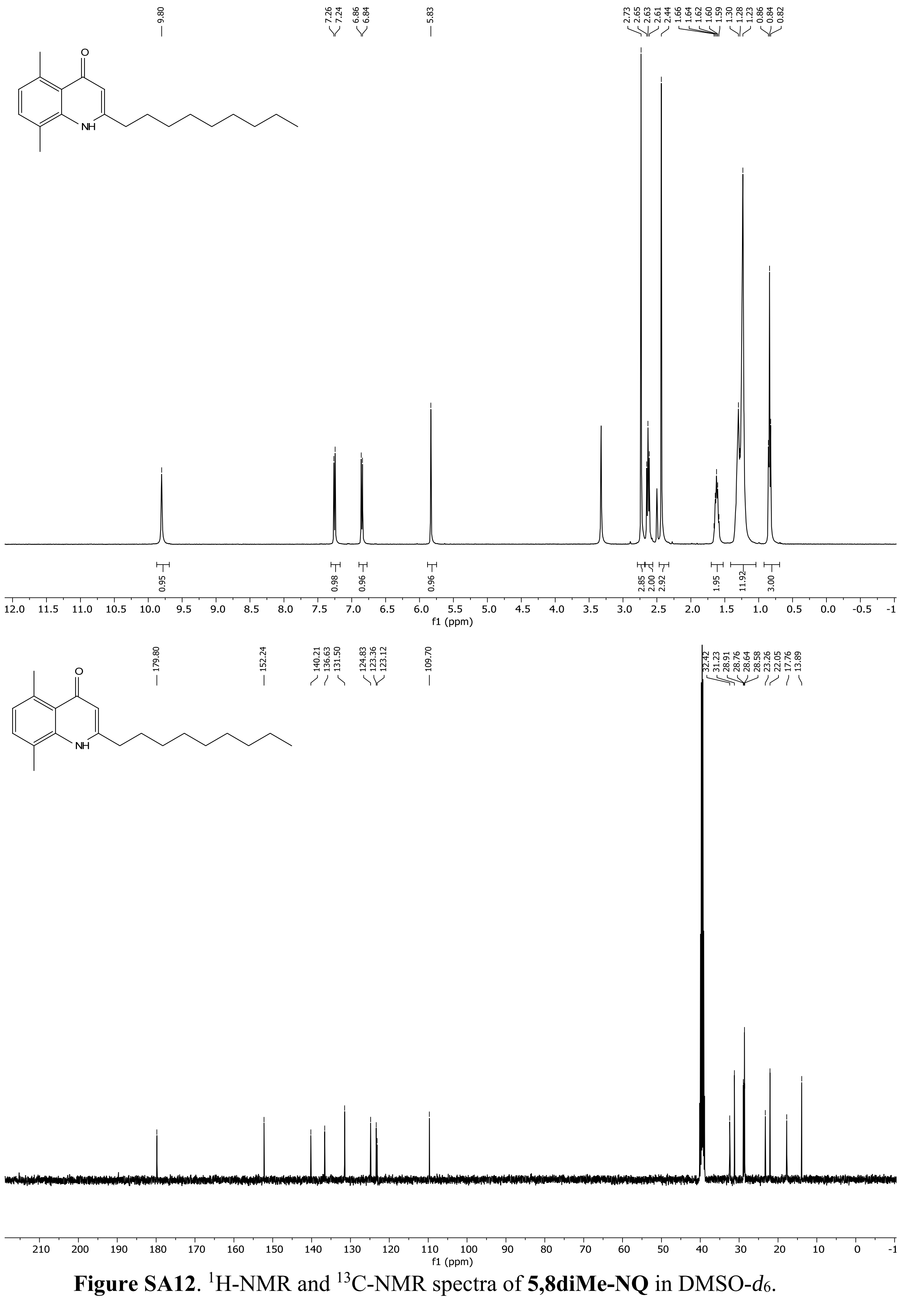

Figure SA12. ^{1}H-NMR and ^{13}C-NMR spectra of **5,8diMe-NQ** in DMSO-d_6.

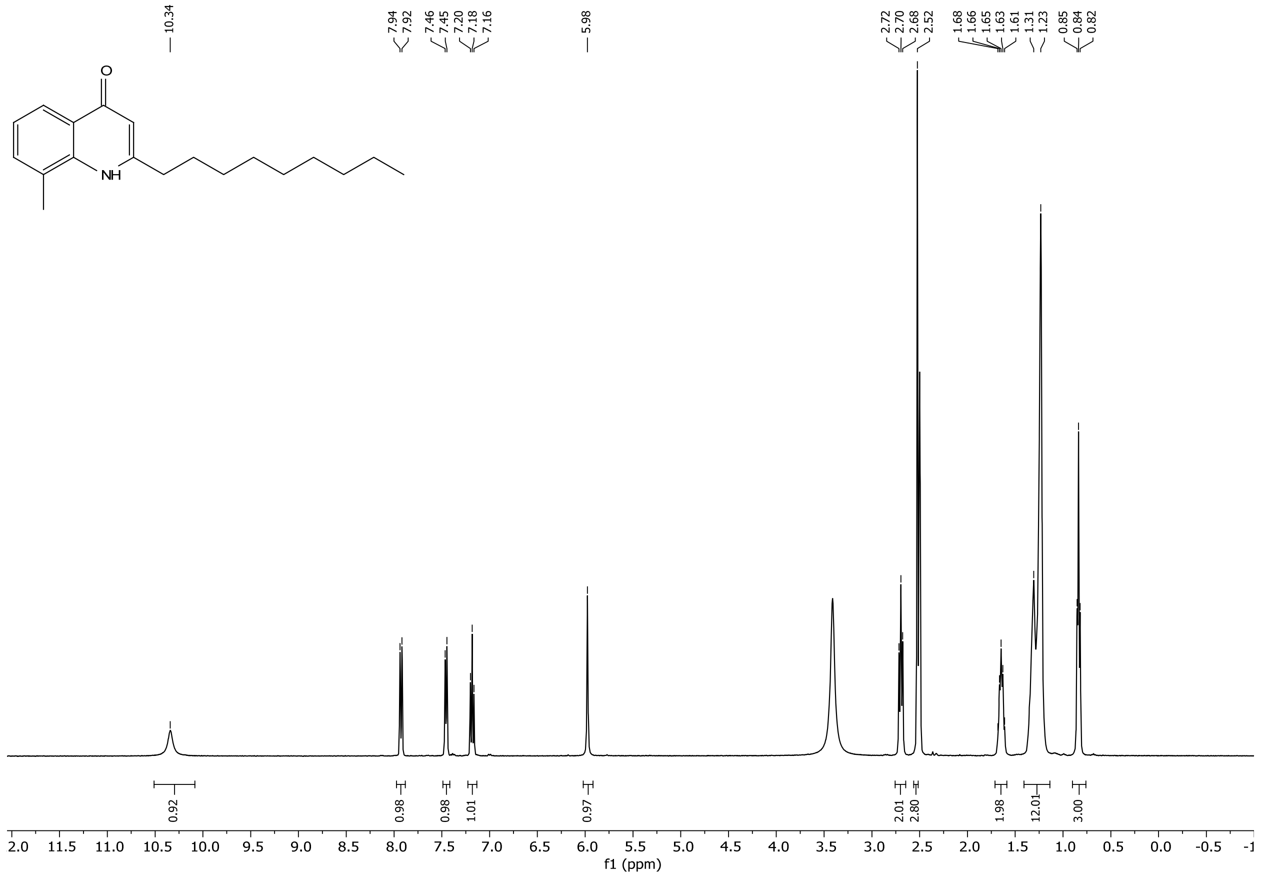

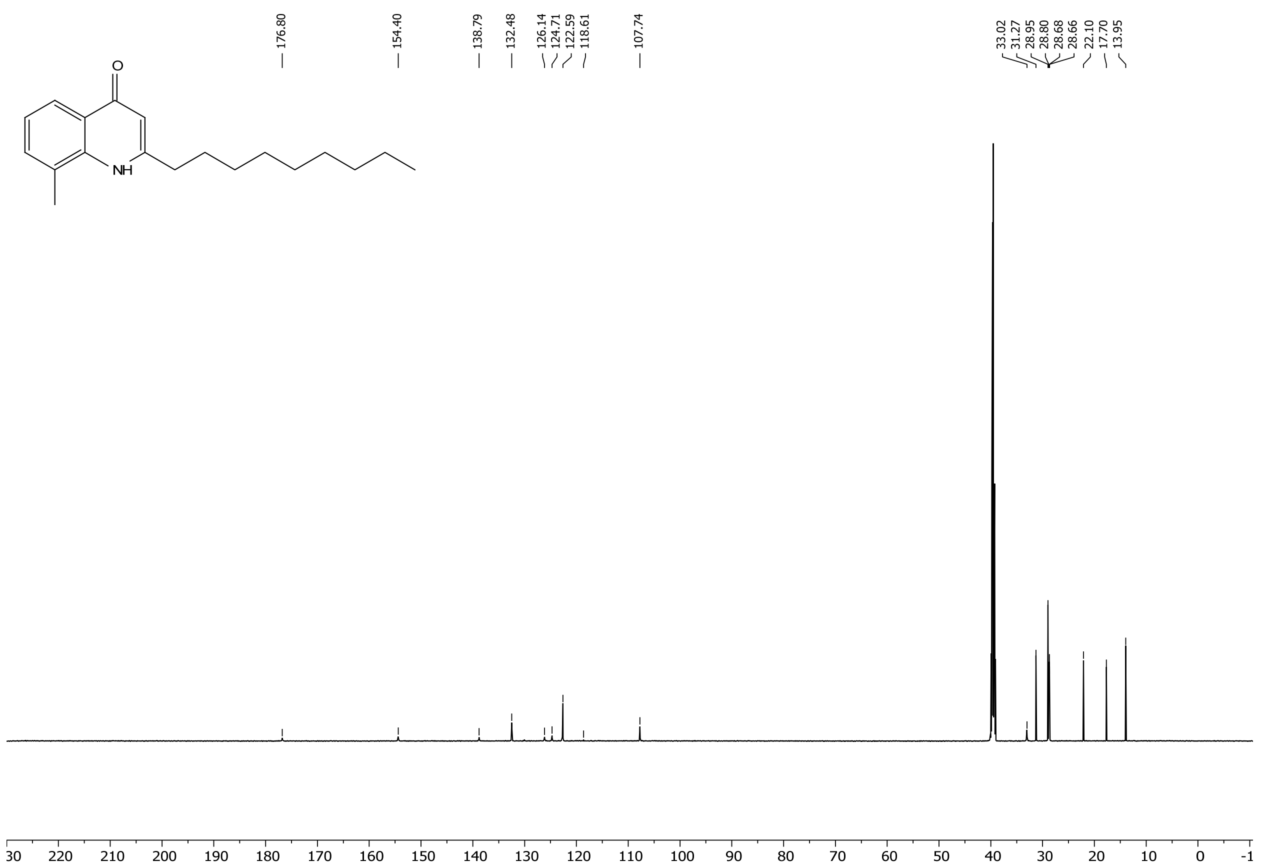

Figure SA13. ^{1}H-NMR and ^{13}C-NMR spectra of **8Me-NQ** in DMSO-d_6.

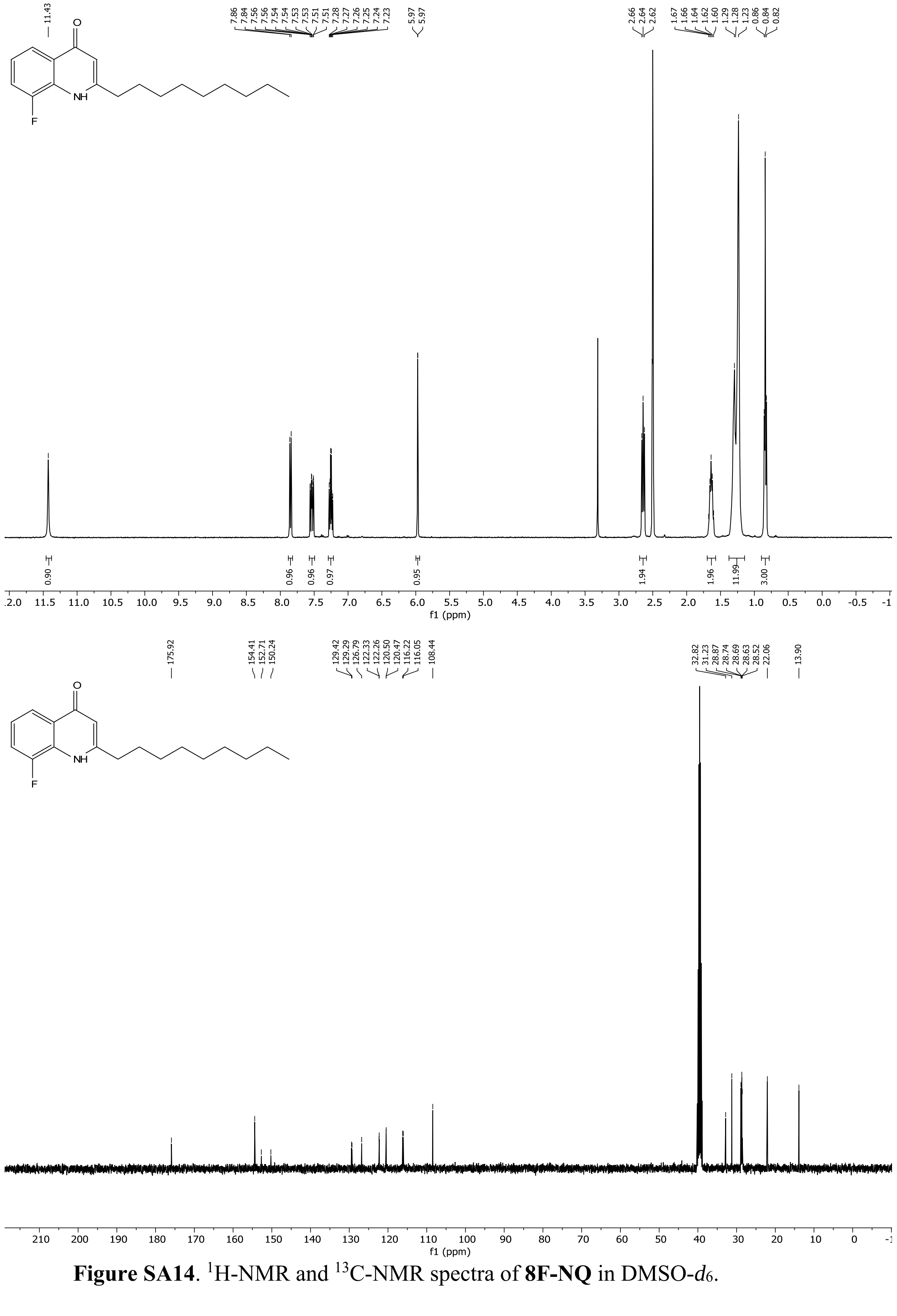

Figure SA14. ^{1}H-NMR and ^{13}C-NMR spectra of **8F-NQ** in DMSO-d_6.

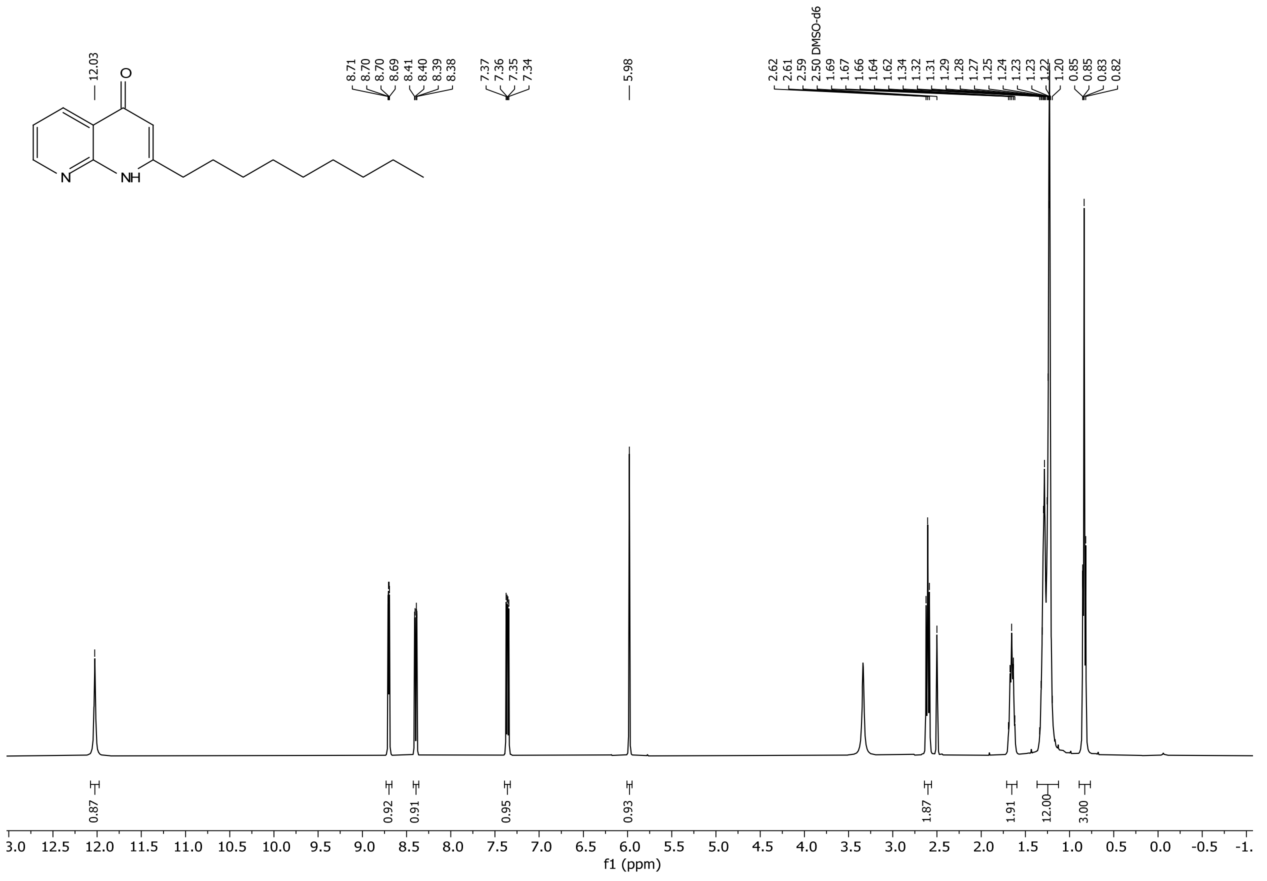

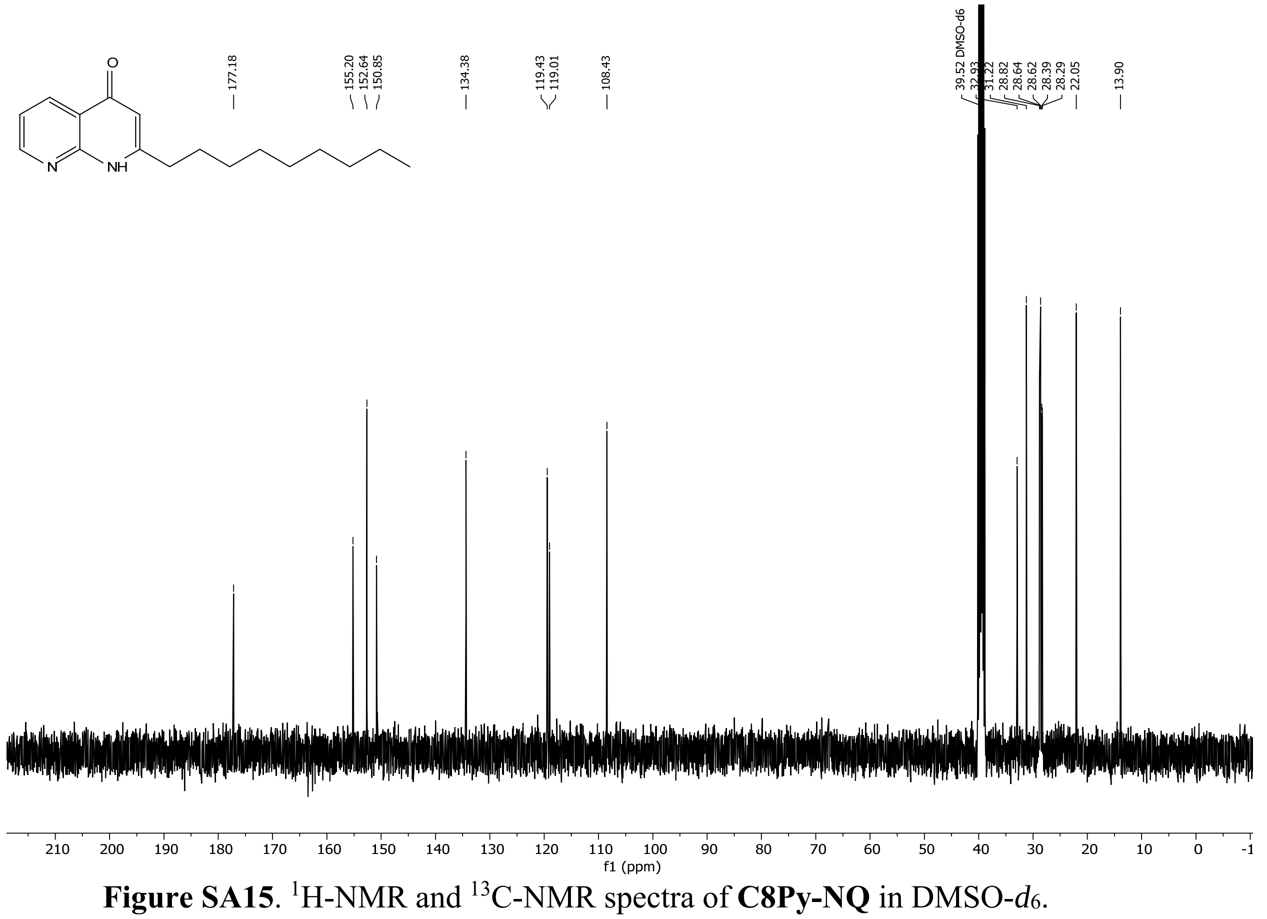

Figure SA15. ^{1}H-NMR and ^{13}C-NMR spectra of **C8Py-NQ** in DMSO-d_6.

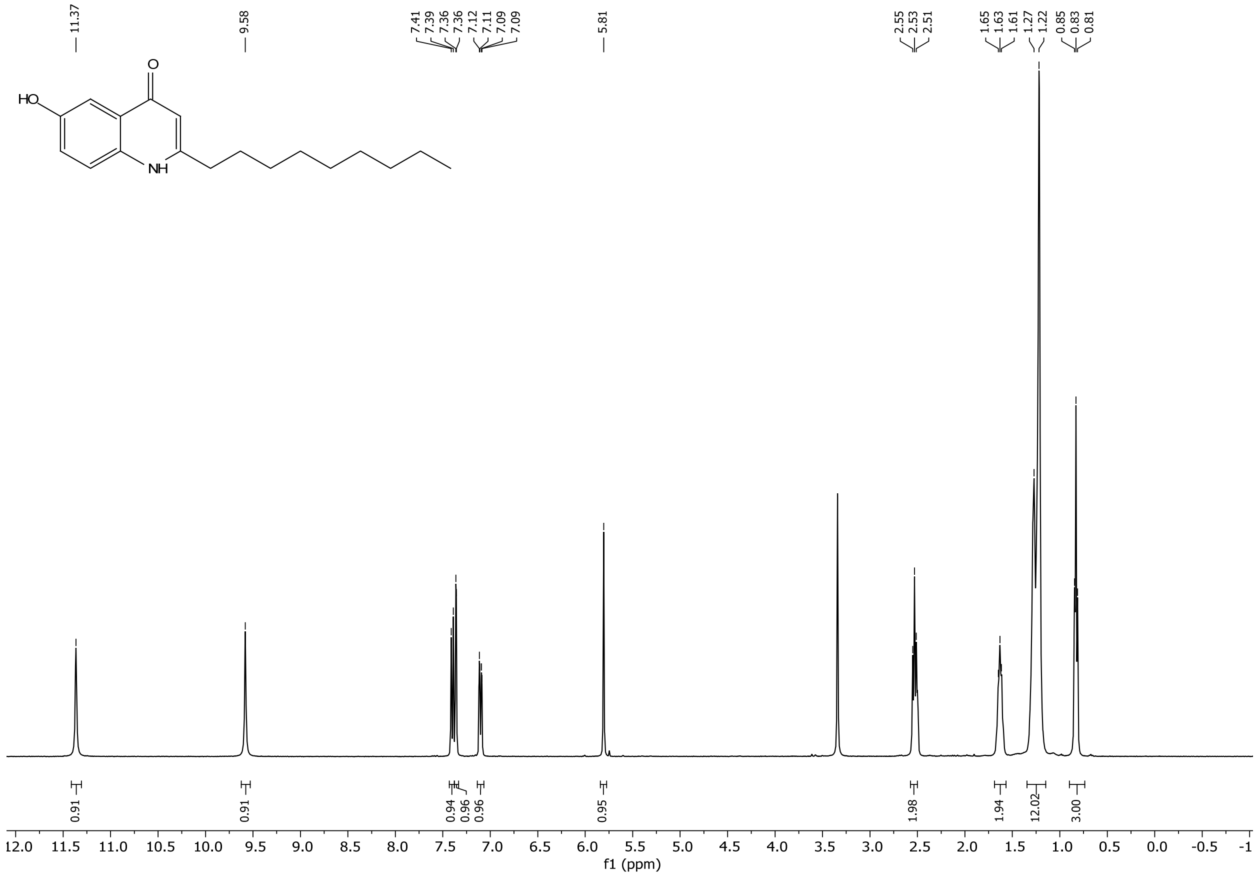

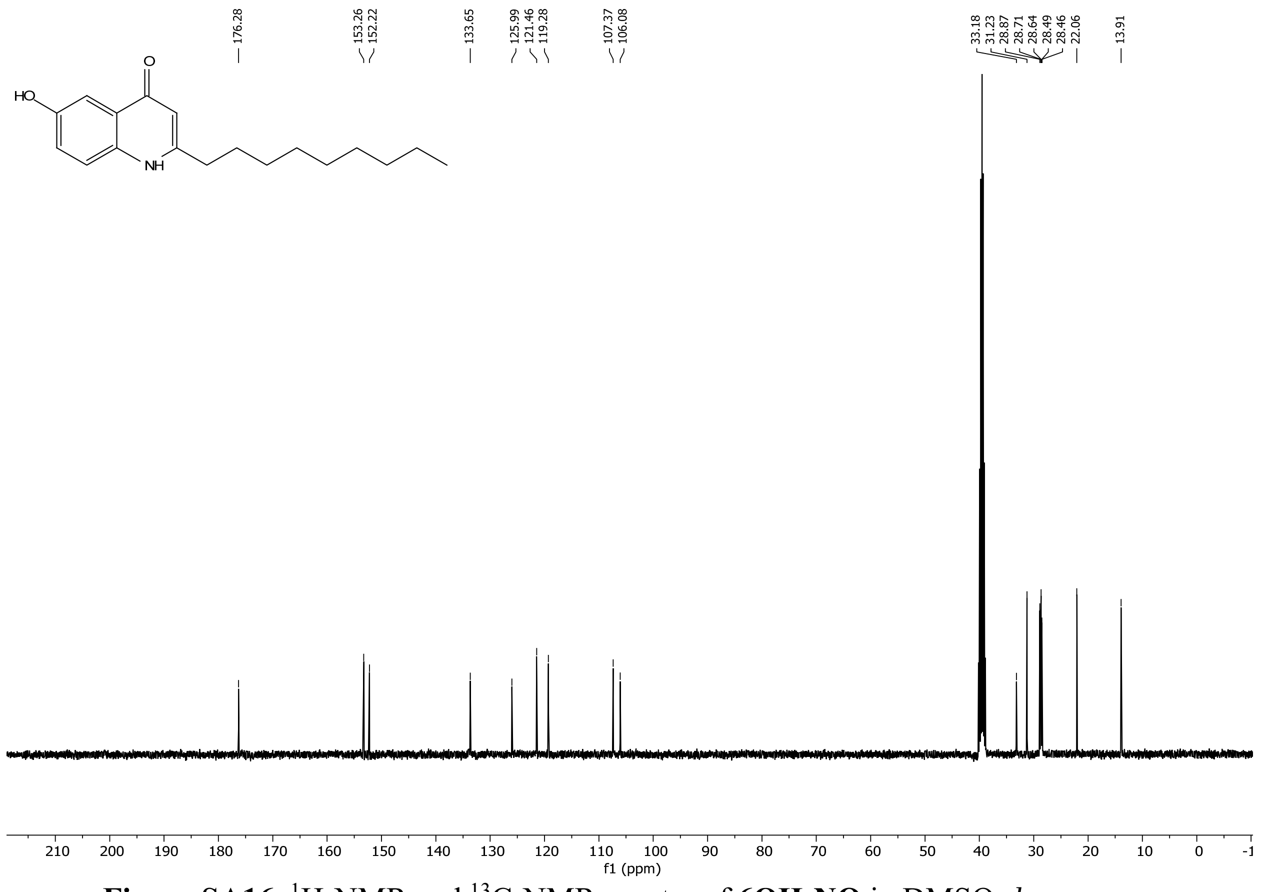

Figure SA16. ^{1}H-NMR and ^{13}C-NMR spectra of **6OH-NQ** in DMSO-d_6.

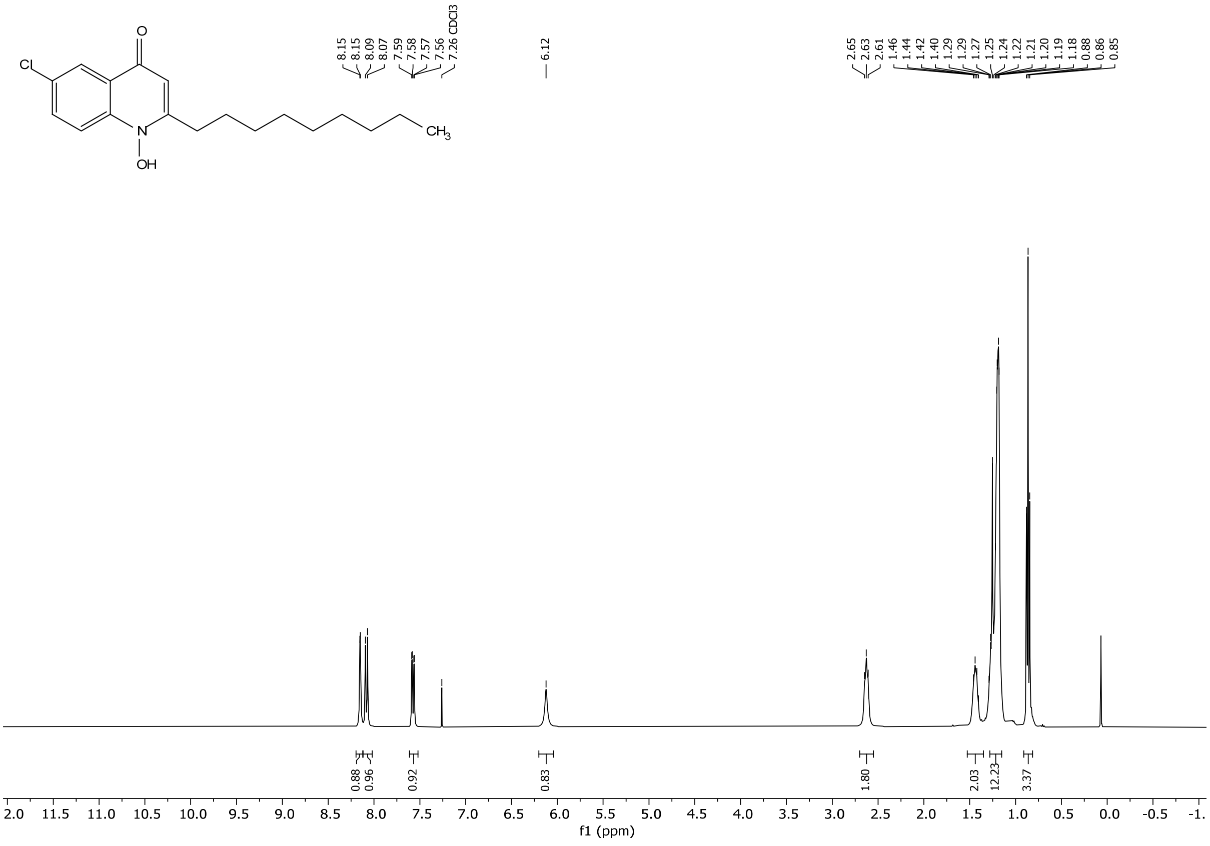

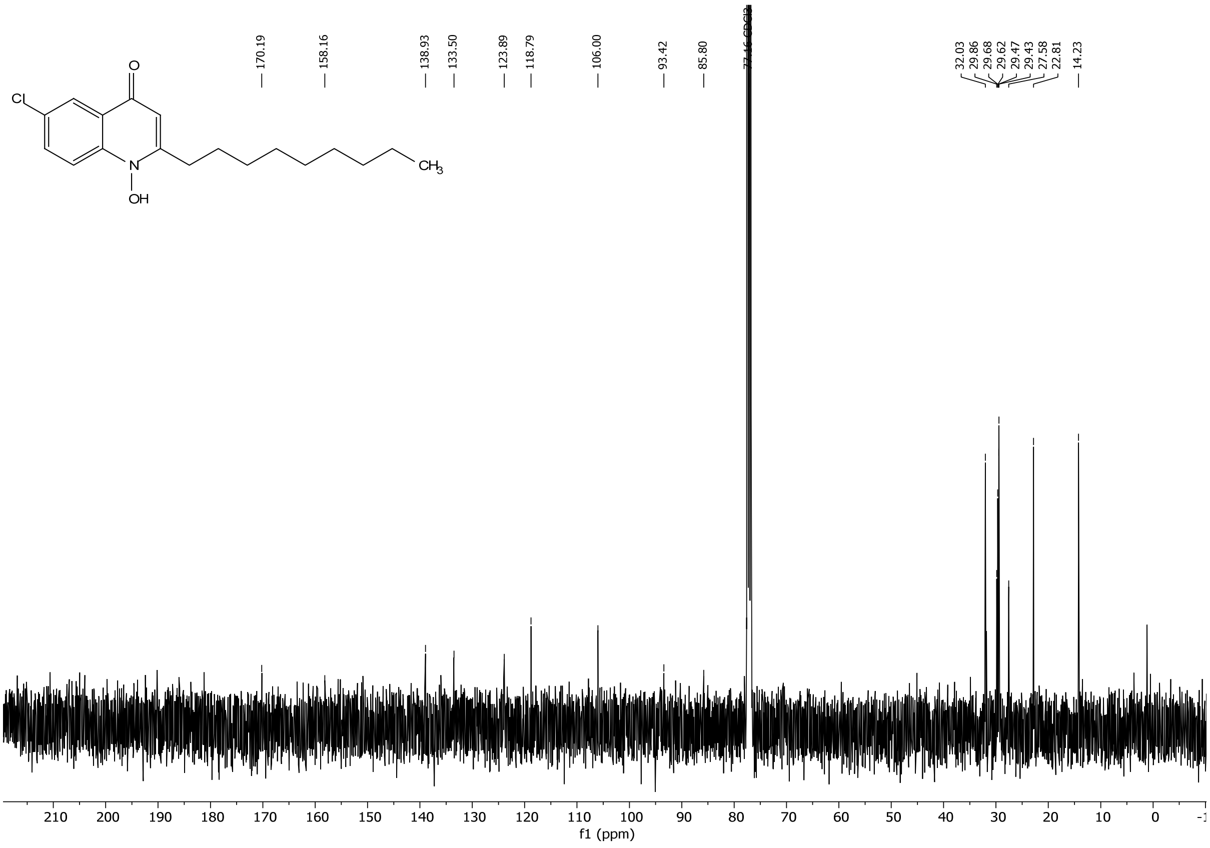

Figure SA17. ^{1}H-NMR and ^{13}C-NMR spectra of **6Cl-NQNO** in $CDCl_3$-*d*.

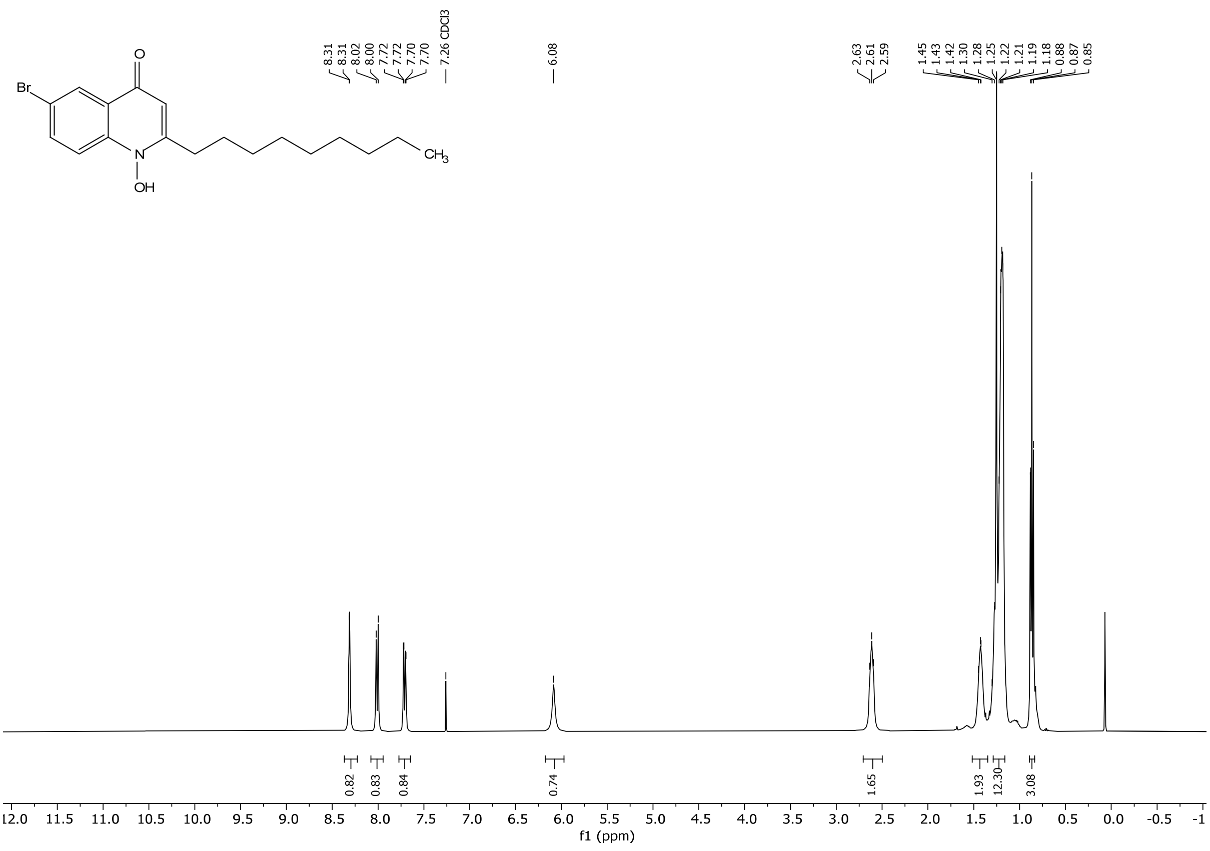

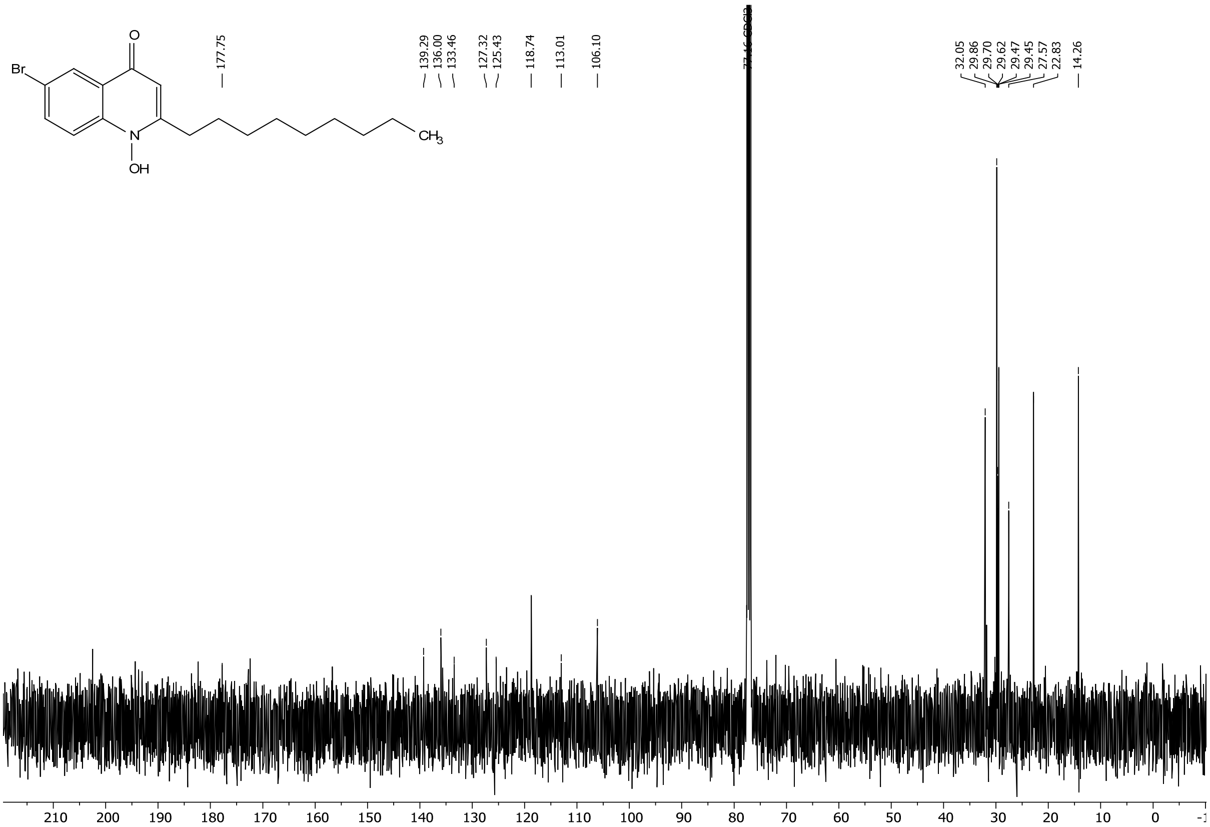

Figure SA18. ^{1}H-NMR and ^{13}C-NMR spectra of **6Br-NQNO** in $CDCl_3$-*d*.

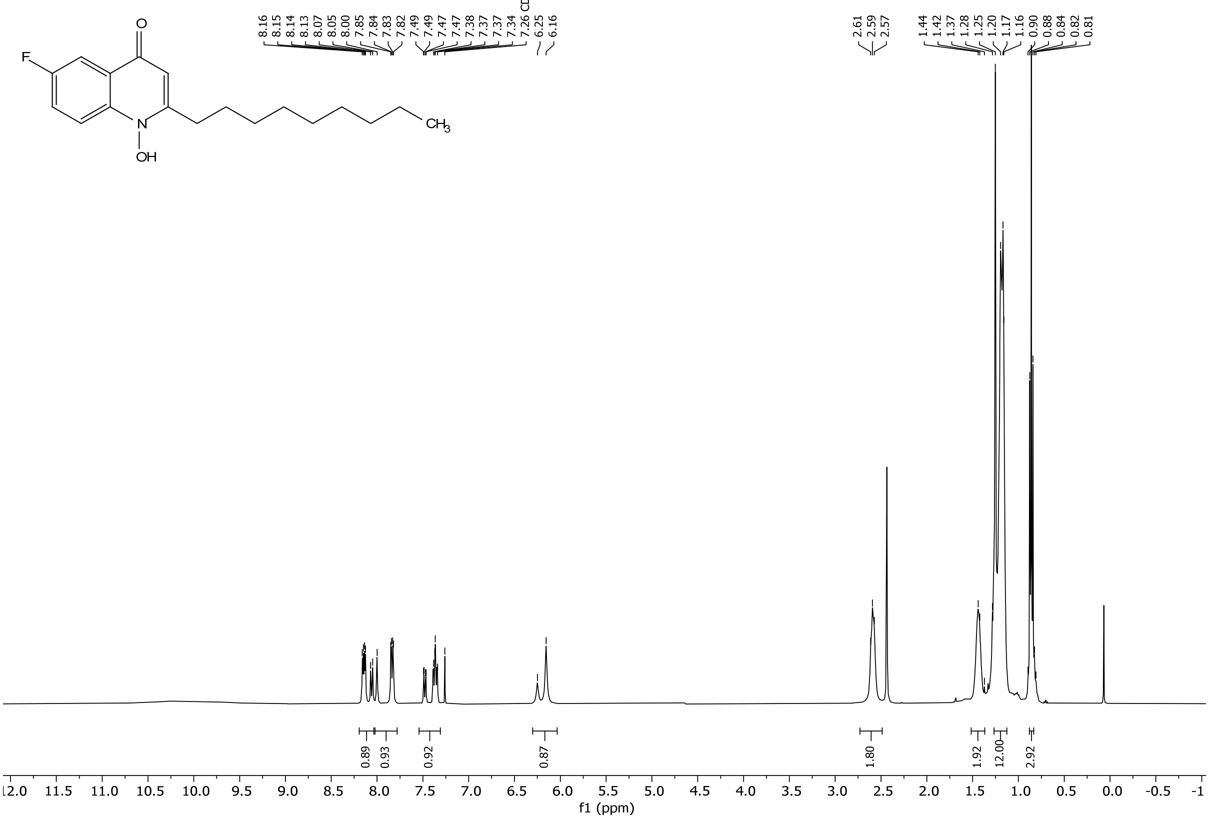

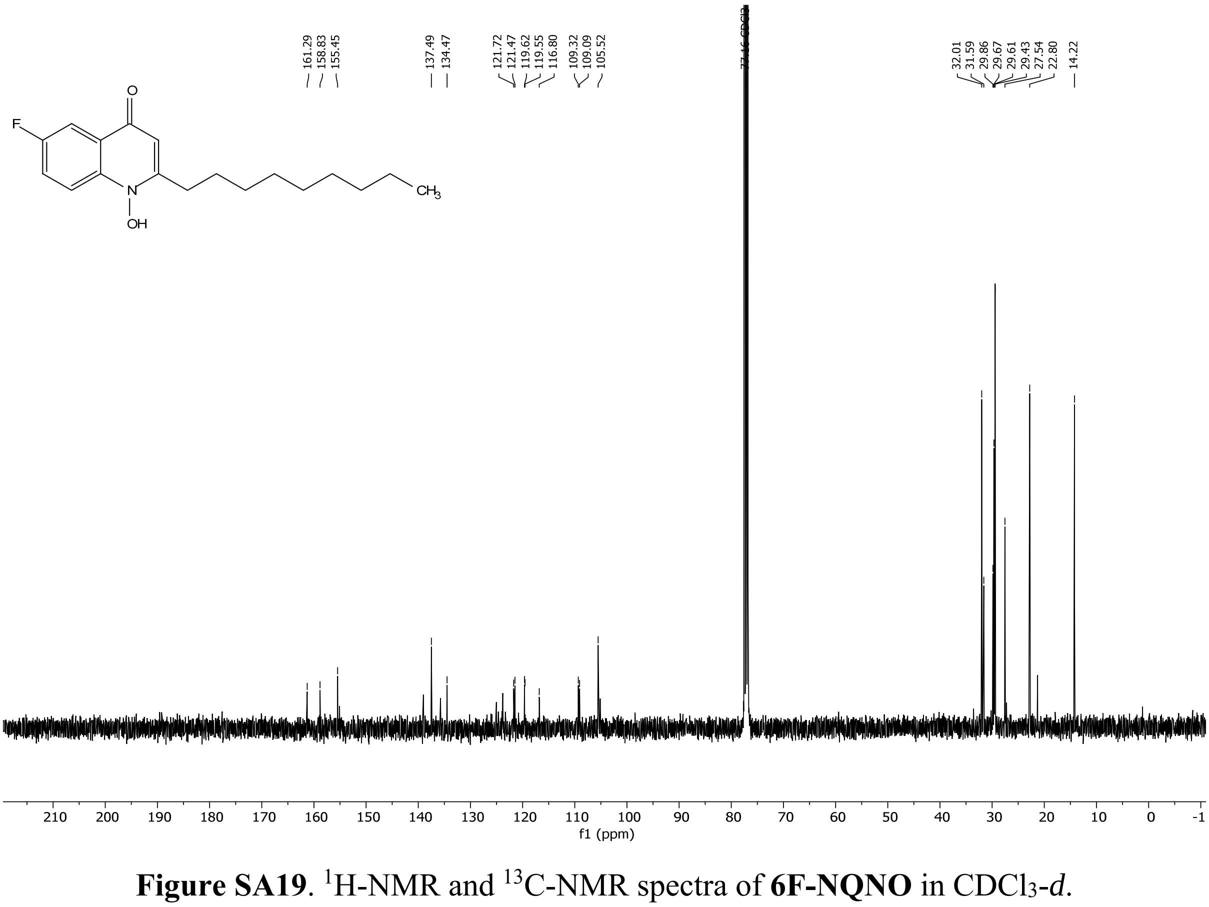

Figure SA19. ^{1}H-NMR and ^{13}C-NMR spectra of **6F-NQNO** in $CDCl_3$-*d*.

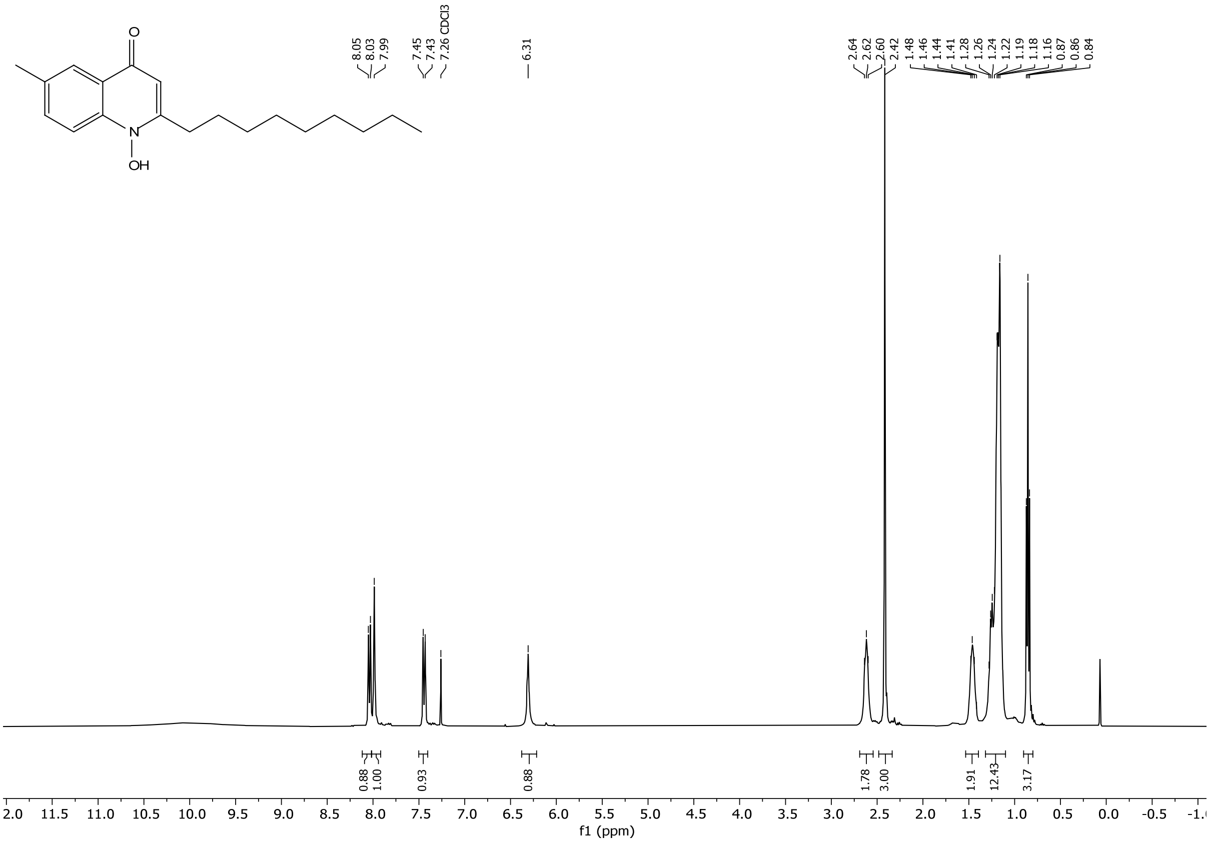

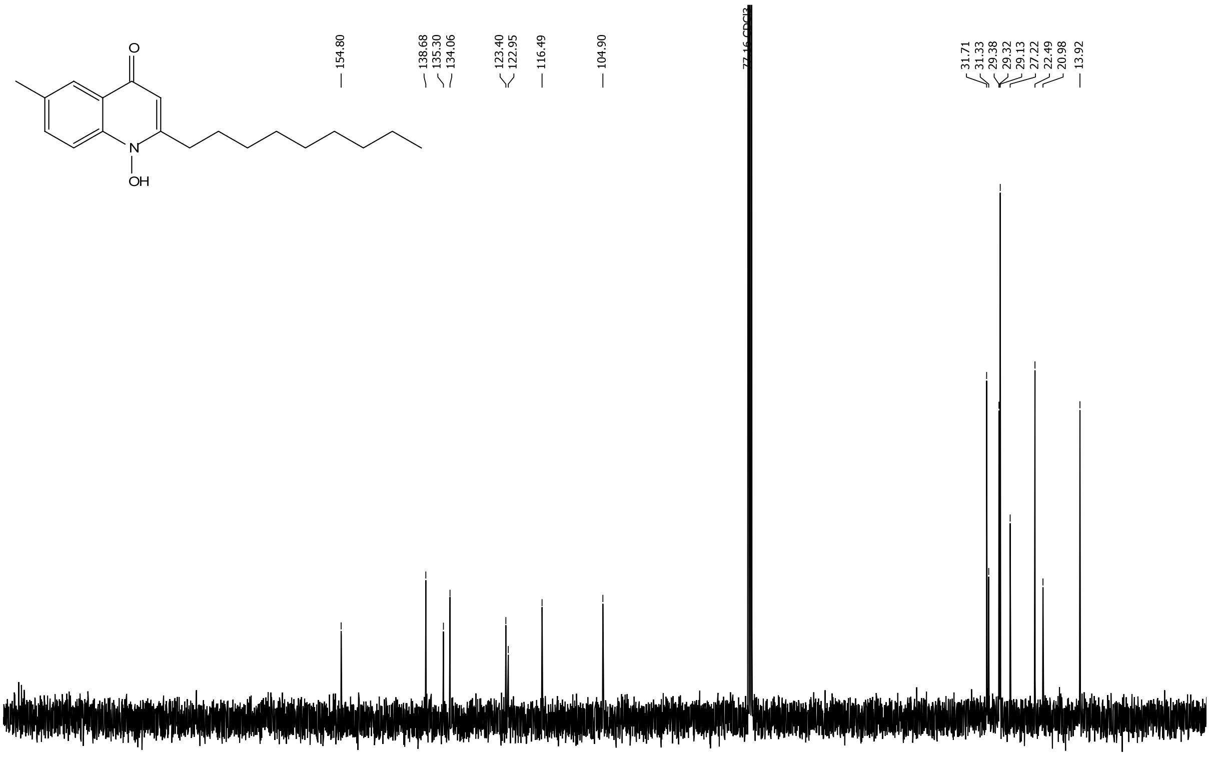

Figure SA20. ^{1}H-NMR and ^{13}C-NMR spectra of **6Me-NQNO** in $CDCl_3$-*d*.

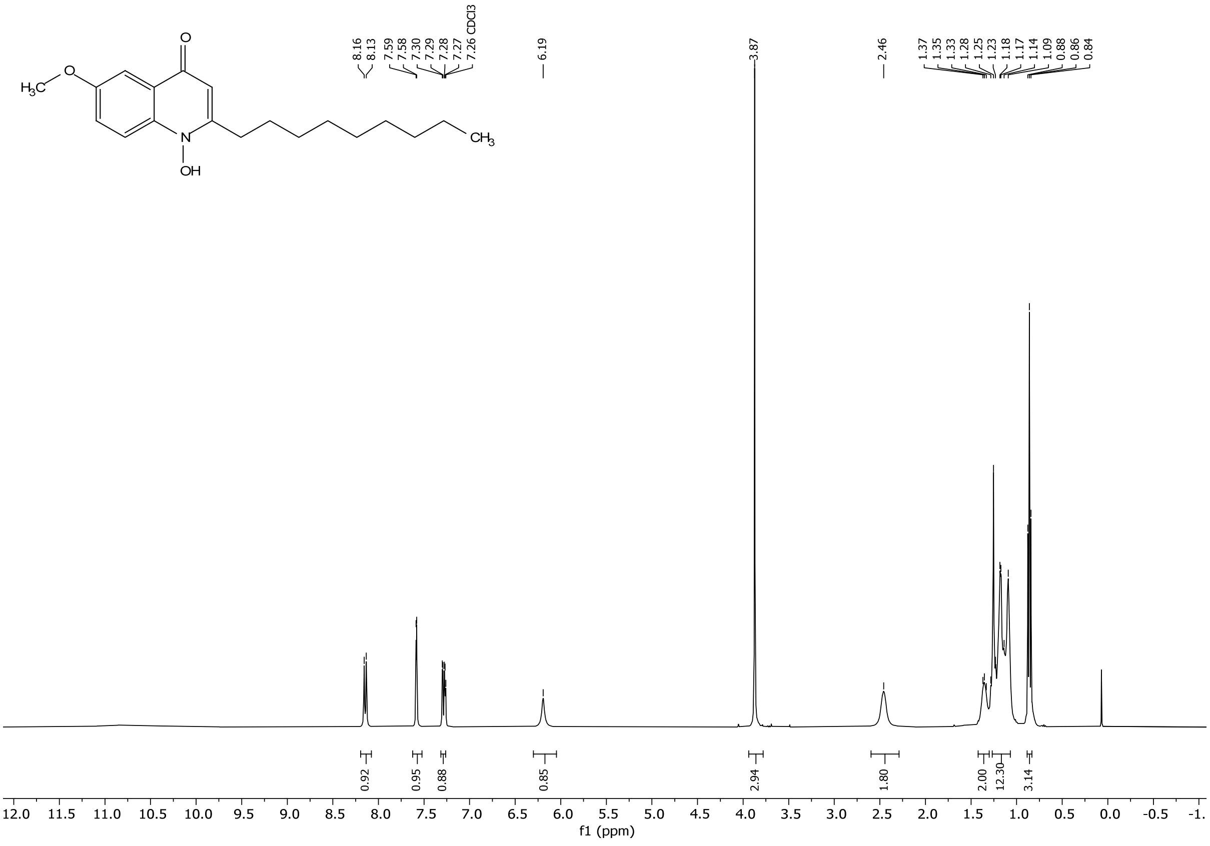

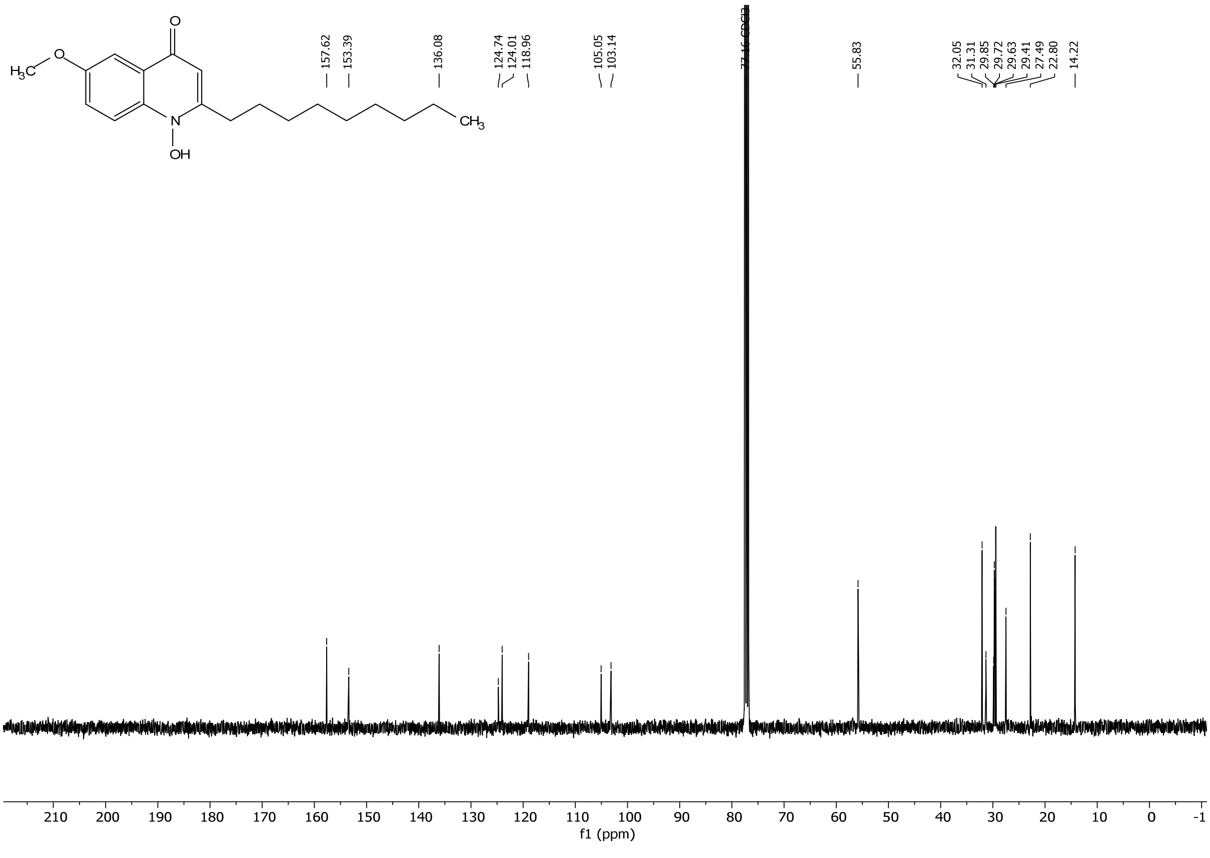

Figure SA21. ^{1}H-NMR and ^{13}C-NMR spectra of **6OMe-NQNO** in $CDCl_3$-*d*.

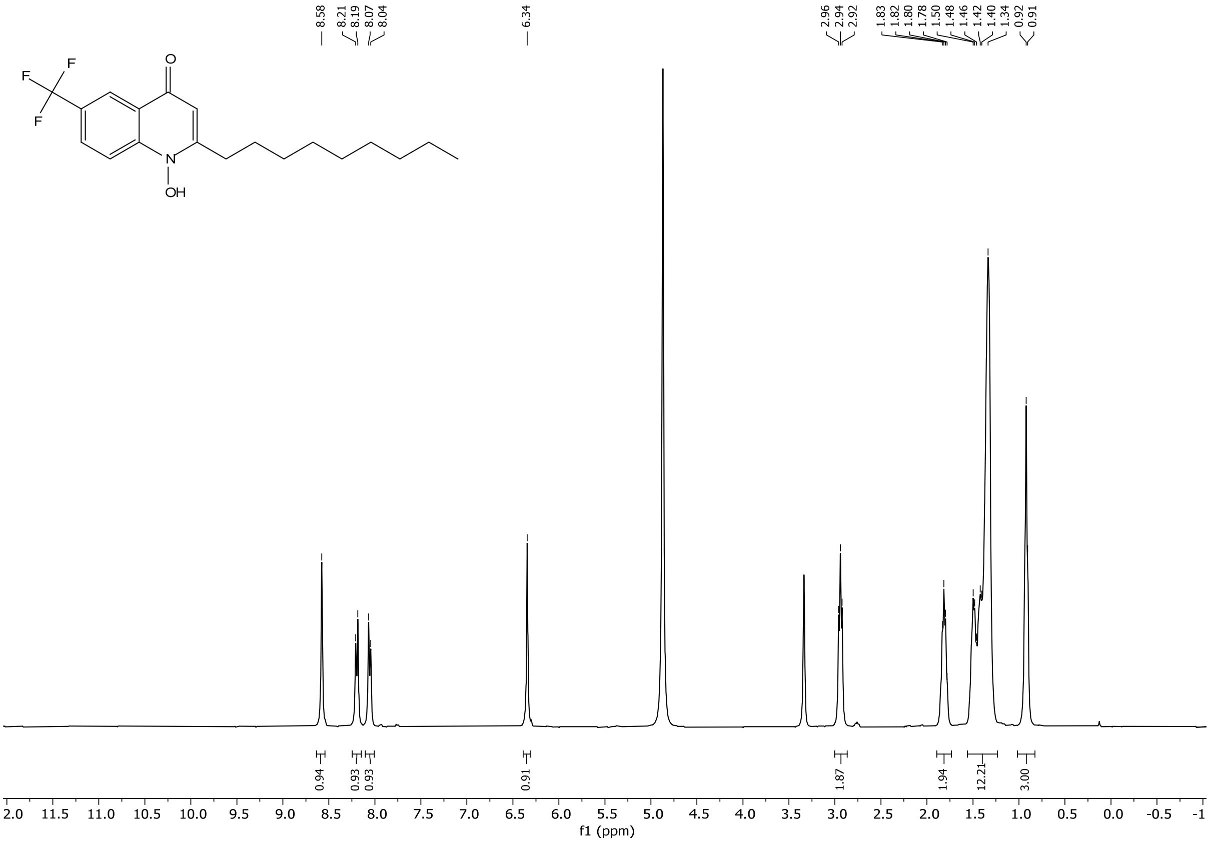

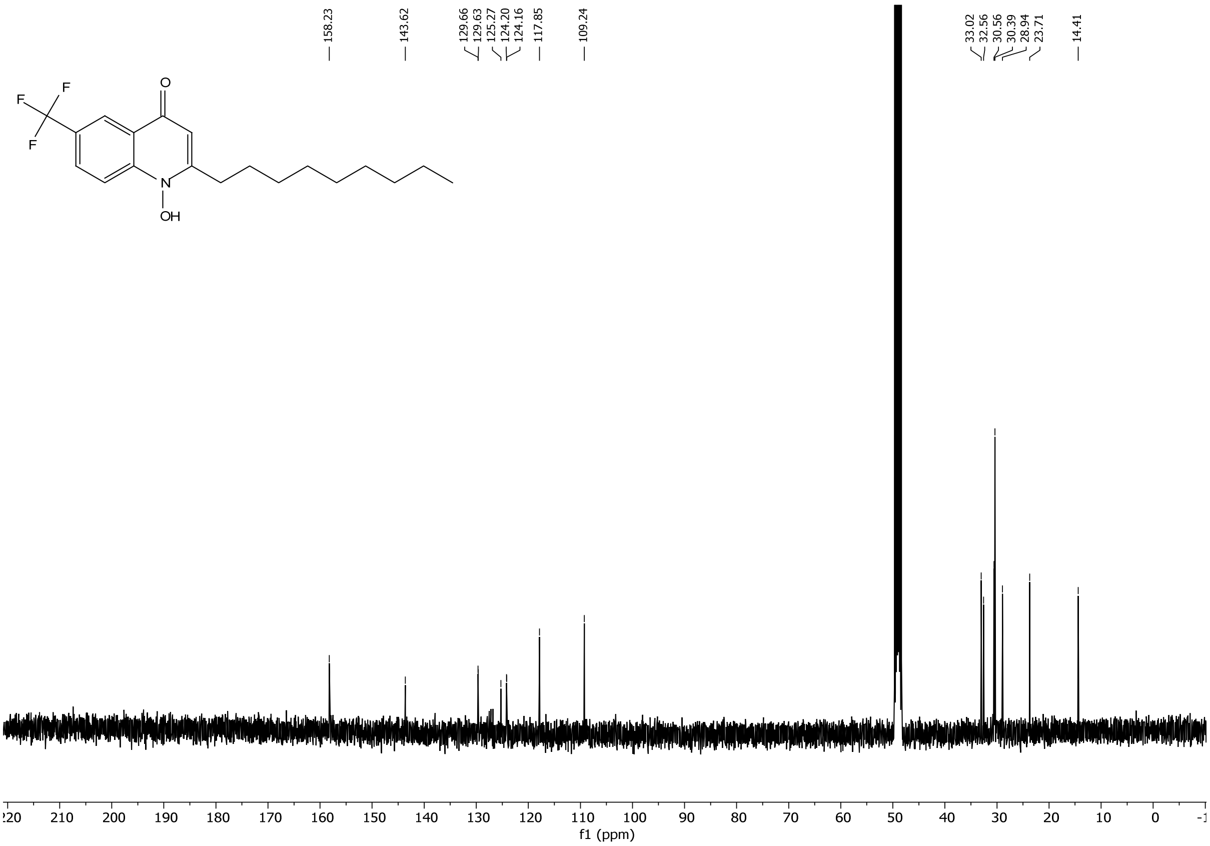

Figure SA22. ^{1}H-NMR and ^{13}C-NMR spectra of **$6CF_3$-NQNO** in MeOD-d_3.

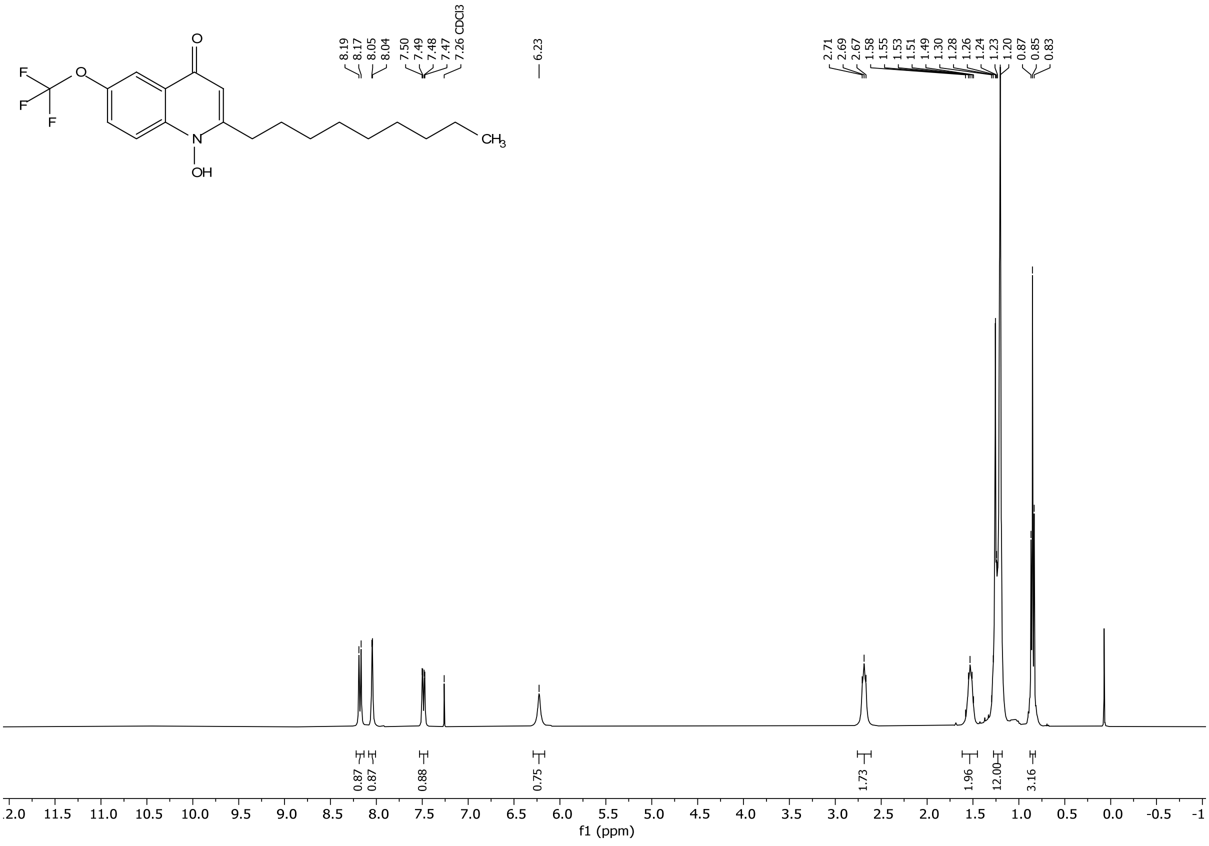

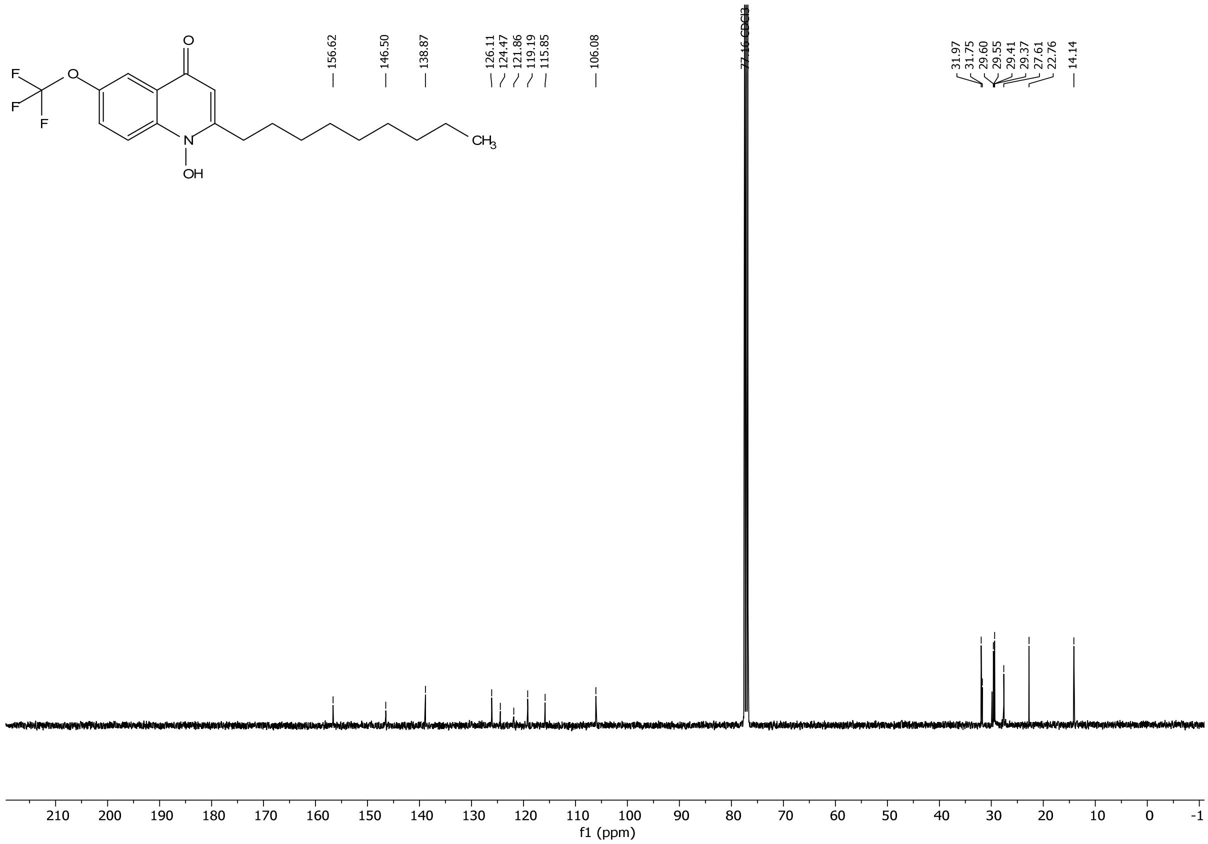

Figure SA23. ^{1}H-NMR and ^{13}C-NMR spectra of **6OCF$_3$-NQNO** in CDCl$_3$-*d*.

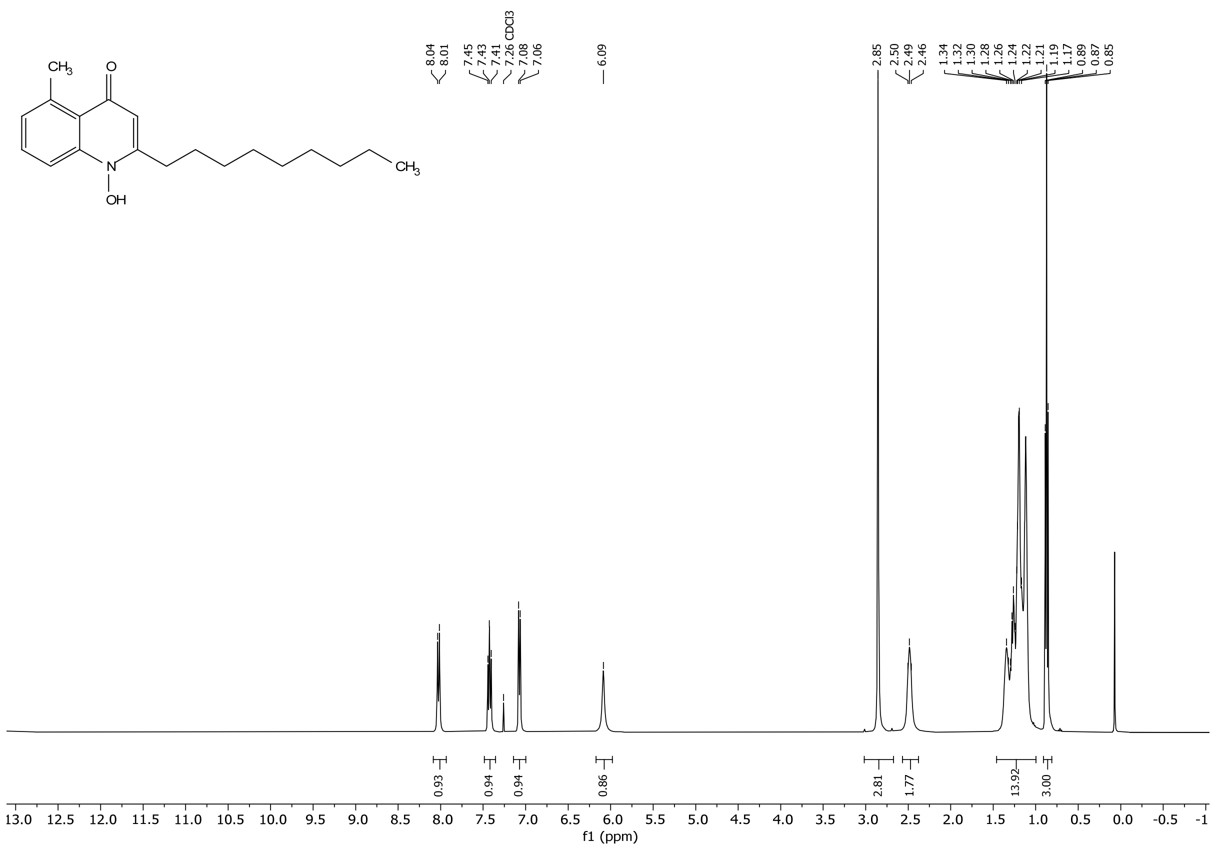

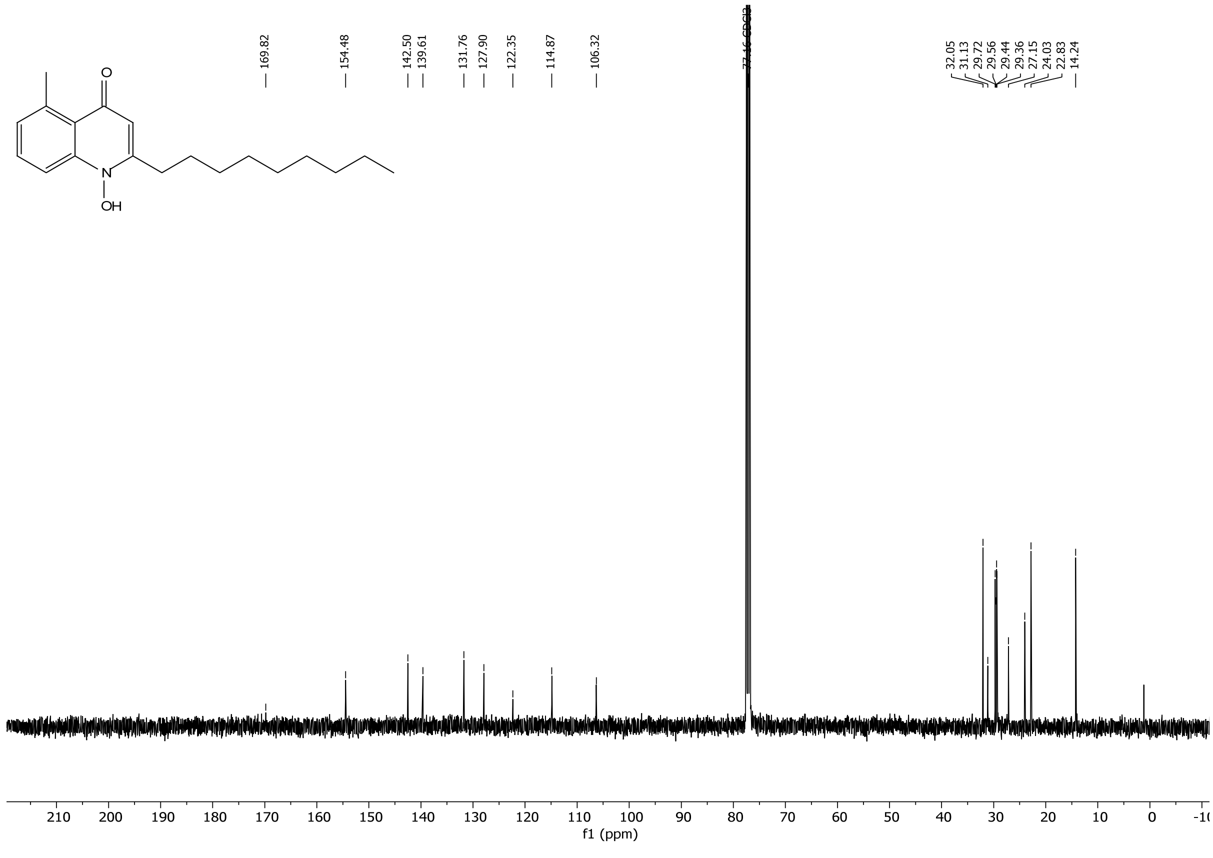

Figure SA24. ^{1}H-NMR and ^{13}C-NMR spectra of **5Me-NQNO** in $CDCl_3$-*d*.

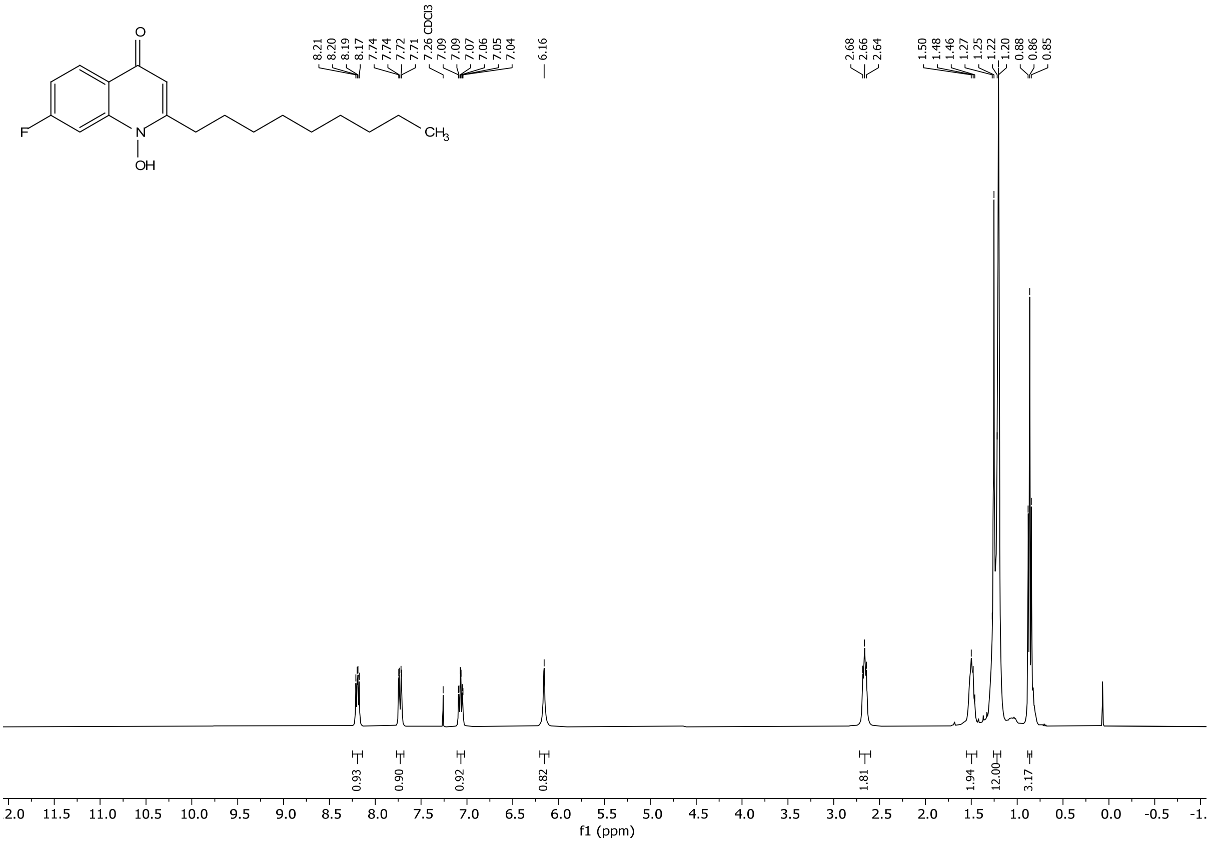

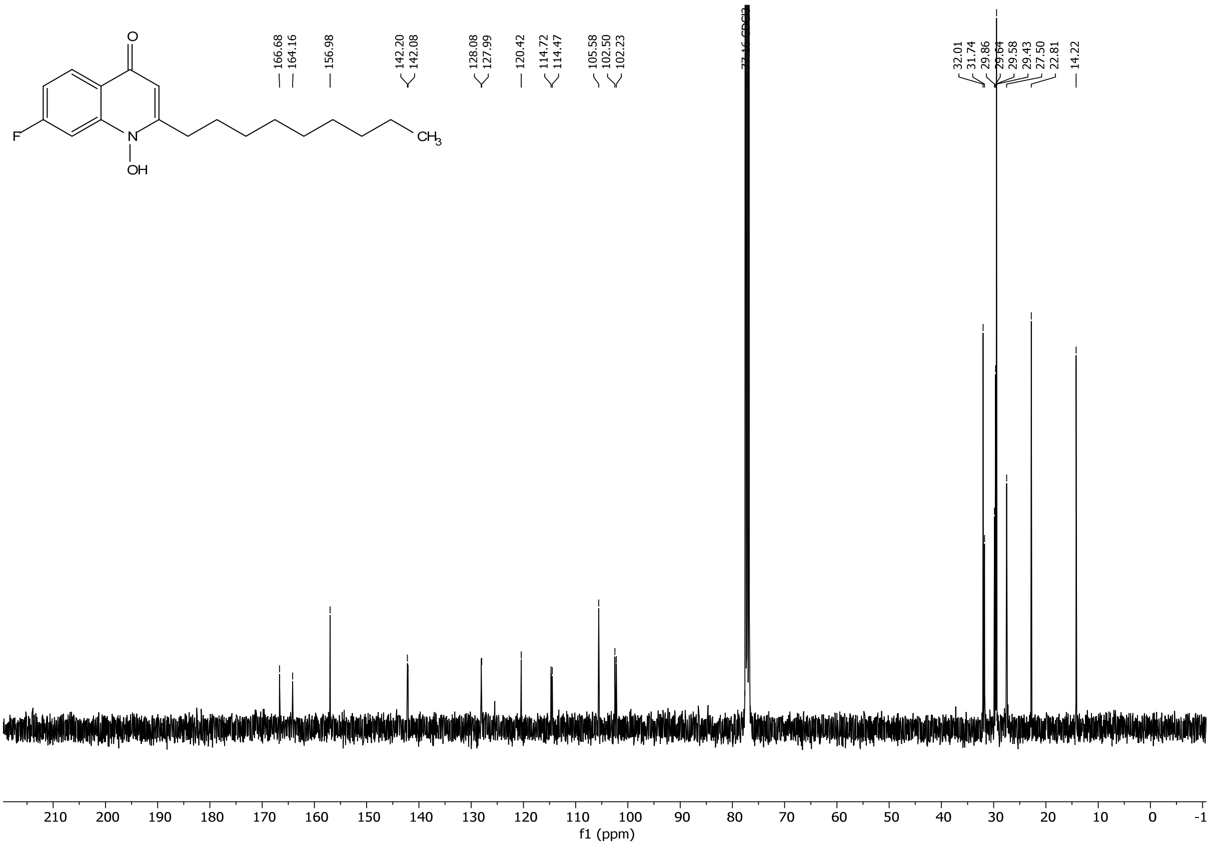

Figure SA25. ^{1}H-NMR and ^{13}C-NMR spectra of **7F-NQNO** in $CDCl_3$-*d*.

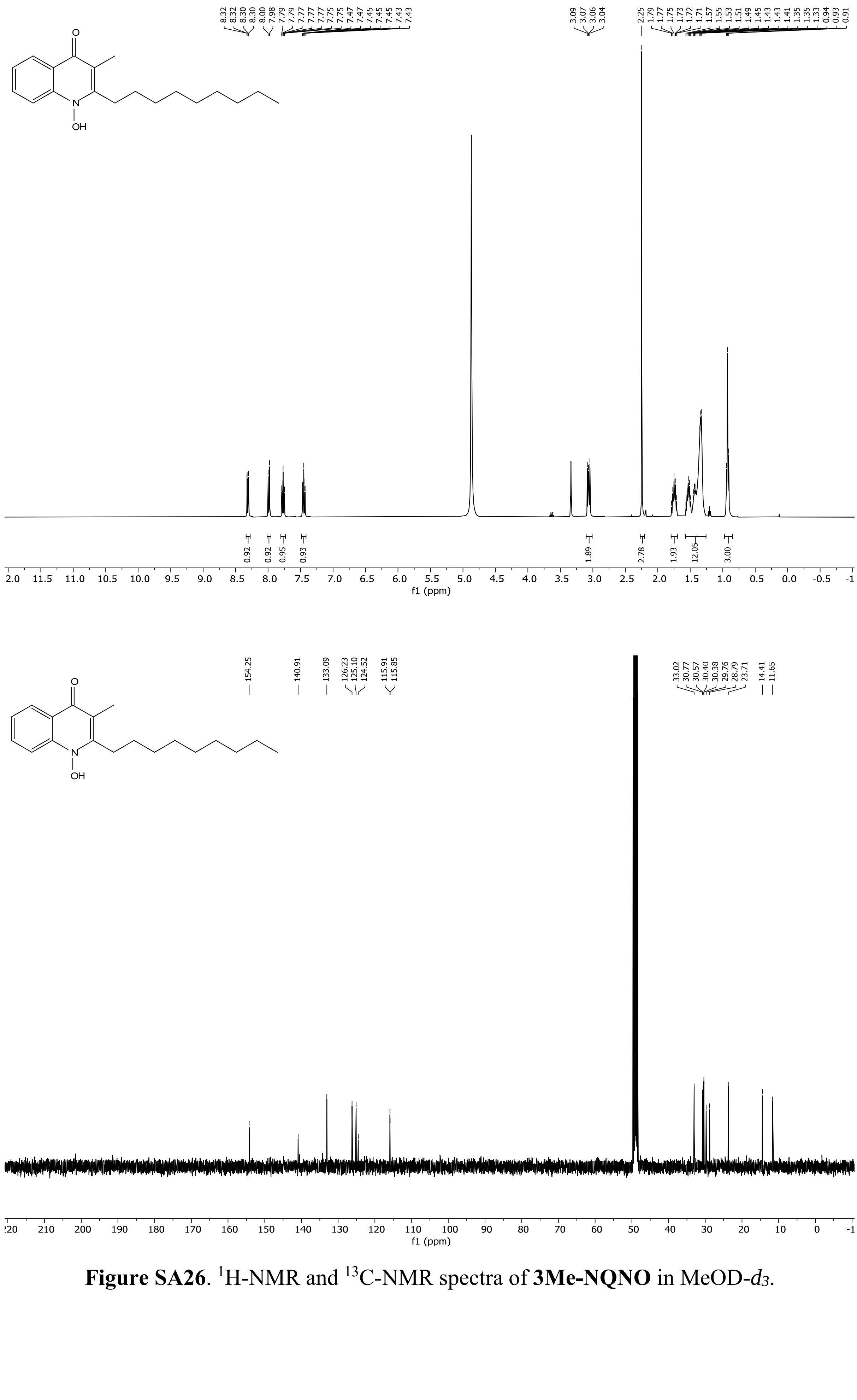

Figure SA26. ^{1}H-NMR and ^{13}C-NMR spectra of **3Me-NQNO** in MeOD-d_3.

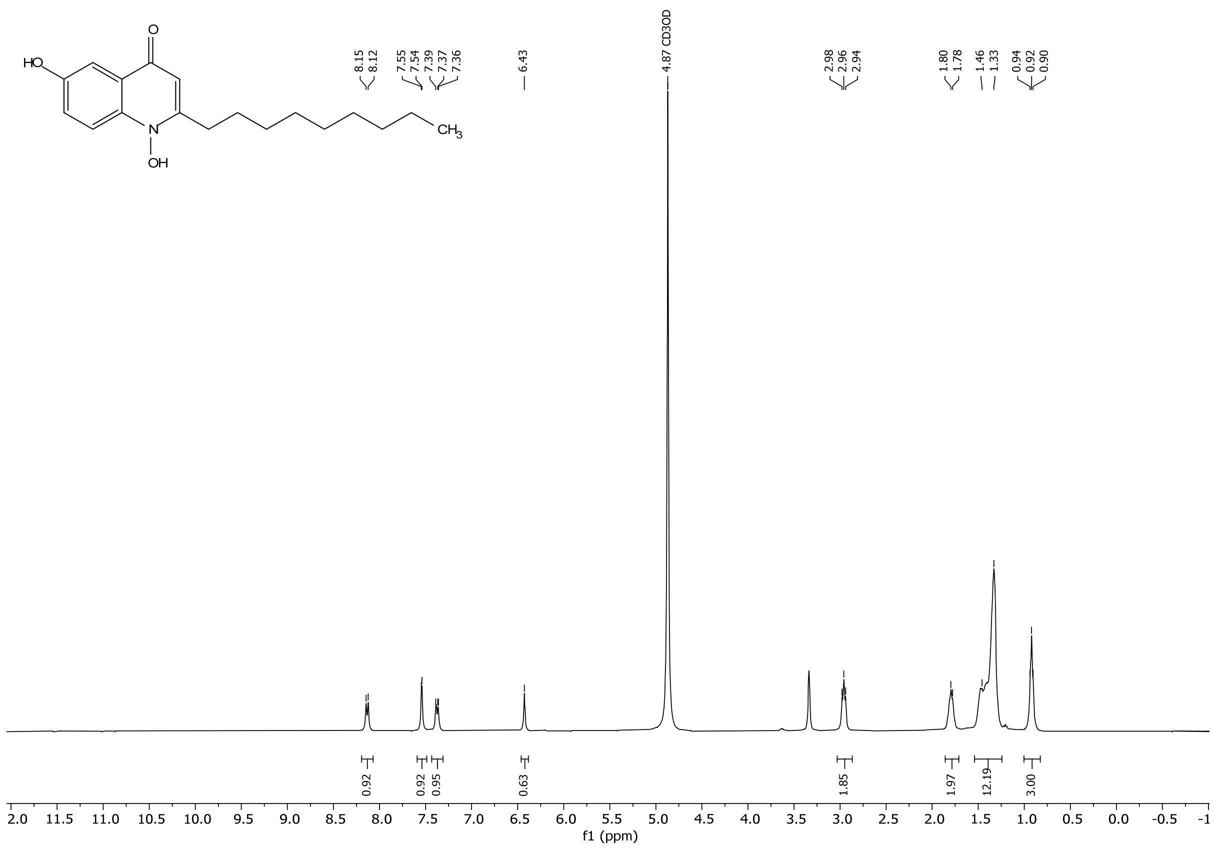

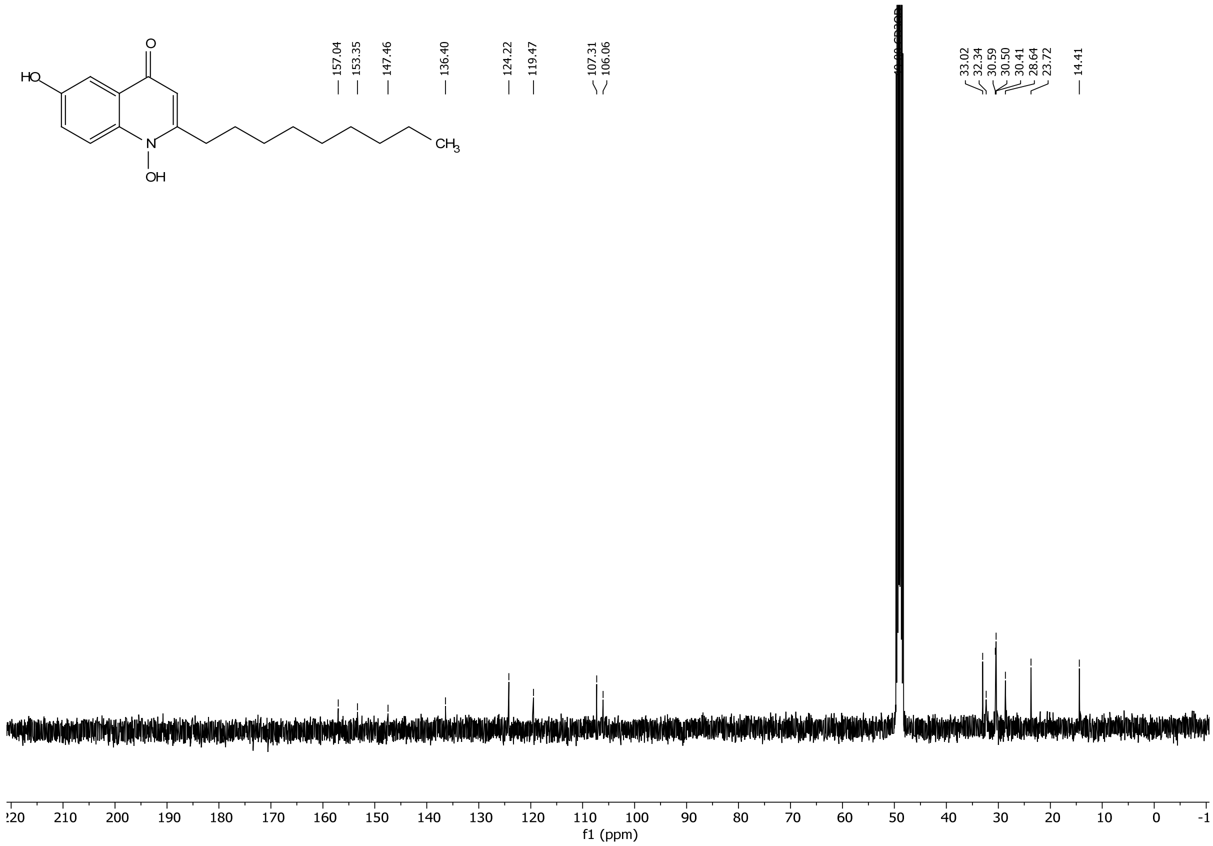

Figure SA27. ^{1}H-NMR and ^{13}C-NMR spectra of **6OH-NQNO** in MeOD-d_3.
