# Supplementary material for: A quinolone N-oxide antibiotic selectively targets Neisseria gonorrhoeae via its toxin–antitoxin system
Source: Nat Microbiol. 2025 Apr 2;10(4):939–57. doi: 10.1038/s41564-025-01968-y (PMC11964940; doi:10.1038/s41564-025-01968-y)
Supplement: Supplementary file 2 — Reporting Summary [file 41564_2025_1968_MOESM2_ESM.pdf]

nature portfolio

Corresponding author(s): Hauck & Böttcher

Last updated by author(s): 30-1-2023

Reporting Summary

Nature Portfolio wishes to improve the reproducibility of the work that we publish. This form provides structure for consistency and transparency in reporting. For further information on Nature Portfolio policies, see our Editorial Policies and the Editorial Policy Checklist.

Statistics

For all statistical analyses, confirm that the following items are present in the figure legend, table legend, main text, or Methods section.

n/a	Confirmed	
☐	☒	The exact sample size (*n*) for each experimental group/condition, given as a discrete number and unit of measurement
☐	☒	A statement on whether measurements were taken from distinct samples or whether the same sample was measured repeatedly
☐	☒	The statistical test(s) used AND whether they are one- or two-sided<br>*Only common tests should be described solely by name; describe more complex techniques in the Methods section.*
☒	☐	A description of all covariates tested
☐	☒	A description of any assumptions or corrections, such as tests of normality and adjustment for multiple comparisons
☐	☒	A full description of the statistical parameters including central tendency (e.g. means) or other basic estimates (e.g. regression coefficient) AND variation (e.g. standard deviation) or associated estimates of uncertainty (e.g. confidence intervals)
☐	☒	For null hypothesis testing, the test statistic (e.g. *F*, *t*, *r*) with confidence intervals, effect sizes, degrees of freedom and *P* value noted<br>*Give P values as exact values whenever suitable.*
☒	☐	For Bayesian analysis, information on the choice of priors and Markov chain Monte Carlo settings
☒	☐	For hierarchical and complex designs, identification of the appropriate level for tests and full reporting of outcomes
☐	☒	Estimates of effect sizes (e.g. Cohen's *d*, Pearson's *r*), indicating how they were calculated

Our web collection on statistics for biologists contains articles on many of the points above.

Software and code

Policy information about availability of computer code

Data collection	For gonococcal genome sequencing data obtained by the MiSeq sequencing system (Illumina), FastQC v0.11.5 (Andrews, S. (2010). FastQC: A Quality Control Tool for High Throughput Sequence Data. http://www.bioinformatics.babraham.ac.uk/projects/fastqc/) was used to analyze the quality of the raw sequencing data. The raw reads were trimmed by Sickle v1.33 (https://github.com/najoshi/sickle). SPAdes v3.10.1 (Bankevich, A., Nurk, S., Antipov, D., Gurevich, A.A., Dvorkin, M., Kulikov, A.S., Lesin, V.M., Nikolenko, S.I., Pham, S., Prjibelski, A.D., et al. (2012). SPAdes: a new genome assembly algorithm and its applications to single-cell sequencing. J Comput Biol 19, 455-477.) was used for the assembly of the raw reads.
Data analysis	For gene prediction and automatic annotation we employed Prokka (v1.12) (Seemann, T. (2014). Prokka: rapid prokaryotic genome annotation. Bioinformatics 30, 2068-2069.) and the NCBI Prokaryotic Genome Annotation Pipeline (PGAP release 5.2) (Tatusova, T., DiCuccio, M., Badretdin, A., Chetvernin, V., Nawrocki, E.P., Zaslavsky, L., Lomsadze, A., Pruitt, K.D., Borodovsky, M., and Ostell, J. (2016). NCBI prokaryotic genome annotation pipeline. Nucl Acids Res 44, 6614-6624.). For subsequent comparative genomic analysis, we used the PGAP annotation. The quality trimmed reads of the parent strain MS11 N309 and the two NQNO-resistant mutants MS11-R1 and MS11-R2 were aligned to the reference genome of N. gonorrhoeae MS11 (accession number NC_022240; https://www.ncbi.nlm.nih.gov/nuccore/NC_022240.1/) with the BWA-MEM algorithm from the Burrows-Wheeler Aligner (BWA) software package (v0.7.17) (Li, H., and Durbin, R. (2010). Fast and accurate long-read alignment with Burrows-Wheeler transform. Bioinformatics 26, 589-595). The produced alignment was sorted with SortSam, and PCR duplicates were marked with MarkDuplicates using Picard (v2.17.3) (http://broadinstitute.github.io/picard/), for downstream analysis.<br>The Genome Analysis Toolkit's (GATK) (v3.8.0) RealignerTargetCreator and IndelRealigner tools were used to perform realignment around the Indels (McKenna, A., Hanna, M., Banks, E., Sivachenko, A., Cibulskis, K., Kernytsky, A., Garimella, K., Altshuler, D., Gabriel, S., Daly, M., et al. (2010). The Genome Analysis Toolkit: a MapReduce framework for analyzing next-generation DNA sequencing data. Genome Res 20, 1297-1303.). The GATK BaseRecalibrator was used to perform base quality score recalibration. Variant calling was then performed using the

GATK HaplotypeCaller with the default parameters except for the ploidy that was set to 1, according to GATK Best Practices. The sorted SNPs and InDels were then filtered using the GATK VariantFilteration with the filtering criteria recommended by GATK. To further remove sequencing bias only variants with a minimum read depth of 10 were considered. The effects of the variants were then annotated using SnpEff (v4.3s) (Cingolani, P., Platts, A., Wang le, L., Coon, M., Nguyen, T., Wang, L., Land, S.J., Lu, X., and Ruden, D.M. (2012). A program for annotating and predicting the effects of single nucleotide polymorphisms, SnpEff: SNPs in the genome of Drosophila melanogaster strain w1118; iso-2; iso-3. Fly (Austin) 6, 80-92).

For manuscripts utilizing custom algorithms or software that are central to the research but not yet described in published literature, software must be made available to editors and reviewers. We strongly encourage code deposition in a community repository (e.g. GitHub). See the Nature Portfolio guidelines for submitting code & software for further information.

Data

Policy information about availability of data

All manuscripts must include a data availability statement. This statement should provide the following information, where applicable:

- Accession codes, unique identifiers, or web links for publicly available datasets
- A description of any restrictions on data availability
- For clinical datasets or third party data, please ensure that the statement adheres to our policy

The draft genome sequences of the three strains MS11_N309 (accession no. SAMN19108268), MS11-R1 (N568) (accession no. SAMN19108269) and MS11-R2 (N569) (accession no. SAMN19108270) were combined in Bioproject number PRJNA728975 and are publicly available from the NCBI GenBank. The accessible links are:
N. gonorrhoeae MS11-N309: https://www.ncbi.nlm.nih.gov/nuccore/JAHBBP000000000
N. gonorrhoeae MS11-R1: https://www.ncbi.nlm.nih.gov/nuccore/JAHBBO000000000
N. gonorrhoeae MS11-R2: https://www.ncbi.nlm.nih.gov/nuccore/JAHBBN000000000

As a reference genome, the publicly available genome of N. gonorrhoeae MS11 was used (accession number NC_022240; https://www.ncbi.nlm.nih.gov/nuccore/NC_022240.1/)
All other primary data are contained within the manuscript, including the extended supplementary information about chemical synthesis of all compounds and their quality control.

Human research participants

Policy information about studies involving human research participants and Sex and Gender in Research.

Reporting on sex and gender	*Use the terms sex (biological attribute) and gender (shaped by social and cultural circumstances) carefully in order to avoid confusing both terms. Indicate if findings apply to only one sex or gender; describe whether sex and gender were considered in study design whether sex and/or gender was determined based on self-reporting or assigned and methods used. Provide in the source data disaggregated sex and gender data where this information has been collected, and consent has been obtained for sharing of individual-level data; provide overall numbers in this Reporting Summary. Please state if this information has not been collected. Report sex- and gender-based analyses where performed, justify reasons for lack of sex- and gender-based analysis.*
Population characteristics	*Describe the covariate-relevant population characteristics of the human research participants (e.g. age, genotypic information, past and current diagnosis and treatment categories). If you filled out the behavioural & social sciences study design questions and have nothing to add here, write "See above."*
Recruitment	*Describe how participants were recruited. Outline any potential self-selection bias or other biases that may be present and how these are likely to impact results.*
Ethics oversight	*Identify the organization(s) that approved the study protocol.*

Note that full information on the approval of the study protocol must also be provided in the manuscript.

Field-specific reporting

Please select the one below that is the best fit for your research. If you are not sure, read the appropriate sections before making your selection.

☒ Life sciences ☐ Behavioural & social sciences ☐ Ecological, evolutionary & environmental sciences

For a reference copy of the document with all sections, see nature.com/documents/nr-reporting-summary-flat.pdf

Life sciences study design

All studies must disclose on these points even when the disclosure is negative.

Sample size	Based on initial experiments with the first generation of NQNO compounds, we calculated the effect size (Cohen's d) on recovered viable bacteria from NQNO-treated versus control (DMSO)-treated animals to be between 2 and 3. Based on an effect size of 3, we have performed

an a priori power analysis with a minimum probability for the type I-error of $p<0.05$ (the probability that we reject H0 despite it is correct) to predict, how large a sample size we need to achieve this significance level. The a priori analysis indicated that the sample size should consist of at least 4 animals per group.

Data exclusions	No data were excluded
Replication	All experiments contained technical and biological replicates as detailed in Material&Methods and Figure legends.
Randomization	Animals were assigned randomly to the treatment or control group by drawing labeled cards from a blinded container
Blinding	Data collection and analysis were performed by experimentators, who were not blind to the conditions of the experiments.

Reporting for specific materials, systems and methods

We require information from authors about some types of materials, experimental systems and methods used in many studies. Here, indicate whether each material, system or method listed is relevant to your study. If you are not sure if a list item applies to your research, read the appropriate section before selecting a response.

Materials & experimental systems

n/a	Involved in the study	
☐	☒	Antibodies
☐	☒	Eukaryotic cell lines
☒	☐	Palaeontology and archaeology
☐	☒	Animals and other organisms
☒	☐	Clinical data
☒	☐	Dual use research of concern

Methods

n/a	Involved in the study	
☒	☐	ChIP-seq
☒	☐	Flow cytometry
☒	☐	MRI-based neuroimaging

Antibodies

Antibodies used	The following primary and secondary antibodies were employed at the indicated dilutions for Western blotting (WB). Monoclonal antibody (mAB) against 6x His (clone H8; Thermo Fisher Scientific; 1:1.000 WB); mAb against GAPDH (clone GA1R, Invitrogen, 1:2.000 WB); mAb against GFP (clone JL-8, Clontech, Palo Alto, CA, 1:5000); mAb α-Opa (clone 4B12/C11, Developmental Studies Hybridoma Bank, University of Iowa, USA, a generous gift of M. Achtman, WB: 1:2000). Rabbit polyclonal antibody (pRb) against the synthetic epsilon1 peptide C-EKNRRMMTDEAFRKEVEKRLYAG was produced by PSL GmbH (Heidelberg, Germany), affinity purified against the cognate peptide and used at 1µg/ml for WB; pRb against the synthetic zeta1 peptides AKKEYSKQRVVTNSK-C and KIVGINQDRNSEFIDK-C was produced by PSL GmbH (Heidelberg, Germany) and affinity purified using both peptides (1µg/ml pRb was used for WB). HRP-conjugated goat anti-mouse IgG (cat-no: 115-035-146; 1:10.000 WB) and HRP-conjugated goat anti-rabbit IgG (cat-no: 111-035-003; 1:5.000 WB) were from Jackson Immunoresearch (West Grove, PA).
Validation	The polyclonal antisera against epsilon1 and zeta1 were affinity purified against the synthetic peptides used to raise the antibodies.<br>For validation, the polyclonal antibodies against epsilon1/zeta1 were used in Western Blotting assays with bacterial lysates obtained from gonococcal strains lacking the toxin/antitoxin system (negative control) or expressing the toxin/antitoxin. The validation data are part of the manuscript.<br>For validation, the monoclonal antibodies against Opa proteins were used in Western Blotting assays with bacterial lysates obtained from gonococcal strains lacking Opa expression (negative control) or expressing a panel of different Opa proteins. The validation data have been published as part of a previous study (Roth A, Mattheis C, Muenzner P, Unemo M, Hauck CR. Innate recognition by neutrophil granulocytes differs between Neisseria gonorrhoeae strains causing local or disseminating infections. Infect Immun. 2013 Jul;81(7):2358-70. doi: 10.1128/IAI.00128-13).<br>The commercially available monoclonal antibodies have been validated by the suppliers.

Eukaryotic cell lines

Policy information about cell lines and Sex and Gender in Research

Cell line source(s)	Primary human vaginal epithelial cells (hVEC line MS74) were obtained from A.J. Schaeffer (Feinberg School of Medicine, Northwestern University, Chicago, IL) and are derived from vaginal tissue of a post-menopausal woman. The cell line was created through immortalization of the cells with human papilloma virus 16, E6 and E7 genes according to (Rajan, N., Pruden, D.L., Kaznari, H., Cao, Q., Anderson, B.E., Duncan, J.L., and Schaeffer, A.J. (2000). Characterization of an immortalized human vaginal epithelial cell line. J Urol 163, 616-622).<br>HeLa S3 cells were obtained from DSMZ, Braunschweig, Germany (ACC 161) and ME-180 cells were obtained from ATCC, Rockville, MD (ATCC HTB-33)
Authentication	The authentication was performed at the source by immunostaining for cytokeratins (hVEC) or STR analysis according to the global standard ANSI/ATCC ASN-0002.1-2021 (HeLa S3 and ME-180)

Mycoplasma contamination	All eukaryotic cell lines were screened in-house by a PCR-test for the presence of mycoplasma (Uphoff CC, Drexler HG (2005) Detection of mycoplasma contaminations. Methods Mol Biol 290: 13-23) and were tested to be Mycoplasma free.
Commonly misidentified lines (See ICLAC register)	no commonly misidentified cell lines were used.

Animals and other research organisms

Policy information about studies involving animals; ARRIVE guidelines recommended for reporting animal research, and Sex and Gender in Research

Laboratory animals	C57BL/6J mice carrying the complete human CEA gene (CEAtg mice) (Eades-Perner, A.M., van der Putten, H., Hirth, A., Thompson, J., Neumaier, M., von Kleist, S., and Zimmermann, W. (1994). Mice transgenic for the human carcinoembryonic antigen gene maintain its spatiotemporal expression pattern. Cancer Res. 54, 4169–4176.) and wildtype C57BL/6J mice (obtained from Elevage Janvier, Le Genest Saint Isle, France) were kept under specified pathogen-free conditions under a 12-h light cycle in the animal facility of University of Konstanz in accordance with national and institutional guidelines. Only female mice between 6 – 8 weeks of age were used.
Wild animals	The study did not involve wild animals
Reporting on sex	Only female mice were used, as in vivo infections were studied in a vaginal infection model.
Field-collected samples	The study did not involve samples collected from the field
Ethics oversight	Experiments involving animals were performed in accordance with the German Law for the Protection of Animal Welfare. The animal care and use protocol, including the protocol of experimental vaginal infection of female mice, was approved by the appropriate state ethics committee and state authorities regulating animal experiments (Regierungspraesidium Freiburg, Germany) under the permit file number G-19/147.

Note that full information on the approval of the study protocol must also be provided in the manuscript.
