# Supplementary material for: A quinolone N-oxide antibiotic selectively targets Neisseria gonorrhoeae via its toxin–antitoxin system
Source: Nat Microbiol. 2025 Apr 2;10(4):939–57. doi: 10.1038/s41564-025-01968-y (PMC11964940; doi:10.1038/s41564-025-01968-y)
Supplement: Supplementary file 6 — Unprocessed, labelled agarose gels (a,d) and western blots (g). [file 41564_2025_1968_MOESM6_ESM.pdf]

Blots zu Figure 3

A

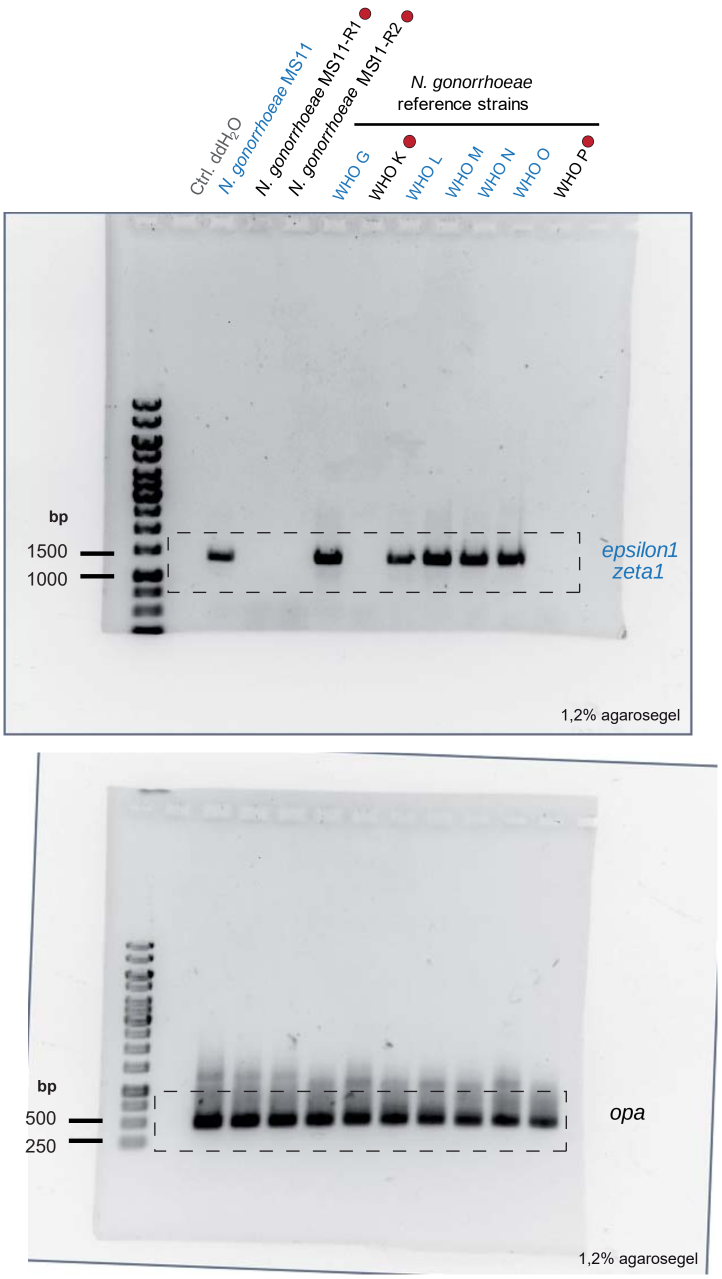

D

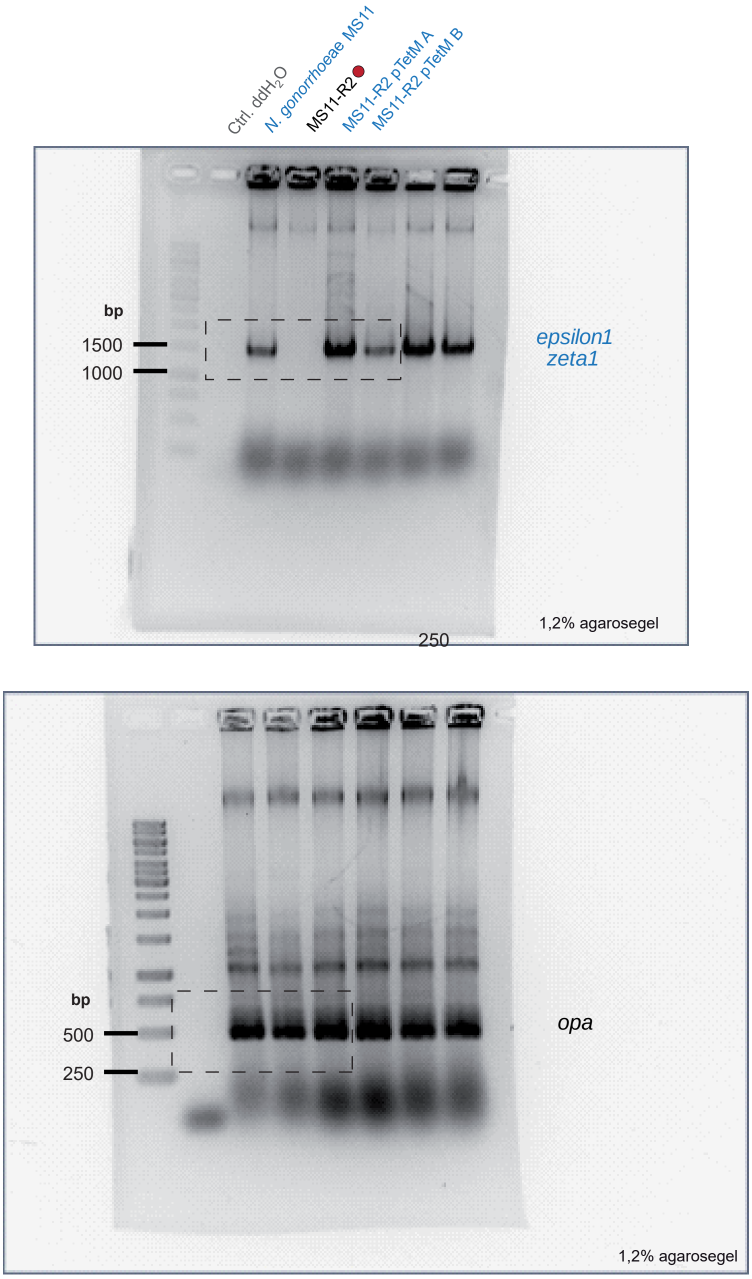

Blots zu Figure 3

G

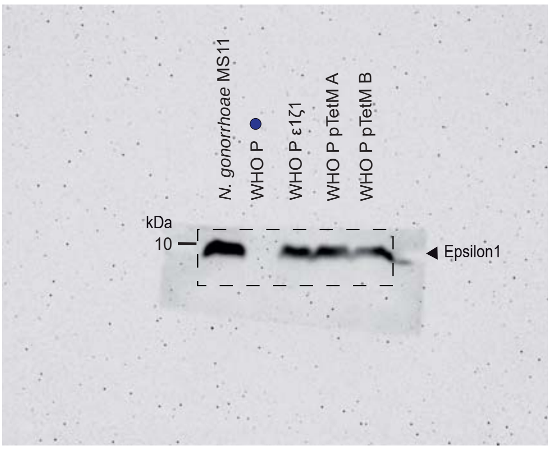

WCL, anti-Epsilon1

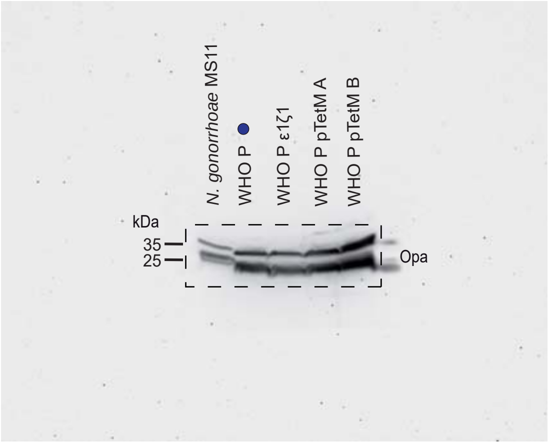

WCL, anti-Opa

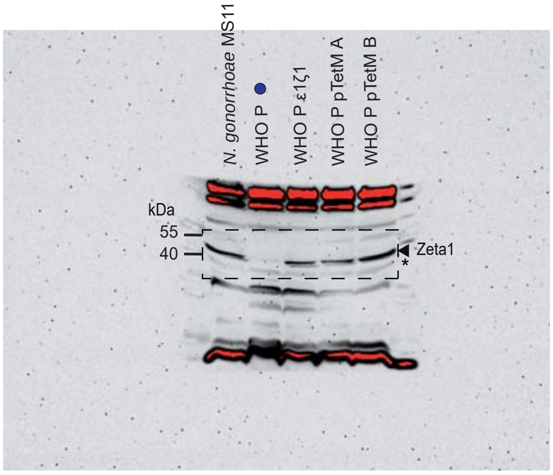

WCL, anti-Zeta1

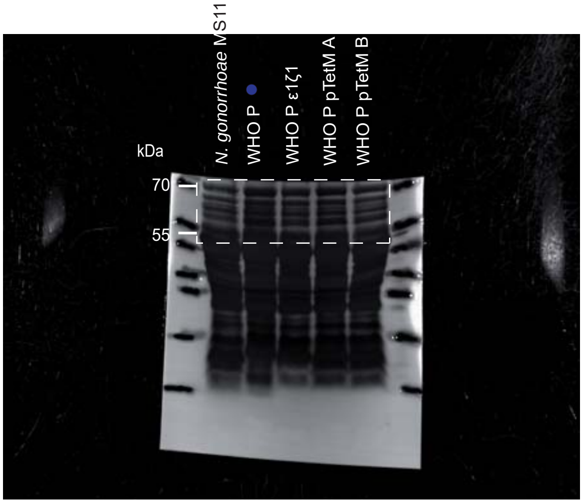

Coomassie

● = high-level resistant (50 µM NQNO)
