# Supplementary material for: A quinolone N-oxide antibiotic selectively targets Neisseria gonorrhoeae via its toxin–antitoxin system
Source: Nat Microbiol. 2025 Apr 2;10(4):939–57. doi: 10.1038/s41564-025-01968-y (PMC11964940; doi:10.1038/s41564-025-01968-y)
Supplement: Supplementary file 7 — Unprocessed, labelled western blots. [file 41564_2025_1968_MOESM7_ESM.pdf]

Blots zu Figure 4

A

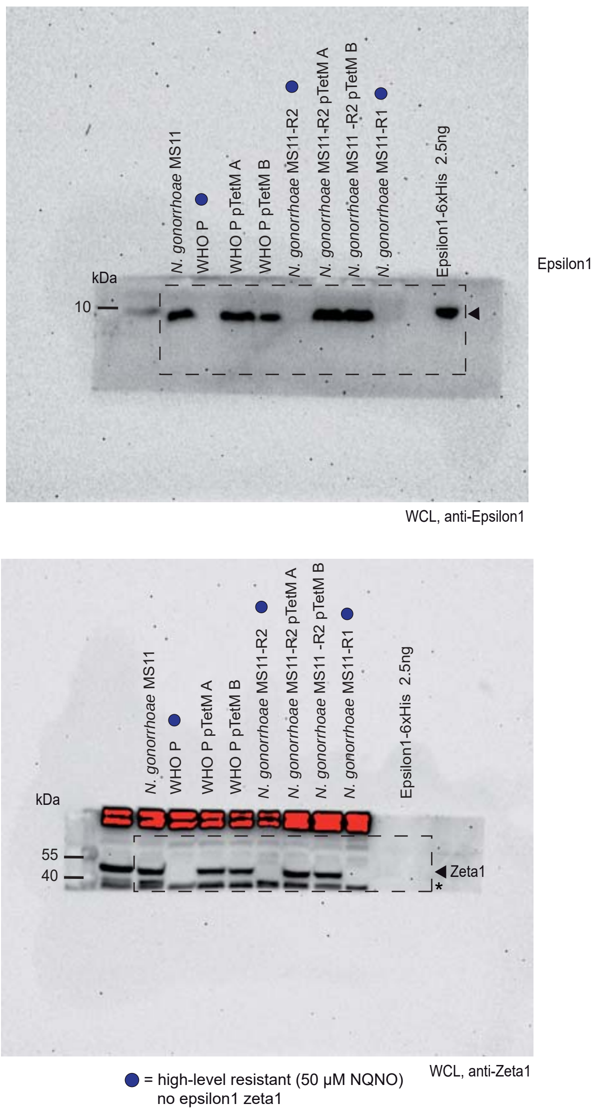

B

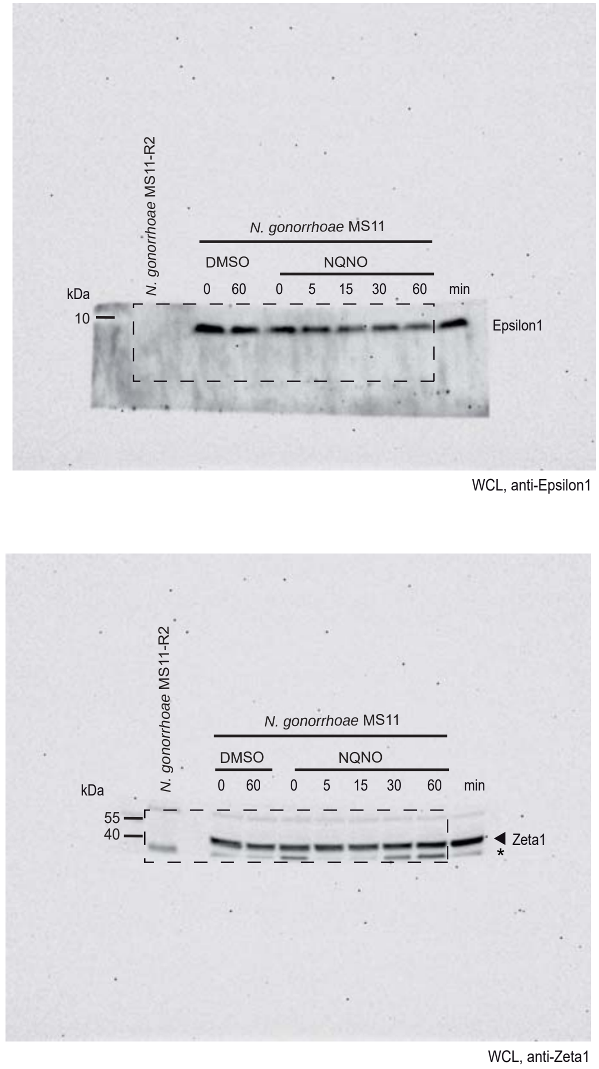

C

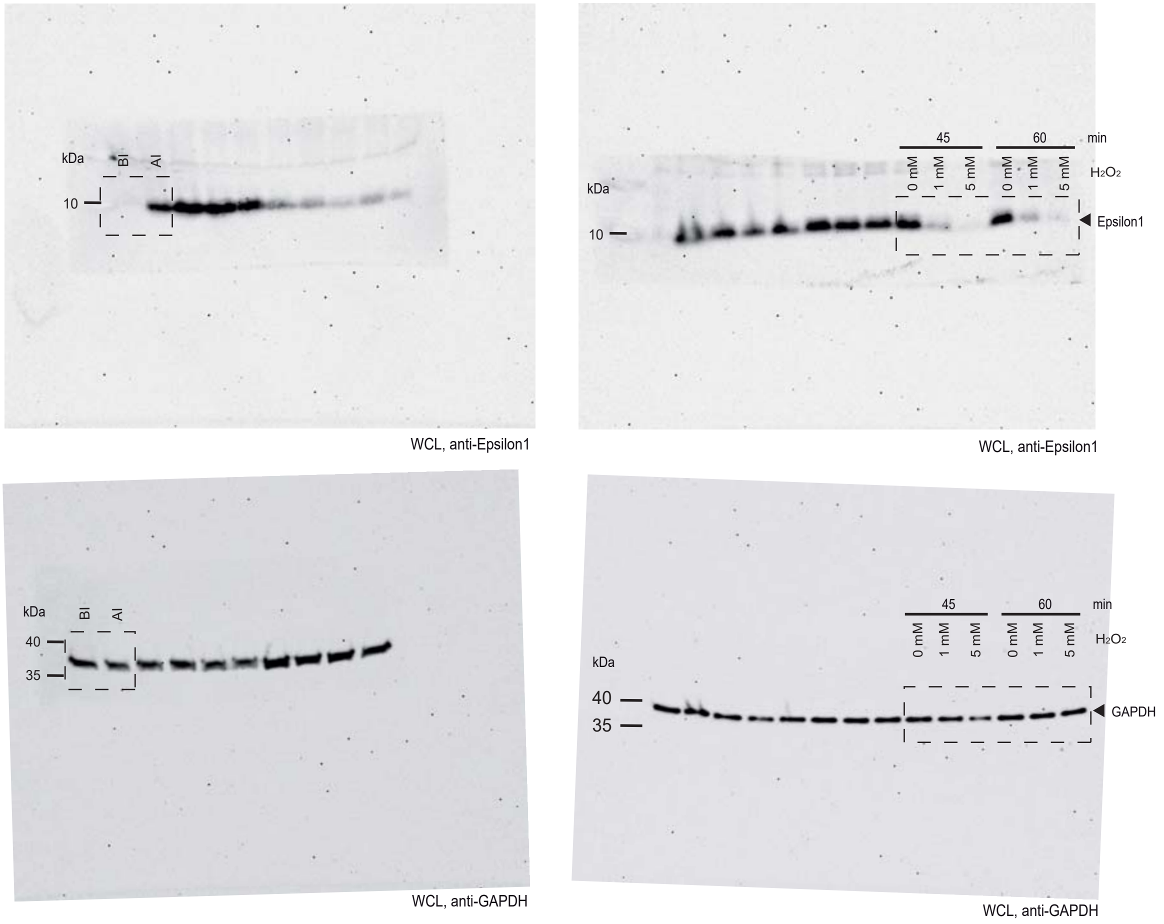

Blots zu Figure 4

D

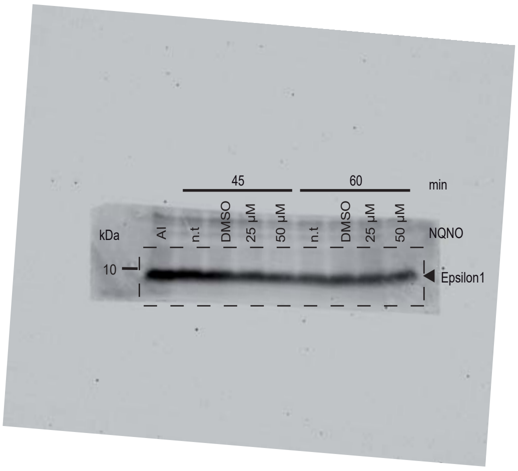

WCL, anti-Epsilon1

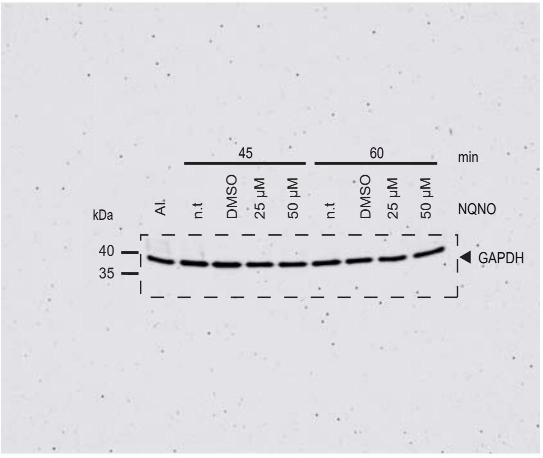

WCL, anti-GAPDH

F

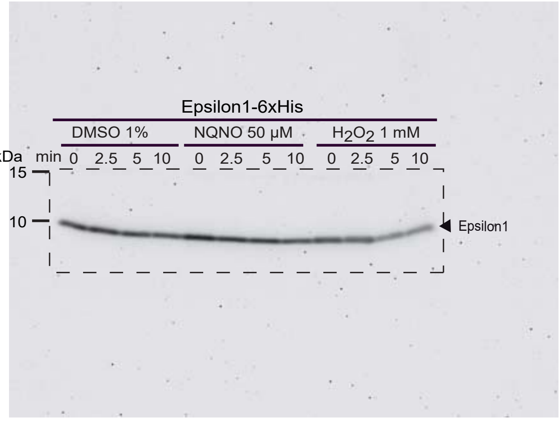

WCL, anti-6xHIS

G

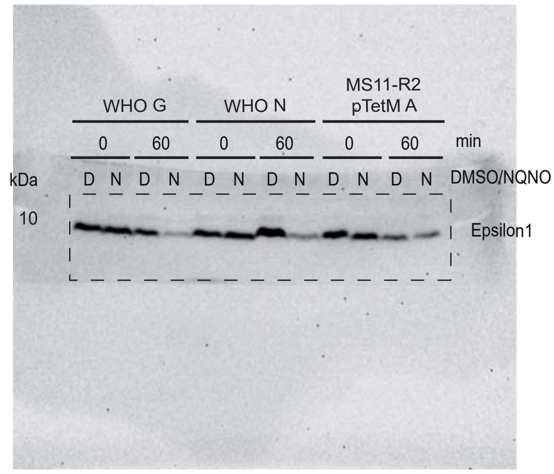

WCL, anti-Epsilon1

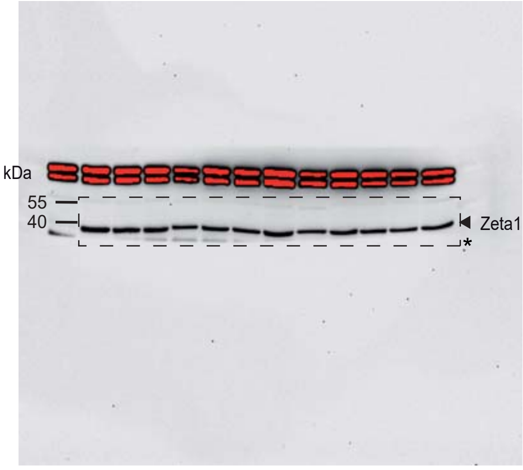

WCL, anti-Zeta1

H

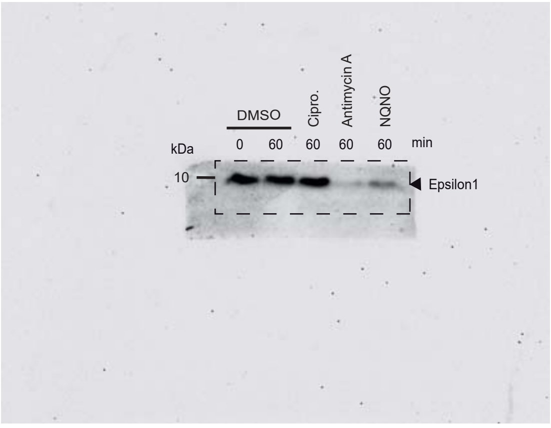

WCL, anti-Epsilon1

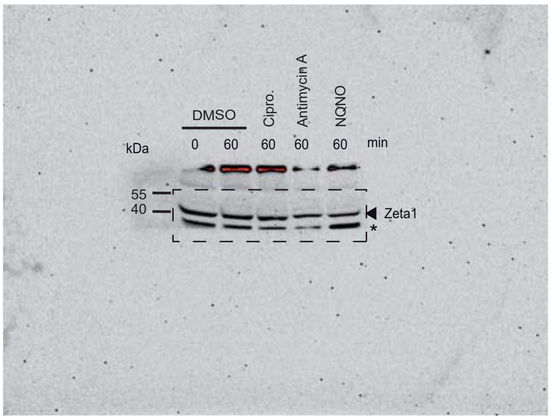

WCL, anti-Zeta1
