# Supplementary figures and images for: A quinolone N-oxide antibiotic selectively targets Neisseria gonorrhoeae via its toxin–antitoxin system
Source: Nat Microbiol. 2025 Apr 2;10(4):939–57. doi: 10.1038/s41564-025-01968-y (PMC11964940; doi:10.1038/s41564-025-01968-y)

D

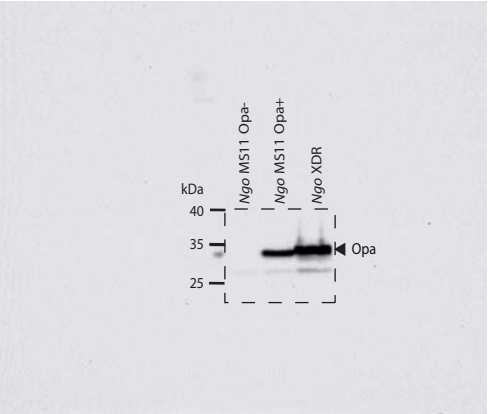

WCL, anti-Opa

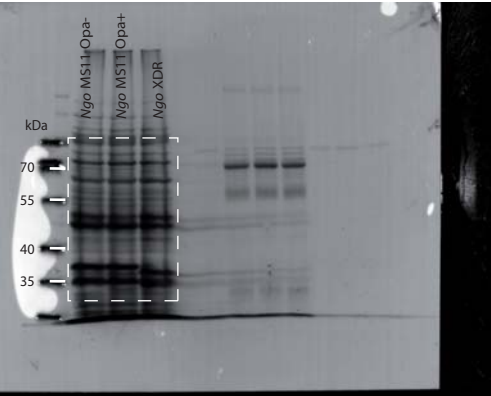

Coomassie

E

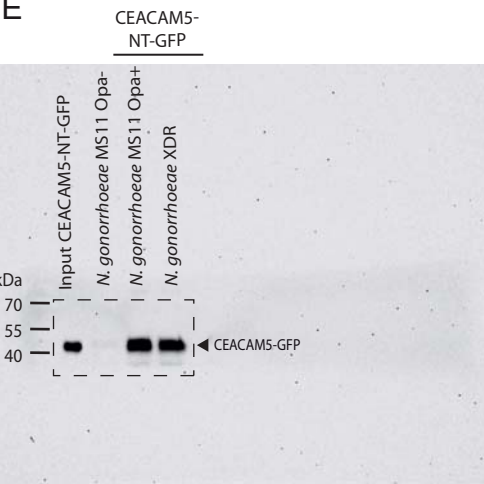

WCL, anti-GFP

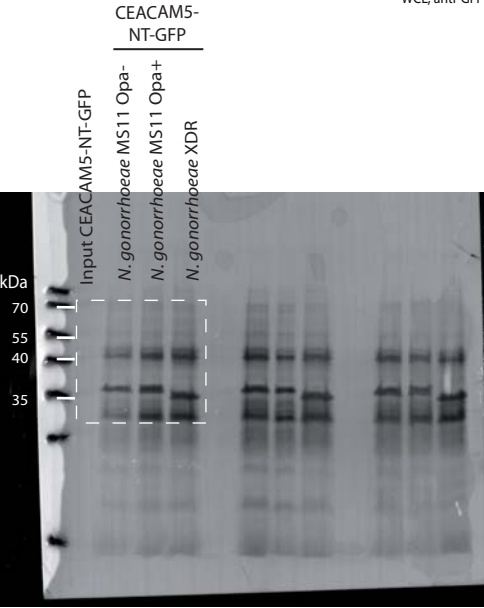

Coomassie

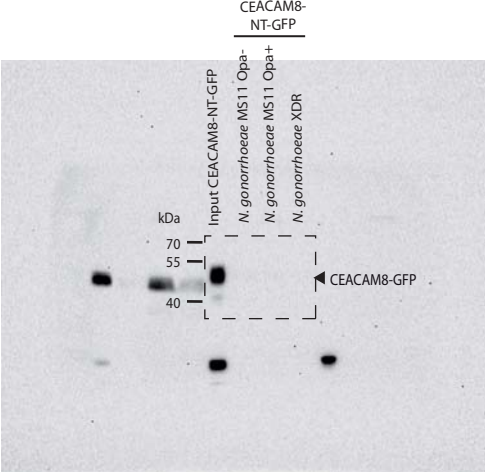

WCL, anti-GFP

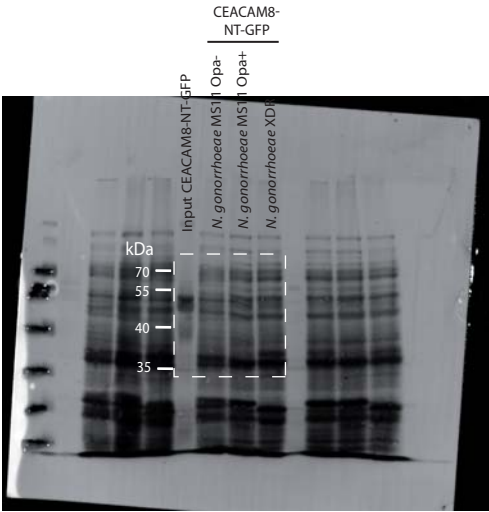

Coomassie

Supplement: Supplementary file 8 — Unprocessed, labelled western blots. [file 41564_2025_1968_MOESM8_ESM.pdf]

Blots for Supplementary Figure 7

C

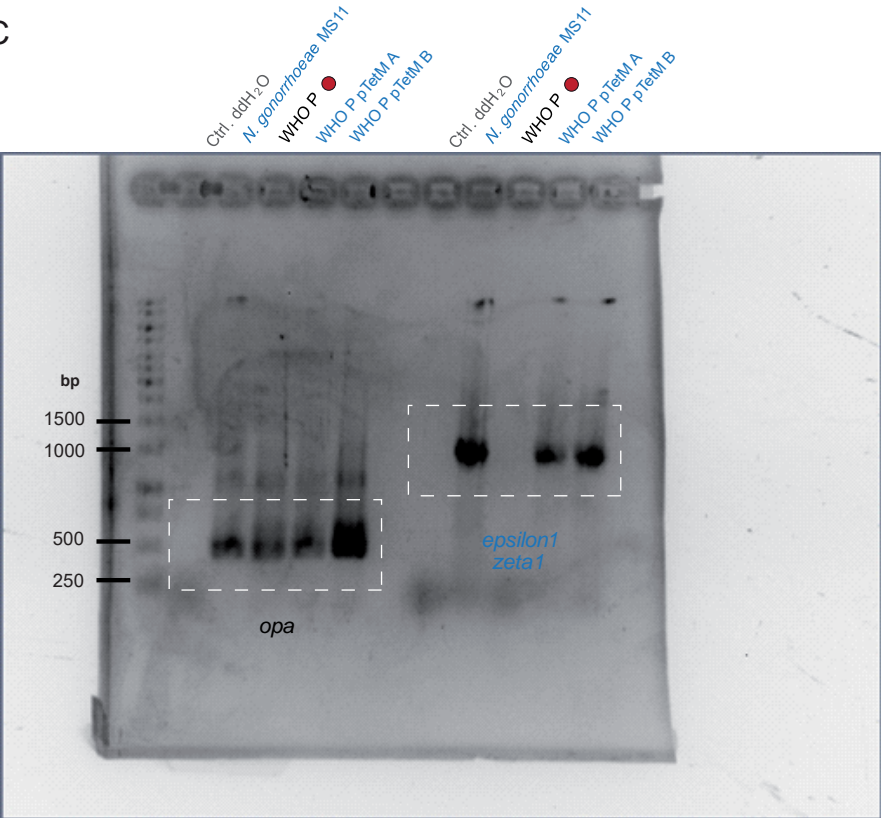

1,2% agarosegel

D

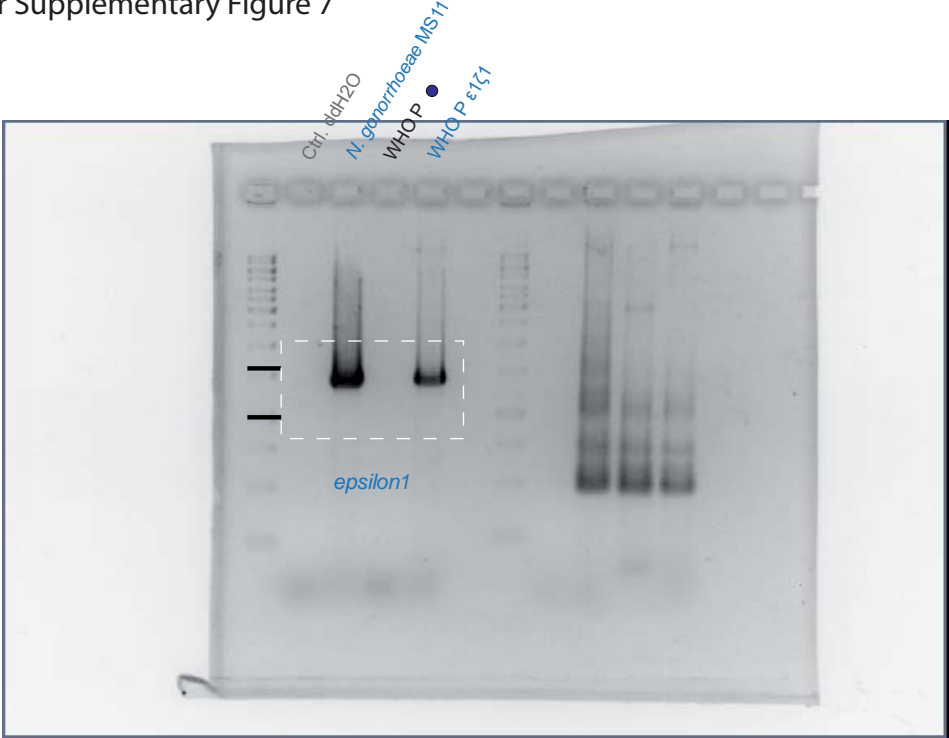

1,2% agarosegel

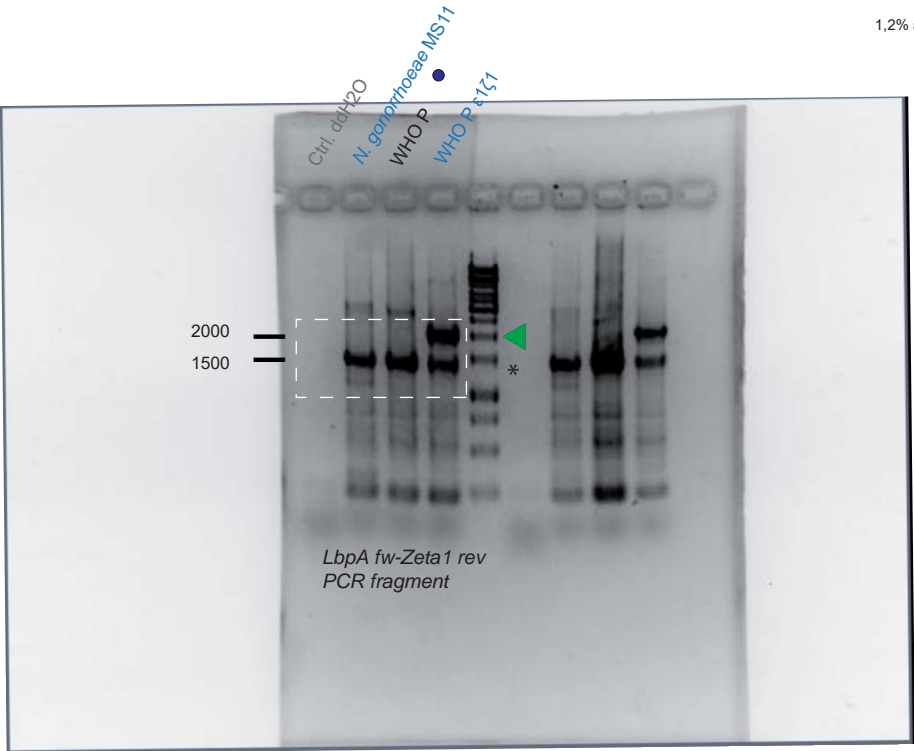

1,2% agarosegel

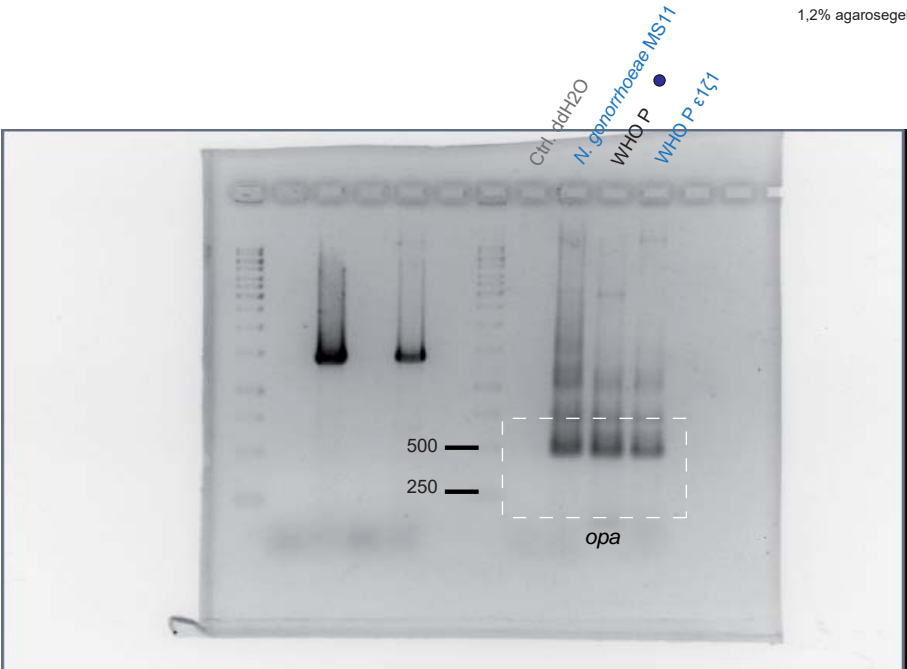

1,2% agarosegel

Supplement: Supplementary file 9 — Unprocessed, labelled agarose gels (c,d). [file 41564_2025_1968_MOESM9_ESM.pdf]
